# Genetic estimation of correlations and causalities between multifaceted modifiable factors and gastro-oesophageal reflux disease

**DOI:** 10.3389/fnut.2022.1009122

**Published:** 2022-11-01

**Authors:** Yuanlin Sun, Xueyuan Cao, Donghui Cao, Yingnan Cui, Kaisheng Su, Zhifang Jia, Yanhua Wu, Jing Jiang

**Affiliations:** ^1^Department of Gastric and Colorectal Surgery, General Surgery Center, The First Hospital of Jilin University, Changchun, Jilin, China; ^2^Department of Clinical Epidemiology, The First Hospital of Jilin University, Changchun, Jilin, China

**Keywords:** Mendelian randomization, gastro-oesophageal reflux disease, modifiable factors, meta-analysis, discovery phase, replication phase

## Abstract

**Background:**

Gastro-oesophageal reflux disease (GORD) is a common gastrointestinal dysfunction that significantly affects the quality of daily life, and health interventions are challenging to prevent the risk of GORD. In this study, we used Mendelian randomization framework to genetically determine the causal associations between multifaceted modifiable factors and the risk of GORD.

**Materials and methods:**

Sixty-six exposures with available instrumental variables (IVs) across 6 modifiable pathways were included in the univariable MR analysis (UVMR). Summary-level genome-wide association studies (GWAS) datasets for GORD were retrieved from the Neale Lab (GORD_*Neale*_, Ncases = 29975, Ncontrols = 390556) and FinnGen (GORD_*Finn*_, Ncases = 13141, Ncontrols = 89695). Using the METAL software, meta-analysis for single nucleotide polymorphisms (SNPs) from GORD_*Neale*_ and GORD_*Finn*_ was conducted with an inverse variance weighted (IVW) fixed-effect model. Moreover, we leveraged partition around medoids (PAM) clustering algorithm to cluster genetic correlation subtypes, whose hub exposures were conditioned for multivariable MR (MVMR) analyses. *P*-values were adjusted with Bonferroni multiple comparisons.

**Results:**

Significant causal associations were identified between 26 exposures (15 risk exposures and 11 protective exposures) and the risk of GORD. Among them, 13 risk exposures [lifetime smoking, cigarette consumption, insomnia, short sleep, leisure sedentary behavior (TV watching), body mass index (BMI), body fat percentage, whole body fat mass, visceral adipose tissue, waist circumference, hip circumference, major depressive disorder, and anxious feeling], and 10 protective exposures (leisure sedentary behavior (computer use), sitting height, hand grip strength (left and right), birth weight, life satisfaction, positive affect, income, educational attainment, and intelligence) showed novel significant causal associations with the risk of GORD. Moreover, 13 exposures still demonstrated independent associations with the risk of GORD following MVMR analyses conditioned for hub exposures (educational attainment, smoking initiation and BMI). In addition, 12 exposures showed suggestive causal associations with the risk of GORD.

**Conclusion:**

This study systematically elucidated the modifiable factors causally associated with the risk of GORD from multifaceted perspectives, which provided implications for prevention and treatment of GORD.

## Introduction

As a common digestive system disease, GORD clinically presents with intraesophageal symptoms, such as acid regurgitation, heartburn and chest pain, and extraesophageal symptoms, including bronchial asthma, chronic cough, and hoarseness (1). Long-term abnormal reflux also increases the risk of pathological oesophageal stricture or even oesophageal adenocarcinoma (2). Currently, the highest prevalence of GORD is observed in North and Central America, followed by Europe (3). GORD symptoms can be influenced by multiple determinants, such as excessive acid exposure caused by anatomical or physiological defects of the oesophagogastric junction, the frequencies of reflux attacks and the acidity of reflux fluid (4). Moreover, poor health status and lifestyle, such as obesity (5, 6), smoking (6, 7), and sleep disorders (8, 9), are common precipitating factors that unilaterally or mutually affect the risk of GORD. However, there is insufficient evidence to determine the causal relationship between each factor and the risk of GORD due to limited studies, potential confounding factors and reverse causalities. In this case, it is of great significance to clarify the risk factors and protective factors related to the causations of GORD in all aspects for the prevention, clinical diagnosis and the treatment of GORD.

MR mainly relies on IVs to detect and quantify causality between exposure and outcome (10). This design overcome the effects of the potential residual confounders and the reverse causalities (11). With the development of the genome-wide association studies (GWAS) based on large sample sizes (12), MR analysis offers a more accurate and representative assessment of the causal associations between exposures and outcomes by primarily using genetic variants. Firstly, according to the Mendel’s second law, the inheritance of a trait is independent of others with randomness. Therefore, the genetic variants of the offspring will not be disturbed by environmental confounding factors (13). Secondly, the distributions of the genetic variants precede the acquired exposures and outcomes including various diseases, so that the order of the three factors is in line with the causal timing and is not affected by reverse causality (14). Thirdly, genetic variants associated with specific exposures will index lifetime differences and thus produce causal estimates that are unsusceptible to the attenuation by errors (regression dilution bias) (15). To date, central obesity indicators (BMI, waist circumference, hip circumference) and daily habits (smoking and alcohol consumption) have been examined for their causal effects on the risk of GORD using MR analysis (16). However, there have been no studies evaluating factors that are causally associated with the risk of GORD as comprehensively as possible.

In this study, we screened a total of 66 exposures across 6 modifiable pathways (daily habits, health status, nutritional and biochemical biomarkers, nutritional and developmental status, emotional factors, socioeconomic factors) for the purpose of identifying the factors that causally associated with the risk of GORD using MR analysis in the discovery and replication phases. Moreover, a meta-analysis based on the above two-phase determined the factors that were causally associated with the risk of GORD to the greatest extent, and also enhanced the statistical efficacy of MR results in the discovery and replication phases.

## Materials and methods

### Identification of exposures for Mendelian randomization analysis

We retrieved factors from PubMed that were associated with the risk of GORD with the query strategy “((Gastroesophageal Reflux[Mesh] OR GERD[Title/Abstract] OR GORD[Title/Abstract] OR Gastric Acid Reflux [Title/Abstract] OR Gastric Acid Reflux[Title/Abstract] OR Gastro Esophageal Reflux[Title/Abstract] OR Gastro oesophageal Reflux[Title/Abstract] OR Gastroesophageal Reflux[Title/Abstract] OR Gastro-oesophageal Reflux[Title/Abstract] OR Reflux, Gastroesophageal[Title/Abstract] OR Esophageal Reflux [Title/Abstract])) AND ((relative[Title/Abstract] AND risk[Title/Abstract]) OR (relative risk[Text Word]) OR (risks[Text Word])).” Reviews, meta-analyses and population-based studies were included, and we identified 58 modifiable factors (Supplementary Table 1) related to the risk of GORD. We next determined the numerous exposures used for MR analyses according to the definitions of the modifiable factors. (1) Factor “smoking behaviors” corresponded to the exposures: smoking initiation, lifetime smoking, cigarette consumption, age of initiation of smoking, and smoking cessation. (2) Factor “sleep disorders” corresponded to the exposures: insomnia, short sleep, long sleep, daytime sleepiness and daytime napping. (3) Factor “physical activity levels” corresponded to the exposures: leisure sedentary behavior (TV watching), leisure sedentary behavior (computer use), moderate to vigorous physical activity levels, 10+ min vigorous activity, strenuous sports or other exercises. (4) Factor “obesity” corresponded to the exposures: BMI, body fat percentage, whole body fat mass, visceral adipose tissue, waist circumference, hip circumference, and waist-to-hip ratio. (6) Factor “height” was categorized into exposures: standing height and sitting height. (7) Factor “muscle mass” corresponded to the exposures: whole body fat-free mass, hand grip strength (left and right). (8) Factor “positive subjective well-beings” corresponded to the exposures: life satisfaction and positive affect. Sex-specific and disease-specific phenotypes, such as “sex-hormone-related factors” and “common medications,” were excluded. Finally, the 66 exposures (Table 1; Supplementary Table 2) across 6 modifiable pathways were identified to explore causal associations with the risk of GORD.

**TABLE 1:** Information of the 66 exposures included in MR analysis.

Exposure	Source	F^a^	*R*^2^ (%)^b^	N. SNPs^c^	Samples	Unit of measurement	OR at 80% statistical power
**Daily habits**							
Alcohol consumption	(33)	63.901	0.618	99	941280	Drinks per week	0.797/1.210
Coffee consumption	(65)	86.083	0.743	29	335909	Cups of coffee per day	0.814/1.191
Caffeine consumption	(66)	86.910	0.623	28	362316	mg per day	0.797/1.209
Smoking initiation	(33)	44.684	35.082	378	1232091	Events	0.972/1.028
Lifetime smoking	(67)	43.873	1.282	126	462690	Lifetime smoking index	0.858/1.144
Cigarette consumption	(33)	87.547	1.374	55	337334	Cigarettes per day	0.863/1.140
Age of initiation of smoking	(33)	41.386	0.085	10	341427	Years	0.470/1.577
Smoking cessation	(33)	55.325	0.243	24	547219	Events	0.669/1.349
Intake of total sugar	(68)	47.992	0.184	9	235391	Kcal/day	0.619/1.401
Intake of fat	(68)	72.037	0.134	5	268922	Kcal/day	0.557/1.469
Intake of carbohydrate	(68)	38.629	0.144	12	268922	Kcal/day	0.571/1.457
Breakfast skipping	(69)	39.411	0.122	6	193860	Always/sometimes/never	0.571/1.456
Morning person	(70)	45.210	1.289	122	403195	Events	0.853/1.149
Insomnia	(71)	43.037	0.688	41	237627	Events	0.801/1.199
Short sleep	(72)	37.585	0.465	26	411934	Events	0.759/1.241
Long sleep	(72)	38.845	0.091	10	339926	Events	0.492/1.558
Daytime sleepiness	(73)	42.395	0.122	42	452071	Never/sometimes/often/always	0.557/1.480
Daytime napping	(74)	47.129	1.031	106	452633	Never or rarely/sometimes/always	0.837/1.163
Leisure sedentary behavior (TV watching)	(51)	42.762	1.370	141	408815	Hours	0.858/1.146
Leisure sedentary behavior (computer use)	(51)	38.889	0.447	47	408815	Hours	0.752/1.257
Moderate to vigorous physical activity levels	(75)	34.084	0.163	19	377234	MET-minutes/week	0.599/1.430
10+ min vigorous activity	(75)	40.109	0.128	7	261055	Events	0.550/1.486
Strenuous sports or other exercises	(75)	38.306	0.131	14	350492	Events	0.551/1.479
**Health status**							
Childhood-onset asthma	(76)	77.609	2.441	103	314633	Events	0.897/1.104
Adult-onset asthma	(76)	63.801	0.877	46	327253	Events	0.829/1.175
Type 2 diabetes	(77)	55.419	2.128	380	8318130	Events	0.890/1.112
Coronary artery disease	(78)	73.439	3.564	145	296525	Events	0.915/1.086
Atrial fibrillation	(79)	94.914	1.022	111	1030836	Events	0.841/1.162
Ulcerative colitis	(80)	63.050	8.476	39	27432	Events	0.944/1.056
Crohn’s disease	(80)	59.192	14.391	53	20883	Events	0.957/1.043
**Nutritional and biochemical biomarkers**							
Fasting glucose	(81)	125.237	3.020	69	281416	mmol/L	0.904/1.097
Fating insulin	(81)	51.976	0.683	38	281416	pmol/L	0.800/1.205
2-h blood glucose	(81)	57.248	0.264	14	281416	mmol/L	0.683/1.332
Glycosylated hemoglobin (HbA1c)	(81)	103.014	2.632	73	281416	Percentage	0.897/1.105
HDL cholesterol	(82)	110.137	1.134	145	1320016	mg/dL	0.843/1.159
LDL cholesterol	(82)	187.847	1.565	112	1320016	mg/dL	0.867/1.135
Total cholesterol	(82)	166.998	1.644	134	1320016	mg/dL	0.870/1.132
Triglycerides	(82)	130.638	1.138	123	1320016	mg/dL	0.845/1.159
Adiponectin	(83)	57.727	1.373	14	29347	ln(mg/dL)	0.858/1.145
Leptin	(84)	19.552	0.288	6	33987	log(ng/mL)	0.693/1.318
Vitamin C	(85)	89.574	1.715	11	52018	μmol/L	0.873/1.129
25-Hydroxyvitamin D	(86)	112.523	2.623	118	496946	nmol/L	0.897/1.104
**Nutritional and developmental status**							
Body mass index	MRC IEU (34)^d^	62.134	5.896	458	461460	Kg/m^2^	0.931/1.069
Body fat percentage	MRC IEU (34)	58.238	4.841	395	454633	Percentage	0.924/1.077
Whole body fat mass	MRC IEU (34)	61.138	5.626	435	454137	Kg	0.929/1.071
Whole body fat-free mass	MRC IEU (34)	83.701	9.786	556	454850	Kg	0.947/1.054
Visceral adipose tissue	(87)	58.878	3.331	201	325153	Kg	0.909/1.093
Waist circumference	MRC IEU (34)	56.580	4.357	374	462166	cm	0.920/1.081
Hip circumference	MRC IEU (34)	62.533	5.357	420	462117	cm	0.928/1.073
Waist-to-hip ratio	(88)	64.130	3.106	355	697734	Percentage	0.905/1.096
Standing height	MRC IEU (34)	138.651	22.252	773	461950	cm	0.965/1.036
Sitting height	MRC IEU (34)	102.675	12.807	602	461536	cm	0.953/1.047
Hand grip strength (left)	MRC IEU (34)	47.127	1.564	157	461026	Kg	0.866/1.135
Hand grip strength (right)	MRC IEU (34)	47.585	1.734	176	461089	Kg	0.873/1.129
Birth weight	(89)	53.403	3.044	178	298142	Kg	0.904/1.097
Childhood BMI	(90)	50.951	2.635	18	34744	Kg/m^2	0.897/1.104
**Emotional factors**							
Major depressive disorder	(91)	38.704	0.503	50	361315	Events	0.766/1.240
Anxious feeling	(92)	35.456	0.210	23	371318	Events	0.646/1.377
Schizophrenia	(93)	21.293	10.819	189	320404	Events	0.951/1.050
Bipolar disorder	(94)	22.702	0.452	53	413466	Events	0.765/1.246
Anorexia nervosa	(95)	170.613	0.321	6	72517	Events	0.720/1.290
Life satisfaction	(96)	39.714	3.682	80	80852	Life satisfaction score	0.913/1.088
Positive affect	(96)	40.991	0.900	98	410063	Positive affect score	0.824/1.179
**Socioeconomic factors**							
Income	MRC IEU (34)	40.958	0.443	48	397751	Dollars	0.751/1.256
Educational attainment	(32)	46.668	7.350	317	766345	Years	0.938/1.062
Intelligence	(97)	42.540	2.616	167	269867	*g* phenotype	0.989/1.011

^a^F, F statistics, the indicator of evaluating weak IV bias.

^b^R^2^ (%), genetic explanations of phenotypic variance.

^c^N. SNPs, number of single nucleotide polymorphisms.

^d^MRC-IEU, the MRC IEU OpenGWAS data infrastructure.

### Data sources of gastro-oesophageal reflux disease

Neale Lab^1^ performed GWAS across more than 7,000 phenotypes from 6 ethnic groups (European, East Asian, African, American, Central South Asian, and Middle Eastern) with the UK Biobank (UKB) approval. Similarly, FinnGen^2^ is a large public consortium for genetic and health data from Finnish participants. The summary-level GWAS datasets in the Neale Lab and FinnGen consortiums are free and publicly available.

For the outcome cohort, the ‘‘K21 Gastro-oesophageal reflux disease’’ GWAS dataset obtained from the Neale Lab (GORD_*Neale*_)^3^ was used for the discovery phase. GORD_*Neale*_ GWAS dataset, which contained 29975 cases and 390556 controls and was adjusted for covariates (age, sex, age*sex, age^2, age^2*sex and the first 10 principal components), was analyzed with Scalable and Accurate Implementation of Generalized mixed model (SAIGE) (17), and implemented in the hail batch.^4^ In the replication phase, the “finngen_R6_K11_REFLUX” GWAS dataset (13141 cases and 189695 controls) obtained from FinnGen (GORD_*Finn*_) was analyzed using SAIGE (17)^5^ and adjusted for covariates including age, sex, 10 principal components, and genotyping batch. Moreover, to effectively complement and identify the causalities between the 66 exposures and the risk of GORD, we employed a sample-size-weighted approach with METAL (18) to perform the IVW fixed-effect meta-analysis (GORD_*meta*_) for the SNPs of summary-level GORD_*Neale*_ and GORD_*FinnGen*_ datasets.

### Selection of instrumental variables

In this study, all IV data derived from European-descent GWAS datasets followed the MR principles. Namely, IVs should be closely related to exposures; IVs cannot affect outcomes directly but rather through exposures, and no associations were found between IVs and known or unknown confounders. Moreover, genome-wide statistically significant SNPs (*P* < 5*10^–8^) without linkage disequilibrium (*R*^2^ > 0.001, clumping Kb < 10000) were used as independent IVs. Meanwhile, the effect values of exposures and outcomes were harmonized to the same effect allele to ensure accurate MR analysis (19).

### Estimation of heritability and genetic correlation

The linkage disequilibrium score regression (LDSC) is an effective method for estimating heritability (*h*^2^_*snp*_) and genetic correlations (*r*_*g*_) between different phenotypes based on summary-level GWAS datasets (20). According to the European population-characterized LD score profiles of the 1000GenomesProjects^6^ (21), we calculated the heritability using LDSC for 66 exposures in 6 modifiable pathways, and identified 2,145 (66 exposures*32.5) genetic correlations between 66 exposures (including self-diagnosis), as well as genetic correlations between 66 exposures and GORD_*Neale*_ and GORD_*Finn*_. *P* ≤ 0.05 was considered as the threshold of statistical significance for genetic correlations. *P* ≤ 2.261E-05 (0.05/2145, Bonferroni multiple comparisons) was considered as the threshold of genetic correlation with significantly statistical significance.

### Univariable Mendelian randomization analysis

**FIGURE 1:**
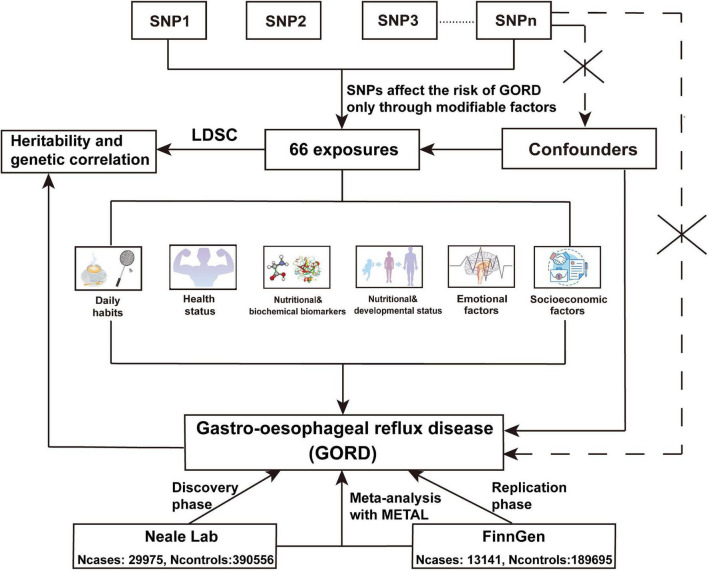
The flow chart of the UVMR analyses. In UVMR analysis, we assessed the causal associations between 66 exposures across 6 modifiable pathways and the risk of GORD from discovery phase, replication phase, and IVW fixed-effect meta-analysis using METAL for SNPs from GORD_*Neale*_ and GORD_*Finn*_. The selection of IVs (SNPs) abided by 3 assumptions of MR analysis. (1) IVs should be closely related to exposure; (2) IVs cannot affect outcome directly, but only through exposure; (3) IVs should not be associated with the known or unknown confounders.

The process of UVMR is shown in Figure 1. In this study, the two-sample UVMR was performed using the “TwoSampleMR” package of R software. The IVW method was principally employed to explore the causality between each exposure and the risk of GORD, with the weighted median method and MR-Egger method for enhancing the findings. Under the premise of ensuring that all IVs are valid, the IVW method uses the reciprocal of the variance of each IV as a weight to calculate the causal estimate of a single IV, and the causal estimate corresponding to each IV can be summed into a weighted estimate as a whole (22, 23). The weighted median estimate is the median of the distribution of all IV estimates sorted by weight, and the weight of each IV causal estimate depends on the accuracy of the estimation. In cases where at least half of SNPs are valid IVs, the weighted median provides a consistent estimate of the final effect (24). The MR-Egger method does not force the regression line to pass through the origin, allowing the included IVs to have directional pleiotropy. When the intercept of MR-Egger analysis is significantly different from 0, it indicates that there is directional gene pleiotropy; when the intercept of the regression is 0, or the intercept is not statistically significant (*P* > 0.05), the slope of MR-Egger represents the causal estimate of exposure on the outcome (25). Therefore, the MR-Egger method can be utilized to examine and correct the horizontal pleiotropy. MR-PRESSO with the “MRPRESSO package” of R software was applied to detect outliers (26). In this study, when outliers were detected, they would be eliminated, and MR analysis would be reconducted until no outliers remained. Causal associations with *P* ≤ 7.576E-04 (0.05/66) were deemed as significant, and causal associations with 7.576E-04 < *P* ≤ 0.05 were defined as suggestive causal associations.

### Genetic correlation clustering and multivariable Mendelian randomization analysis

**FIGURE 2:**
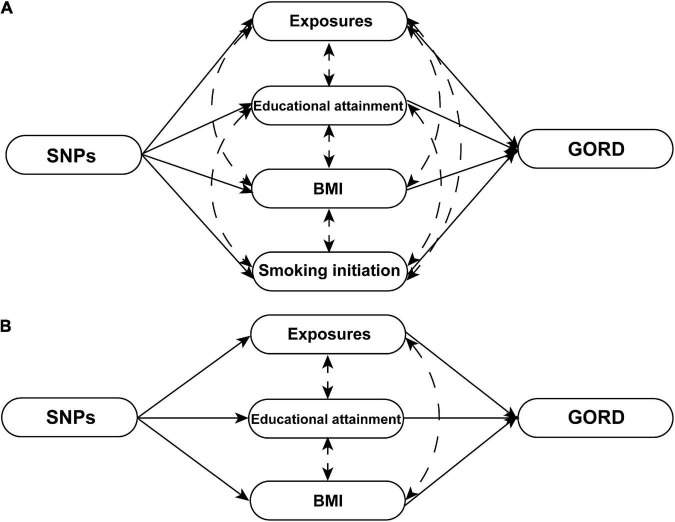
The schematics of MVMR analyses. **(A)** The schematic of MVMR analyses with adjustment of educational attainment, smoking initiation, and BMI for 11 significant causal exposures [cigarette consumption, insomnia, short sleep, leisure sedentary behavior (TV watching), waist-to-hip ratio, height (standing and sitting), major depressive disorder, anxious feeling, life satisfaction, and positive affect]. **(B)** The schematic of MVMR analyses with adjustment of educational attainment and BMI for four significant causal exposures [leisure sedentary behavior (computer use), hand grip strength (left and right), and birth weight]. Exposures that respectively strongly genetically correlated with the BMI (body fat percentage, whole body fat mass, visceral adipose tissue, and waist circumference), smoking initiation (lifetime smoking), and educational attainment (income and intelligence) were excluded from MVMR analyses.

We performed the PAM clustering algorithm to cluster genetic correlation subgroups for exposures that were identified to be significantly causally associated with the risk of GORD (*P* ≤ 7.576E-04). A vital advantage of the PAM clustering algorithm included the ability to robustly cluster different data types within limited samples, minimizing the influences of data noise and isolated points on the clustering results. Inter-exposure similarity was measured using Euclidean distance, 80% of the exposures were taken at one time, 1,000 replicate samplings were conducted, and the slope of the CDF curve and area under the CDF curve determined the most appropriate number of clusters to be placed. Exposures that were genetically correlated with all other exposures with statistical significance (*P* ≤ 0.05) in each cluster were defined as the hub exposures. MVMR principle hypothesizes that genetic-level links existed in individual exposures, and that SNPs are strongly associated with at least one of these exposures (27). Thus, as shown in Figure 2, to further clarify whether significant causal exposures were directly causally associated with the risk of GORD rather than being mediated by hub exposures, we chose significant causal exposures that had statistically significant genetic correlations with hub exposures for MVMR, which also included SNPs that were identified as statistically significant from the genome-wide level with at least one specific exposure (*P* < 5*10^–8^) to be IVs and GORD_*meta*_ GWAS dataset as the source of outcome GWAS dataset. MVMR-IVW method (27) and MVMR-MR Egger method (28) were employed for MVMR analyses. Horizontal pleiotropy was examined by the MR-Egger intercept test.

### Statistical analysis

Weak IV bias was evaluated using the *F* statistic [*F* statistic = (Beta/SE)^2^]. The general assumption is that there are no weak instrumental variable biases when the SNPs *F* statistics are greater than 10 (29), and in this study, the SNPs *F* statistics less than or equal to 10 were removed from MR analysis. The Cochran’s *Q* test *P*-value and *I*^2^ were employed to evaluate and quantify the heterogeneity of SNPs. In this study, the multiplicative random effects model was adopted for MR analysis with Cochran’s *Q* test *P* ≤ 0.05, and the fixed-effect model was utilized to conduct MR analysis with Cochran’s *Q* test *P* > 0.05 (30). When the number of SNPs involved in MR analysis was less than or equal to 3, the fixed-effect model was applied for MR analysis. The mRnd online tool was used to calculate statistical power of MR analysis^7^ (31). In this study, all data analysis and visualization were completed using R software version 4.1.2.

## Results

### Overall heritability and genetic correlation of the 66 exposures

**FIGURE 3:**
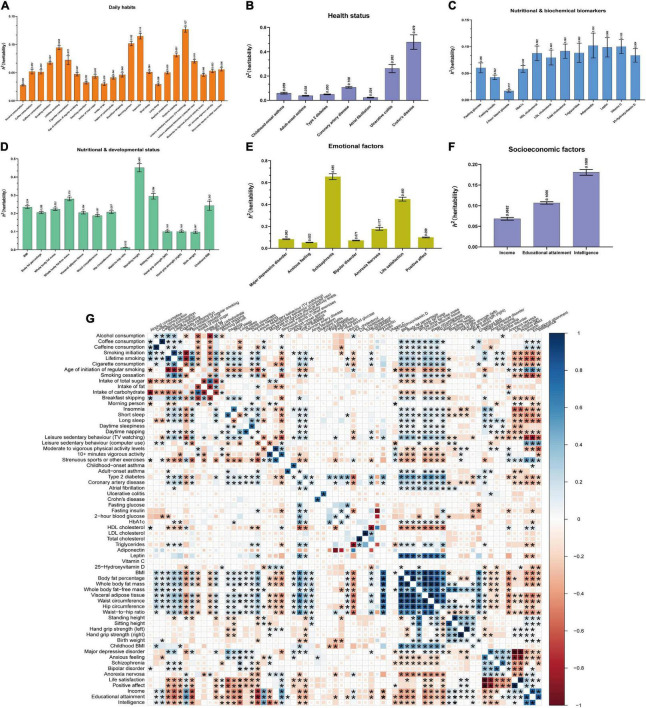
Estimations of heritability and genetic correlations for 66 exposures using LDSC. **(A–F)** Estimations of heritability for 66 exposures across 6 modifiable pathways (daily habits, health status, nutritional and biochemical biomarkers, nutritional and developmental status, emotional factors, and socioeconomic factors). Mean with SEM was represented with error bar. **(G)** Genetic correlations between 66 exposures. Totally, there were 2,145 (66 exposures*32.5) genetic correlations among 66 exposures. The sizes and colors of squares respectively represented the significance level and genetic correlation directions. If *P* ≤ 0.05 (the threshold of genetic correlation with statistical significance), the squares would be full-sized. *P* ≤ 2.331E-05 (0.05/2145, Bonferroni multiple comparisons) was considered as the threshold of genetic correlation with significantly statistical significance between two exposures, whose squares would be showed with asterisk.

We estimated heritability and genetic correlation using LDSC for 66 exposures, GORD_*Neale*_ and GORD_*Finn*_ (Figures 3A–F; Supplementary Table 3). Total observed scale heritability ranged from the lowest waist-to-hip ratio (*h*^2^_*snp*_ = 0.011, SE = 4.000E-03) to highest schizophrenia (*h*^2^_*snp*_ = 0.655, SE = 0.024). GORD_*Neale*_ (*h*^2^_*snp*_ = 0.018, SE = 1.500E-03) and GORD_*Finn*_ (*h*^2^_*snp*_ = 0.015, SE = 2.500E-03) possessed largely consistent heritability. The exposure-exposure, exposure-GORD_*Neale*_, exposure-GORD_*Finn*_, and GORD_*Neale*_-GORD_*Finn*_ genetic correlations were shown in Figure 3G and Table 2. Particularly, GORD_*Neale*_ and GORD_*Finn*_ shared a high degree of genetic correlation (*r*_*g*_ = 0.929, *P* = 3.389E-21), and 39 of 48 exposures that showed a statistically significant genetic correlation with GORD_*Neale*_ also had a similar pattern of genetic correlation with GORD_*Finn*_.

**TABLE 2:** Statistically significant genetic correlation between 48 exposures and GORD_*Neale*_ as well as GORD_*Finn*_.

Exposure/Outcome	GORD_*Neale*_^a^				GORD_*Finn*_^b^			
		
	*r*_*g*_ ^c^	SE	Z-score	*P*-valu*e*	*r*_*g*_	SE	Z-score	*P*-valu*e*
Coffee consumption	−0.091	0.040	−2.255	0.024	−0.055	0.055	−1.006	0.315
Caffeine consumption	−0.161	0.041	−3.936	8.274E-05	−0.199	0.060	−3.313	9.216E-04
Smoking initiation	0.342	0.031	10.904	1.103E-27	0.219	0.051	4.326	1.518E-05
Lifetime smoking	0.438	0.033	13.212	7.499E-40	0.269	0.049	5.469	4.540E-08
Cigarette consumption	0.326	0.038	8.500	1.893E-17	0.275	0.058	4.741	2.125E-06
Age of initiation of regular smoking	−0.401	0.044	−9.049	1.445E-19	−0.386	0.072	−5.385	7.254E-08
Smoking cessation	0.341	0.047	7.221	5.145E-13	0.185	0.070	2.629	8.573E-03
Breakfast skipping	0.238	0.056	4.286	1.823E-05	0.161	0.072	2.118	0.033
Insomnia	0.465	0.037	12.623	1.587E-36	0.397	0.054	7.298	2.932E-13
Short sleep	0.384	0.042	9.225	2.827E-20	0.257	0.050	5.100	3.401E-07
Long sleep	0.202	0.049	4.133	3.587E-05	0.191	0.068	2.820	4.797E-03
Daytime sleepiness	0.124	0.041	3.052	2.276E-03	0.182	0.059	3.099	1.940E-03
Daytime napping	0.160	0.037	4.288	1.804E-05	0.159	0.046	3.484	4.938E-04
Leisure sedentary behavior (TV watching)	0.393	0.036	11.039	2.492E-28	0.259	0.057	4.577	4.715E-06
Leisure sedentary behavior (computer use)	−0.157	0.032	−4.943	7.691E-07	−0.140	0.049	−2.855	4.299E-03
10+ min vigorous activity	−0.104	0.043	−2.430	0.015	−0.120	0.060	−1.995	0.046
Strenuous sports or other exercises	−0.374	0.040	−9.369	7.346E-21	−0.252	0.056	−4.474	7.670E-06
Adult-onset asthma	0.292	0.040	7.315	2.573E-13	0.121	0.068	2.496	0.012
Type 2 diabetes	0.301	0.034	8.918	4.744E-19	0.256	0.045	5.712	1.119E-08
Coronary artery disease	0.311	0.032	9.646	5.115E-22	0.173	0.043	3.974	7.079E-05
Atrial fibrillation	0.091	0.042	2.191	0.028	0.063	0.052	1.221	0.222
Fasting glucose	0.136	0.050	2.743	6.088E-03	−0.004	0.065	−0.061	0.951
Fating insulin	0.121	0.060	2.028	0.043	0.117	0.071	1.636	0.102
HDL cholesterol	−0.228	0.043	−5.342	9.189E-08	−0.180	0.073	−2.471	0.013
LDL cholesterol	0.131	0.059	2.228	0.026	0.050	0.081	0.615	0.538
Triglycerides	0.214	0.044	4.901	9.526E-07	0.235	0.060	3.921	8.830E-05
Leptin	0.180	0.081	2.220	0.026	0.104	0.112	0.926	0.354
25-Hydroxyvitamin D	0.078	0.032	2.450	0.014	0.017	0.041	0.425	0.671
Body mass index	0.369	0.027	13.826	1.768E-43	0.183	0.045	4.052	5.083E-05
Body fat percentage	0.393	0.027	14.714	5.279E-49	0.214	0.046	4.669	3.024E-06
Whole body fat mass	0.347	0.026	13.404	5.758E-41	0.168	0.045	3.749	1.772E-04
Whole body fat-free mass	0.067	0.025	2.662	7.768E-03	−0.021	0.040	−0.521	0.603
Visceral adipose tissue	0.422	0.028	15.286	9.440E-53	0.203	0.050	4.073	4.633E-05
Waist circumference	0.372	0.027	13.767	4.005E-43	0.186	0.046	4.029	5.600E-05
Hip circumference	0.234	0.025	9.202	3.516E-20	0.089	0.043	2.049	0.040
Waist-to-hip ratio	0.399	0.028	14.391	5.876E-47	0.218	0.041	5.344	9.089E-08
Standing height	−0.147	0.027	−5.462	4.716E-08	−0.134	0.038	−3.549	3.871E-04
Sitting height	−0.129	0.028	−4.619	3.852E-06	−0.114	0.037	−3.063	2.194E-03
Hand grip strength (left)	−0.159	0.031	−5.159	2.487E-07	−0.179	0.046	−3.918	8.919E-05
Hand grip strength (right)	−0.157	0.030	−5.259	1.450E-07	−0.201	0.046	−4.362	1.291E-05
Birth weight	−0.127	0.034	−3.704	2.126E-04	−0.066	0.054	−1.228	0.219
Major depressive disorder	0.462	0.034	13.594	4.338E-42	0.531	0.055	9.607	7.438E-22
Anxious feeling	0.247	0.041	6.048	1.470E-09	0.369	0.059	6.303	2.919E-10
Life satisfaction	−0.356	0.036	−9.839	7.687E-23	−0.399	0.053	−7.484	7.199E-14
Positive affect	−0.337	0.036	−9.398	5.542E-21	−0.392	0.053	−7.403	1.336E-13
Income	−0.421	0.037	−11.484	1.580E-30	−0.284	0.054	−5.215	1.841E-07
Educational attainment	−0.484	0.027	−18.058	6.810E-73	−0.334	0.046	−7.257	3.967E-13
Intelligence	−0.386	0.032	−12.078	1.383E-33	−0.195	0.047	−4.120	3.788E-05
GORD_*Neale*_	1.000	8.140E-07	1.229E + 06	0.000	0.929	0.098	9.450	3.389E-21
GORD_*Finn*_	0.929	0.098	9.450	3.389E-21	1.000	1.567E-05	6.383E + 04	0.000

^a^GORD_Neale_, summary-level GWAS dataset of GORD retrieved from Neale Lab.

^b^GORD_Finn_, summary-level GWAS dataset of GORD retrieved from FinnGen.

^c^r_g_, genetic correlation.

### Univariable Mendelian randomization analysis in the discovery phase and replication phase

**FIGURE 4:**
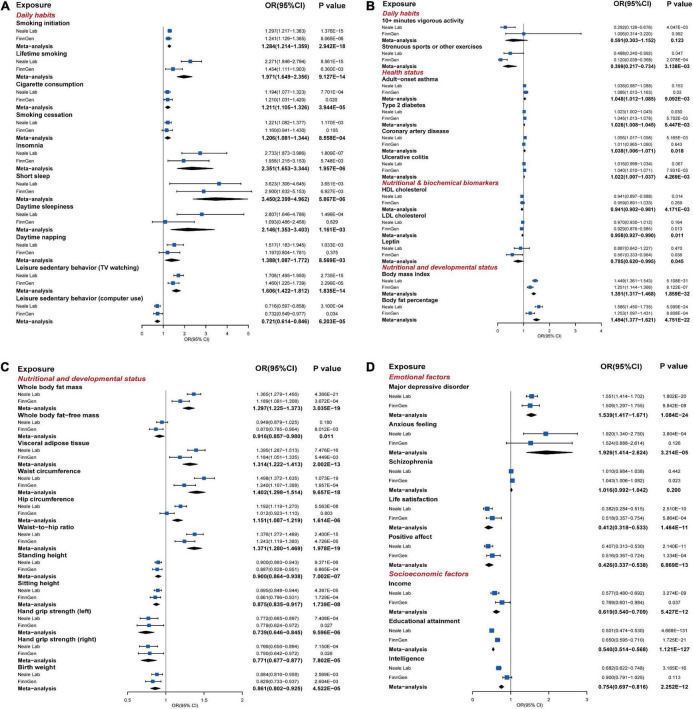
The statistically significant results of MR analysis in the discovery phase or replication phase and meta-analysis (*P* ≤ 0.05). **(A–D)** The forest plots graphically illustrated the aggregated results for 40 exposures that have statistically significant causal associations with the risk of GORD based on the IVW method in the discovery phase, replication phase, or meta-analysis, where “Neale Lab,” “FinnGen,” and “Meta-analysis,” respectively, denoted the MR results in the discovery phase, replication phase, and meta-analysis. The causal effects were represented as odds ratios (ORs) with IVW method (blue box). The MR results of the discovery phase, replication phase, and meta-analysis with IVW method, weighted median method, MR-Egger method, and MR-PRESSO method were summarized in Supplementary Tables 4–6 in detail.

In the discovery and replication phases, all causal associations were examined by the MR-PRESSO method, and outliers were eliminated. In the discovery phase, based on the IVW method, a total of 34 exposures (Figure 4; Supplementary Table 4) were causally associated with the risk of GORD with statistical significance (*P* ≤ 0.05). Among them, the following 20 exposures were causally associated with an increased risk of GORD: smoking initiation, lifetime smoking, cigarette consumption, smoking cessation, insomnia, short sleep, daytime sleepiness, daytime napping, leisure sedentary behavior (TV watching), type 2 diabetes, coronary artery disease, BMI, body fat percentage, whole body fat mass, visceral adipose tissue, waist circumference, hip circumference, waist-to-hip ratio, major depressive disorder, and anxious feeling. The other 14 exposures showed causal associations with a decreased risk of GORD: leisure sedentary behavior (computer use), 10+ min vigorous activity, strenuous sports or other exercises, height (standing and sitting), hand grip strength (left and right), birth weight, life satisfaction, positive affect, income, educational attainment, and intelligence. Moreover, heterogeneities were observed in the SNPs corresponding to the following exposures that causally were associated with the risk of GORD (*P* ≤ 0.05): smoking initiation, lifetime smoking, insomnia, short sleep, daytime sleepiness, daytime napping, leisure sedentary behavior (TV watching), BMI, body fat percentage, whole body fat mass, visceral adipose tissue, waist circumference, hip circumference, waist-to-hip ratio, height (standing and sitting), hand grip strength (left and right), birth weight, anxious feeling, life satisfaction, positive affect, income, educational attainment, and intelligence (Supplementary Figures 1–5). Furthermore, no horizontal pleiotropy was detected in the causal association between the exposures and the risk of GORD.

In the replication phase (Figure 4; Supplementary Table 5), a total of 29 exposures showed causal associations with the risk of GORD (*P* ≤ 0.05), among which, the causal associations between the following 25 exposures were consistent with the results in the discovery phase: smoking initiation, lifetime smoking, cigarette consumption, insomnia, short sleep, leisure sedentary behaviors (TV watching and computer use), strenuous sports or other exercises, type 2 diabetes, BMI, body fat percentage, whole body fat mass, visceral adipose tissue, waist circumference, waist-to-hip ratio, height (standing and sitting), hand grip strength (left and right), birth weight, major depressive disorder, life satisfaction, positive affect, income, and educational attainment. Meanwhile, exposures “child-onset asthma,” “ulcerative colitis,” “whole body fat-free mass,” and “schizophrenia,” which had not previously shown causal associations with the risk of GORD in the discovery phase, also showed causal associations with the risk of GORD in the replication phase. SNPs in the educational attainment, BMI, smoking initiation, sitting height, waist circumference, whole body fat mass, standing height, body fat percentage, birth weight, type 2 diabetes, visceral adipose tissue and short sleep suggested heterogeneities (Supplementary Figures 1–5). Although horizontal pleiotropy (*P* = 0.029, MR-Egger intercept test) was detected in the causal association between the smoking initiation and the risk of GORD, the result from MR-Egger method was still robust (*P* = 0.022, MR-Egger method).

### Meta-analysis for the discovery phase and replication phase

We performed UVMR for the 66 exposures and GORD_*meta*_, which contained 43116 GORD cases and 580251 controls from the GORD_*Neale*_ and GORD_*Finn*_, to complement and consolidate the causal associations between exposures and the risk of GORD. In total, as shown in Figure 4 and Supplementary Tables 6, 26 exposures were finally identified to be significantly causally associated with the risk of GORD (*P* ≤ 7.463E-04), which included 15 risk exposures: smoking initiation (OR = 1.284, *P* = 5.867E-06), lifetime smoking (OR = 1.971, *P* = 9.127E-14), cigarette consumption (OR = 1.211, *P* = 3.944E-05), insomnia (OR = 2.351, *P* = 1.957E-06), short sleep (OR = 3.450, *P* = 5.867E-06), leisure sedentary behavior (TV watching) (OR = 1.606, *P* = 1.835E-14), BMI (OR = 1.391, *P* = 1.859E-32), body fat percentage (OR = 1.494, *P* = 4.751E-22), whole body fat mass (OR = 1.297, *P* = 3.035E-19), visceral adipose tissue (OR = 1.314, *P* = 2.002E-13), waist circumference (OR = 1.402, *P* = 9.657E-18), hip circumference (OR = 1.151, *P* = 1.614E-06), waist-to-hip ratio (OR = 1.371, *P* = 1.978E-19), major depressive disorder (OR = 1.539, *P* = 1.084E-24), and anxious feeling (OR = 1.926, *P* = 3.214E-05); 11 protective exposures: leisure sedentary behavior (computer use) (OR = 0.721, *P* = 1.599E-04, FDR = 1.124E-04), height (standing and sitting) (OR = 0.900, *P* = 7.002E-07 and OR = 0.875, *P* = 1.739E-08), hand grip strength (left and right) (OR = 0.739, *P* = 9.596E-06 and OR = 0.771, *P* = 7.802E-05), birth weight (OR = 0.861, *P* = 4.522E-05), life satisfaction (OR = 0.412, *P* = 1.464E-11), positive affect (OR = 0.426, *P* = 6.869E-13), income (OR = 0.619, *P* = 5.427E-12), educational attainment (OR = 0.540, *P* = 1.121E-127), and intelligence (OR = 0.754, *P* = 2.252E-12). Meanwhile, smoking cessation, daytime sleepiness, daytime napping, adult-onset asthma, type 2 diabetes, coronary artery disease, ulcerative colitis suggestively causally increased risk of GORD, and strenuous sports or other exercises, HDL cholesterol, LDL cholesterol, leptin, and whole body fat-free mass suggestively causally decreased risk of GORD.

### Causalities independent of hub exposures

**FIGURE 5:**
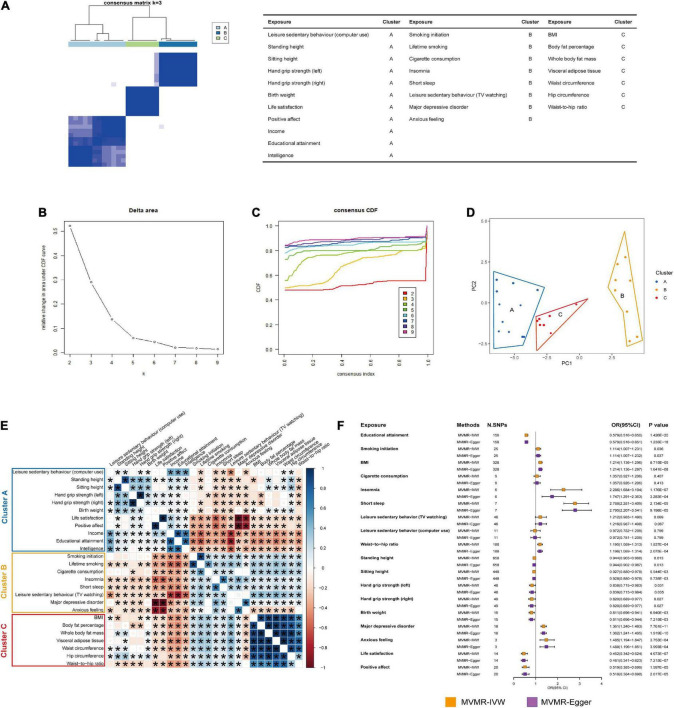
PAM clustering and MVMR analyses. **(A)** The PAM clustering matrix heatmap when *K* = 3. **(B)** The change of slope of CDF curve in PAM clustering for 26 exposures. **(C)** The change of area under CDF curve in PAM clustering for 26 exposures. **(D)** Principal component analysis (PCA) for 26 exposures in cluster A, cluster B, and cluster C. **(E)** Genetic correlations between 26 exposures. The size and color of squares respectively represented the significance level and genetic correlation directions. Genetic correlations with *P* ≤ 0.05 would be showed with asterisks. **(F)** The forest plot showed the results of the causal associations between 15 exposures and the risk of GORD after MVMR analyses with adjustment of educational attainment, smoking initiation, or BMI. The causal effects were represented as ORs with MVMR-IVW method (orange box) and MVMR-Egger method (purple box). The MVMR results estimated with MVMR-IVW method and MVMR-Egger method were summarized in Supplementary Table 7 in detail.

According to the results of the Bonferroni multiple comparisons from UVMR analyses, 26 causal exposures were identified to have significant causal effects on the risk of GORD. *K* = 3 (cluster A, cluster B, and cluster C) (Figures 5A–D) was used as the standard of the PAM algorithm to cluster 351 genetic correlations between 26 significant causal exposures (Figure 5E). Among them, educational attainment, smoking initiation and BMI in clusters A, B, and C were genetically correlated with all the other exposures individually, and were defined as hub exposures. MVMR analysis suggested that educational attainment (MVMR-IVW: OR = 0.579, *P* = 1.426E-20; MVMR-Egger: OR = 0.579, *P* = 1.235E-18), smoking initiation (MVMR-IVW: OR = 1.114, *P* = 0.036; MVMR-Egger: OR = 1.114, *P* = 0.037), and BMI (MVMR-IVW: OR = 1.214, *P* = 8.715E-09; MVMR-Egger: OR = 1.214, *P* = 1.641E-08) all showed independent causal effects on the risk of GORD. As the results of statistically significant genetic correlation of the other exposures with educational attainment, smoking initiation and BMI, we evaluated the independent causal effects of cigarette consumption, insomnia, short sleep, leisure sedentary behavior (TV watching), waist-to-hip ratio, height (standing and sitting), major depressive disorder, anxious feeling, life satisfaction and positive affect with adjustment of educational attainment (32), smoking initiation (33), and BMI (34), and independent causal effects of leisure sedentary behavior (computer use), hand grip strength (left and right) and birth weight with adjustment of educational attainment (32) and BMI (34). Exposures that respectively strongly genetically correlated with the BMI [body fat percentage (*r*_*g*_ = 0.902, *P* = 0.000), whole body fat mass (*r*_*g*_ = 0.912, *P* = 0.000), visceral adipose tissue (*r*_*g*_ = 0.932, *P* = 0.000), waist circumference (*r*_*g*_ = 0.900, *P* = 0.000), and hip circumference (*r*_*g*_ = 0.849, *P* = 0.000)], smoking initiation [lifetime smoking (*r*_*g*_ = 0.921, *P* = 0.000)], and educational attainment [income (*r*_*g*_ = 0.804, *P* = 0.000) and intelligence (*r*_*g*_ = 0.807, *P* = 0.000)] were excluded from MVMR analyses. After MVMR analyses (Figure 5F; Supplementary Table 7), it was found that insomnia (MVMR-IVW: OR = 2.286, *P* = 1.176E-07; MVMR-Egger: OR = 1.747, *P* = 3.283E-04), short sleep (MVMR-IVW: OR = 2.788, *P* = 2.134E-05; MVMR-Egger: OR = 2.795, *P* = 9.198E-05), waist-to-hip ratio (MVMR-IVW: OR = 1.195, *P* = 1.837E-04; MVMR-Egger: OR = 1.196, *P* = 2.075E-04), height (standing and sitting) (standing: MVMR-IVW: OR = 0.944, *P* = 0.013; MVMR-Egger: OR = 0.944, *P* = 0.013; sitting: MVMR-IVW: OR = 0.928, *P* = 5.544E-03; MVMR-Egger: OR = 0.928, *P* = 5.735E-03), hand grip strength (left and right) (left: MVMR-IVW: OR = 0.838, *P* = 0.031; MVMR-Egger: OR = 0.839, *P* = 0.035; right: MVMR-IVW: OR = 0.820, *P* = 0.027; MVMR-Egger: OR = 0.820, *P* = 0.827), birth weight (MVMR-IVW: OR = 0.811, *P* = 6.940E-03; MVMR-Egger: OR = 0.811, *P* = 7.215E-03), major depressive disorder (MVMR-IVW: OR = 1.361, *P* = 7.761E-11; MVMR-Egger: OR = 1.362, *P* = 1.919E-10), anxious feeling (MVMR-IVW: OR = 1.485, *P* = 3.753E-04; MVMR-Egger: OR = 1.485, *P* = 3.993E-04), life satisfaction (MVMR-IVW: OR = 0.462, *P* = 4.673E-07; MVMR-Egger: OR = 0.461, *P* = 7.215E-07), and positive affect (MVMR-IVW: OR = 0.519, *P* = 1.597E-05; MVMR-Egger: OR = 0.518, *P* = 2.017E-05) retained their causal effects on the risk of GORD, similar to the UVMR analyses. Moreover, cigarette consumption and leisure sedentary behaviors (TV watching and computer use) did not exhibit independent causal associations with the risk of GORD following MVMR analyses.

## Discussion

Previous MR studies examined the causal associations between smoking initiation, asthma, type 2 diabetes, BMI, waist circumference, waist-to-hip ratio, standing height, and the risk of GORD primarily among European-ancestry individuals in the UKB database (16, 35–37), whose MR findings were also replicated in this study with larger-scale GORD GWAS datasets from Neale Lab and FinnGen consortium. In this study, we systematically examined causal associations between 66 exposures across 6 modifiable pathways and the risk of GORD in order to comprehensively elucidate the causal modifiable factors that were associated with the risk of GORD. Totally, 26 significant causal associations and 12 suggestive causal associations were observed. Meanwhile, after MVMR analyses, 15 significant causal exposures among smoking behaviors, sleep disorders, obesity, muscle mass, height (standing and sitting), negative emotions, positive subjective well-beings, and socioeconomic factors retained independent causal associations with the risk of GORD.

### Novel significant risk causal associations

Obesity, especially central obesity, contributes the risk of GORD by increasing the frequency of the transient lower oesophageal sphincter relaxation (TLESR) (38), intra-abdominal pressure (39) and the possibility of oesophageal acid exposure (40). We further extended MR analyses to include body fat percentage, whole body fat mass, visceral adipose tissue, waist circumference, hip circumference, and waist-to-hip ratio to systematically demonstrate causal associations between central obesity and the risk of GORD. A higher waist-to-hip ratio is observed to be a better indicator of the occurrence of GORD than BMI (41), and is also associated with the development of GORD (42). In this study, it was further confirmed that the waist-to-hip ratio, whether it was adjusted for BMI or not, presented a strong causal association with the risk of GORD. At present, there are few epidemiological investigations on the association between height and the pathogenesis of GORD. We speculated that taller people have less abdominal pressure and were therefore less likely to develop GORD. The BMI-adjusted and BMI-unadjusted MVMR analyses also verified the independent reverse causalities of the height (standing and the sitting) with the risk of GORD.

Several large cohort studies and meta-analyses have demonstrated evidence linking smoking to GORD (3, 43, 44). Currently, it is believed that smoking causes GORD by prolonging the acid gap time, decreasing the secretion of saliva and neutralizing bases (45) and loosening the lower oesophageal sphincter (LES) (46). In this study, the causalities between smoking behaviors and the risk of GORD were explored systematically from smoking initiation, lifetime smoking, cigarette consumption, and smoking cessation, and the independent positive causal associations between smoking initiation and the risk of GORD were also demonstrated.

Poor sleep quality appeared to be closely related to the risk of GORD. Mody et al. recorded 68.3% of 11685 GORD respondents with sleep difficulties, 49.1% experiencing difficulty falling sleep, and 58.3% struggling to stay asleep (47). This study provided genetic evidence that insomnia and short sleep contributed to the risk of GORD. At present, the associations between insomnia, short sleep and BMI as well as smoking initiation have been demonstrated (48, 49). We performed MVMR analyses with adjustment of educational attainment, BMI and smoking initiation for insomnia and short sleep and confirmed the independence of the causal association between insomnia, short sleep, and the risk of GORD.

As of now, epidemiological studies are lacking regarding the direct impact of sedentary behavior on the risk of GORD. In a UK prospective cohort study, sedentary occupations had a higher risk of Barrett’s oesophagitis comparing with standing occupations (50). In this study, a significant positive causality was found between prolonged TV watching time and the risk of GORD; in contrast, prolonged computer use time had a significant negative causality with the risk of GORD. Furthermore, we also attempted to assess whether sedentary behavior had an independent causal effect on the risk of GORD to rule out the potential causal effects of the remaining factors, such as BMI, smoking behaviors, and educational attainment (51), on the risk of GORD. Following MVMR analyses for TV watching and computer use, we detected that the strong causal effects were attenuated in comparison with the original, which exactly evidenced that sedentary behaviors including TV watching and computer use did not have causal effects on the risk of GORD independently.

Gastro-oesophageal reflux disease is common in individuals with anxious feeling and depression. A recent large cross-sectional study based on 19,099 participants provided evidence that anxious feeling and depression were associated with an increased risk of GORD (52). Corticotropin-releasing hormone (CRH) is a key mediating factor for emotional stress. Broers et al. illustrated that CRH increased oesophageal sensitivity along with increased oesophageal contractility and decreased relaxation of LES, thus causing reflux symptoms (53). In the investigation of GORD pathogenesis, anxious feeling and depression might also be implicated in a similar pattern. GORD may be exacerbated by anxious feeling and depression by reducing the visceral sensitivity index (VSI), enhancing reflux hypersensitivity, and functional heartburn (54). Our study further highlighted the independent causalities of anxious feeling and depression on the risk of GORD.

### Novel significant protective causal associations

Hand grip strength are customarily considered as surrogates for skeletal muscle mass, which is typically influenced by malnutrition and aging (55, 56). In this study, we found that hand grip strength (left and right) had significant negative causal associations with the risk of GORD regardless of BMI and educational attainment. The relationship between muscle mass and the risk of GORD was preliminarily investigated in a context of previous cohort study. A cohort study based on 574 patients with sarcopenia indicated that sarcopenia was positively associated with a high risk of GORD (OR = 1.170, 95% CI = 1.016–1.346) (57). Although the underlying mechanisms of the relationship between muscle mass and the risk of GORD are still obscure, this study suggested that there was a deterministic reverse causal association between muscle mass and the risk of GORD at the genetic level, which might serve as a catalyst for further mechanistic studies. Currently, adults with low birth weight are closely bound up with the risk of GORD (58). Current researches support the hypothesis that GORD is a chronic disease that will last throughout the whole life. It usually begins in infancy, but does not progress in childhood, while becomes clinically significant in adulthood (59, 60). In this study, we confirmed an independently inverse causal association between birth weight based on a GWAS dataset from a long-term population study and the risk of GORD.

It has been demonstrated that specific negative emotions have causal effects on the increased risk of GORD; however, there have been rare studies to investigate the correlations between positive emotions and the risk of GORD. An enlightening finding revealed that effective psychological intervention therapy has been shown to significantly improve GORD symptoms, such as globus sensation and non-cardiac chest pain (61), or, to put it another way, there was a theoretical possibility that protective effects of positive emotions on GORD could be achieved. Our study replenished these protective causalities by confirming that the positive subjective feelings (such as positive affect and life satisfaction) were significantly causally associated with a decreased risk of GORD. It could also be interpreted that positive subjective feelings contributed to protection against the risk of GORD.

Socioeconomic factors are also strongly associated with the risk of GORD in many aspects as well. According to a global meta-analysis containing 102 studies (3), the low-income groups had a higher prevalence of GORD compared with the middle-income groups (OR: 1.58, 95% CI: 1.20–2.08) or the high-income groups (OR: 1.68, 95% CI: 1.38–2.05); those with lower educational levels had a higher prevalence of GORD than the individuals at medium educational levels (OR: 1.47, 95% CI: 1.25–1.73) and the individuals with higher educational levels (OR: 1.78, 95% CI: 1.39–2.28). In this study, reverse causal associations between income, educational attainment as well as intelligence and the risk of GORD were also established. Typically, a higher educational level correlates with healthier lifestyles, better economic status, healthier cognition and more consummate medical care (62, 63). Socioeconomic factors, including income, educational attainment and intelligence, all played protective roles in the risk of GORD, while focusing on a single social factor and discrediting it as confounding was also unreasonable for the risk of GORD. Perhaps the causal associations of the socioeconomic factors on the risk of GORD can also be regarded as the consequence of a wide-ranging interaction.

### Strengths and limitations

We reviewed and summarized the modifiable factors and previous MR results that were associated with the risk of GORD to expand the scope of exposures included in the MR analysis to the largest extent, ensuring comprehensiveness of MR analysis. This study was also the first to rigorously and systematically examine genetic liabilities of modifiable factors for the risk of GORD, mitigating the impacts of potential confounders and reverse causalities as much as possible. Moreover, MR results in the discovery phase and replication phase were complementary, and meta-analysis for the above two phases enhanced the causal associations between exposures and the risk of GORD. MVMR analyses adjusted for hub exposures (educational attainment, smoking initiation, or BMI) estimated from PAM clustering algorithm also revealed novel insights into the independent causal effects of specific exposures on the risk of GORD.

Some limitations were also taken into consideration in this study. Firstly, despite the fact that we systematically searched and summarized the factors related to the risk of GORD and SNPs recruited from exposures, the limitations of review scope design, as well as the lack of SNPs for some exposures might result in omissions or deviations when assessing causal associations. Secondly, it is generally accepted in MR analysis that there are two mechanisms for pleiotropy: one is that a genetic variant directly affects multiple phenotypes, which is defined as type I pleiotropy (horizontal pleiotropy), and the second is that a genetic variant affects one exposure, while simultaneously also affecting other phenotypes through this expose, which is defined as type II pleiotropy (vertical pleiotropy) (64). As a general rule, type II pleiotropy (vertical pleiotropy) has no biased effects on causality. Therefore, we supplemented the MR-Egger method and MR-PRESSO methods in the MR analysis in order to minimize the bias with the detection of type I pleiotropy (horizontal pleiotropy) as much as possible. Thirdly, given that this study was mainly based on the European population, extrapolating the causal associations in this study to other ethnic populations may result in deviations due to genetic heterogeneities among different ethnic populations.

## Conclusion

In summary, we performed MR analysis in this study to identify the factors causally associated with the risk of GORD from 66 exposures across 6 modifiable pathways, which also provided references on the public preventive and therapeutic strategies for GORD from multiple perspectives, such as weight loss, proper high-level physical activities, high-quality sleep, non-smoking, and positive mental state.

## Data availability statement

The original contributions presented in this study are included in the article/Supplementary material, further inquiries can be directed to the corresponding author.

## Ethics statement

Consents for GWAS datasets were all acquired from public database.

## Author contributions

YS and JJ designed this study. YS, XC, and KS drafted the manuscript. YS, XC, DC, YC, KS, ZJ, and JJ conducted data collection and analysis. YW and JJ provided medical ethical reference. All authors contributed to the article and approved the submitted version.

## Footnotes

1
http://www.nealelab.is/uk-biobank

2
https://r6.finngen.fi/

3
https://pan.ukbb.broadinstitute.org/phenotypes

4
https://hail.is/docs/batch/index.html

5
https://github.com/weizhouUMICH/SAIGE/tree/finngen_r6_jk

6
https://alkesgroup.broadinstitute.org/LDSCORE

7
https://shiny.cnsgenomics.com/mRnd/
