# Supplementary material for: Genetic estimation of correlations and causalities between multifaceted modifiable factors and gastro-oesophageal reflux disease
Source: Front Nutr. 2022 Nov 1;9:1009122. doi: 10.3389/fnut.2022.1009122 (PMC9663808; doi:10.3389/fnut.2022.1009122)
Supplement: Supplementary file 1 [file Data_Sheet_1.PDF]

# Genetic estimations of correlations and causalities between multifaceted modifiable factors and gastro-oesophageal reflux disease

Yuanlin Sun[1], Xueyuan Cao[1], Donghui Cao[2], Yingnan Cui[1], Kaisheng Su[2], Zhifang Jia[2], Yanhua Wu[2], Jing Jiang[2*]

**Contents of supplementary file**

**Table S1.** An overview of the modifiable factors associated with the risk of GORD
**Table S2.** The detailed information of the 66 exposures included in MR analysis
**Table S3.** The summary of statistical genetic correlation (P≤0.05)
**Table S4.** The summary of the MR results in the discovery phase
**Table S5.** The summary of the MR results in the replication phase
**Table S6.** The summary of the MR results of the meta-analysis
**Table S7.** The summary of the MVMR results with MVMR-IVW and MVMR-Egger method
**Figure S1.** Scatter plots and funnel plots for exposures (smoking initiation, lifetime smoking, cigarette consumption, smoking cessation, insomnia, short sleep, daytime sleepiness and daytime napping) for $GORD_{Neale}$, $GORD_{Finn}$ and $GORD_{Meta}$
**Figure S2.** Scatter plots and funnel plots for exposures (leisure sedentary behavior (TV watching), Leisure sedentary behavior (computer use), 10+ minutes vigorous activity, strenuous sports or other exercises, adult-onset asthma, type 2 diabetes, coronary artery disease and ulcerative colitis) for $GORD_{Neale}$, $GORD_{Finn}$ and $GORD_{Meta}$
**Figure S3.** Scatter plots and funnel plots for exposures (HDL cholesterol, LDL cholesterol, leptin, BMI, body fat percentage, whole body fat mass, whole body fat-free mass and visceral adipose tissue) for $GORD_{Neale}$, $GORD_{Finn}$ and $GORD_{Meta}$
**Figure S4.** Scatter plots and funnel plots for exposures (waist circumference, hip circumference, waist-to-hip ratio, height (standing and sitting), hand grip strength (left and right) and birth weight) for $GORD_{Neale}$, $GORD_{Finn}$ and $GORD_{Meta}$
**Figure S5.** Scatter plots and funnel plots for exposures (major depressive disorder, anxious feeling, schizophrenia, life satisfaction, positive affect, income, educational attainment and intelligence) for $GORD_{Neale}$, $GORD_{Finn}$ and $GORD_{Meta}$

## Table S1. An overview of the modifiable factors associated with the risk of GORD

| Factors | Previous MR analysis conducted (Yes/No) | Previous MR analysis conducted (PMID) | Previous MR analysis conducted (Published reference) |
|---|---|---|---|
| **Daily habits** | | | |
| Alcohol consumption | Yes | PMID: 35119566 | Adiposity, diabetes, lifestyle factors and risk of gastroesophageal reflux disease: a Mendelian randomization study |
| Coffee consumption | Yes | PMID: 35119566 | Adiposity, diabetes, lifestyle factors and risk of gastroesophageal reflux disease: a Mendelian randomization study |
| Caffeine consumption | No | PMID: 35119566 | Adiposity, diabetes, lifestyle factors and risk of gastroesophageal reflux disease: a Mendelian randomization study |
| Smoking behaviors | Yes | PMID: 35119566 | Adiposity, diabetes, lifestyle factors and risk of gastroesophageal reflux disease: a Mendelian randomization study |
| Sugar intakes | No | NA | NA |
| Fat intakes | No | NA | NA |
| Carbohydrate intakes | No | NA | NA |
| Breakfast skipping | No | NA | NA |
| Circadian rhythm | No | NA | NA |
| Sleep disorders | No | NA | NA |
| Daytime sleepiness | No | NA | NA |
| Daytime napping | No | NA | NA |
| Physical activity levels | No | NA | NA |
| **Health status** | | | |
| Childhood-onset asthma | Yes | PMID: 35292014 | Asthma and the risk of gastrointestinal disorders: a Mendelian randomization study |
| Adult-onset asthma | Yes | PMID: 35292014 | Asthma and the risk of gastrointestinal disorders: a Mendelian randomization study |
| Type 2 diabetes | Yes | PMID: 35119566 | Adiposity, diabetes, lifestyle factors and risk of gastroesophageal reflux disease: a Mendelian randomization study |
| Coronary artery disease | No | NA | NA |
| Atrial fibrillation | No | NA | NA |
| Ulcerative colitis | No | NA | NA |
| Crohn's disease | No | NA | NA |
| **Nutritional & biochemical biomarkers** | | | |
| Fasting glucose | No | NA | NA |
| Insulin | No | NA | NA |
| Postprandial plasma glucose | No | NA | NA |
| Glycosylated hemoglobin (HbA1c) | No | NA | NA |
| HDL cholesterol | No | NA | NA |
| LDL cholesterol | No | NA | NA |
| Total cholesterol | No | NA | NA |
| Triglycerides | No | NA | NA |
| Lipoprotein | No | NA | NA |
| Adiponectin | No | NA | NA |
| Leptin | No | NA | NA |
| Vitamin C | No | NA | NA |
| Vitamin D | No | NA | NA |
| **Nutritional & developmental status** | | | |
| Obesity | Yes | PMID: 35119566 | Adiposity, diabetes, lifestyle factors and risk of gastroesophageal reflux disease: a Mendelian randomization study |
| Visceral adipose tissue | Yes | No | NA |
| Waist circumference | Yes | PMID: 32588049 | Genetic evidence that higher central adiposity causes gastro-oesophageal reflux disease: a Mendelian randomization study |
| Hip circumference | Yes | PMID: 32588049 | Genetic evidence that higher central adiposity causes gastro-oesophageal reflux disease: a Mendelian randomization study |
| Waist-to-hip ratio | Yes | PMID: 32588049 | Genetic evidence that higher central adiposity causes gastro-oesophageal reflux disease: a Mendelian randomization study |
| Weight loss | No | NA | NA |
| Height | Yes | PMID: 30355295 | Adult height and risk of 50 diseases: a combined epidemiological and genetic analysis |
| Muscle mass | No | NA | NA |
| Birth weight | No | NA | NA |
| Childhood obesity | No | NA | NA |
| **Emotional factors** | | | |
| Negative emotions | No | NA | NA |
| Positive subjective well-beings | No | NA | NA |
| **Socioeconomic factors** | | | |
| Income | No | NA | NA |
| Educational attainment | No | NA | NA |
| **Sex-hormone-related factors** | | | |
| Menopausal transit | No | NA | NA |
| Oestradiol levels (female) | No | NA | NA |
| Oestradiol levels (male) | No | NA | NA |
| Sex hormone-binding globulin levels (female) | No | NA | NA |
| Sex hormone-binding globulin levels (male) | No | NA | NA |
| Testosterone levels (female) | No | NA | NA |
| Testosterone levels (male) | No | NA | NA |
| **Common medications** | | | |

| | | | |
|---|---|---|---|
| Aspirin | No | NA | NA |
| Calcium channel blockers | No | NA | NA |
| Statin | No | NA | NA |
| Metformin | No | NA | NA |

**Table S2. The detailed information of the 66 exposures included in MR analysis.**

| Exposure | Source | Definition | Unit | GWAS model | Covariates |
|---|---|---|---|---|---|
| **Daily habits** | | | | | |
| Alcohol consumption | PMID: 30643251 | The average number of drinks a participant reported drinking each week, aggregated across all types of alcohol. | Drinks per week | BOLT-LMM | Age, age-squared, sex, and genetic principal components |
| Coffee consumption | PMID: 31046077 | 24-h recall questionnaire about cups of coffee drinked each day | Cups per day | BOLT-LMM | Age, sex, top 20 principal components of population sub-structure. |
| Caffeine consumption | PMID: 33287642 | Caffeine intake was calculated as the number of cups of coffee or tea multiplied by the caffeine content in per cup of different types of coffee or tea. Combined caffeine intake from both coffee and tea was calculated as the sum of the daily caffeine intake from coffee and tea from individuals. | mg per day | BOLT-LMM | Age, sex, genotyping array, and the first 30 principal components to adjust for population stratification |
| Smoking initiation (current or former vs. never) | PMID: 30643251 | Binary phenotype with current smokers or former smokers and never being regular smokers | Events | BOLT-LMM | Age, age-squared, sex and genetic principal components |
| Lifetime smoking | PMID: 31689377 | Smoking measures available in the UK Biobank were self-reported and collected at initial assessment. They included: smoking status (current, former, never – field 20116), age at initiation in years (fields 3436/2867), age at cessation in years (field 2897) and number of cigarettes smoked per day (fields 3456/2887). Anyone self-reporting to smoke more than 100 cigarettes per day was contacted for confirmation. Hand-rolled cigarette smokers were told 1 g of tobacco equates to one cigarette. UK Biobank removed individuals smoking fewer than 1 or more than 150 cigarettes a day. Based on the method outlined by Leffondré, Abrahamowicz, Xiao, and Siemiatycki, smoking measures were combined into a lifetime smoking index along with a simulated half-life (τ) constant. | Lifetime smoking index | BOLT-LMM | Sex and genotyping chip |
| Cigarette consumption | PMID: 30643251 | The average number of cigarettes smoked per day, either as a current smoker or former smoker, and whether self-rolled or manufactured are smoked. | Cigarettes per day | BOLT-LMM | Age, age-squared, sex, and genetic principal components. |
| Age of initiation of smoking | PMID: 30643251 | Age at which an individual started smoking cigarettes regularly | Years | | |
| Smoking cessation (current vs. former smokers) | PMID: 30643251 | Binary phenotype with current smokers and former smoker | Events | | |
| Intake of total sugar | PMID: 32393786 | Dietary intakes derived from 24h-dietary-recall questionnaires exhibiting 200 food items. For convenience, the energy values of the macronutrients were obtained using the conversion factor of 4 kcal/gram for sugar and carbohydrate, and 9 kcal/gram for fat. | Kcal/day | BOLT-LMM | Sex, age, age-squared, sex multiply by age, sex multiply by age- squared, and the first 10 principal components of the cohort- specific genotype matrix |
| Intake of fat | PMID: 32393786 | | Kcal/day | | |
| Intake of carbohydrate | PMID: 32393786 | | Kcal/day | | |
| Breakfast skipping | PMID: 31190057 | 24h-diet-recall questionnaires were used to estimate the participants breakfast skipping. Responses from all completed recalls (≤5) were considered for each participant. Responses were categorized as “breakfast skipping” if the participant always responded “No”, “sometimes breakfast skipping” if the participant sometimes responded “Yes”, and “breakfast consumers” if the participant always responded “Yes”. | Always/sometimes/never | BOLT-LMM | Age, sex, 10 principal components of ancestry |
| Morning person | PMID: 30696823 | A binary phenotype using the same data-field as for Chronotype. Participants answering “Definitely an ‘evening’ person” and “More an ‘evening’ than a ‘morning’ person” were coded as 0 (controls) and those answering “Definitely a ‘morning’ person” and “More a ‘morning’ than ‘evening’ person” were coded as 1 (cases). Participants answering “Do not know” or “Prefer not to answer” were coded as missing. | Events | BOLT-LMM | Age, sex, study centre and a derived variable representing genotyping release |
| Insomnia (never/rarely vs. usually) | PMID: 30804566 | A binary phenotype about the self-reported insomnia symptom being permeated into the question “do you have trouble falling asleep at night or do you wake up in the middle of the night?” was recorded as “never/rarely” vs. “usually”. | Events | BOLT-LMM | Age, sex, 10 principal components and genotyping array |
| Short sleep (<7h vs. 7-8h/d) | PMID: 30846698 | Participants were asked: About how many hours sleep do you get in every 24 h? (including naps), with responses in hour increments. Sleep duration was treated as a continuous variable and also categorized as either short (6 h or less), normal (7 or 8 h), or long (9 h or more) sleep duration. Extreme responses of less than 3 h or more than 18 h were excluded and do not know or prefer not to answer responses were set to missing. Participants who self-reported any sleep medication were excluded. | Events | BOLT-LMM | Age, sex, 10 principal components of ancestry, genotyping array, and genetic correlation matrix |
| Long sleep (⩾9h vs. 7-8h/d) | PMID: 30846698 | | Events | | |
| Daytime sleepiness | PMID: 31409809 | Self-reported daytime sleepiness was ascertained using the question “How likely are you to dose off or fall asleep during the daytime when you don’t mean to? (e.g. when working, reading or driving)” with the response options of “Never/rarely”, “sometimes”, “often”, “all of the time”, “do not know”, and “prefer not to answer”. Participants reporting “do not know” and “prefer not to answer” were set to missing. Other responses were coded continuously as 1 to 4 corresponding to the severity of daytime sleepiness. | Never/ sometimes/often/always | BOLT-LMM | Age, sex, genotyping array,10 principal components of ancestry and genetic relatedness matrix |
| Daytime napping | PMID: 33568662 | Participants were asked "Do you have a nap during the day?" with responses Never/rarely, Sometimes, Usually, prefer not to answer. Responses were treated as continuous variable in the GWAS. Prefer not to answer responses were set to missing. | Never or rarely/sometimes/always | BOLT-LMM | Age, sex, 10 principal components of ancestry, genotyping array and genetic correlation matrix with a maximum per SNP missingness of 10% and per sample missingness of 40% |

| | | | | | |
|---|---|---|---|---|---|
| Leisure sedentary behavior (TV watching) | PMID: 32317632 | During the first visit, participants were asked three questions, “In a typical day, how many hours do you spend watching TV?”, “In a typical day, how many hours do you spend using the computer? (Do not include using a computer at work)” and “In a typical day, how many hours do you spend driving?”. Participants outside a 99.5% range on the right side of the normal distribution were excluded on a per-phenotype basis. | Hours | BOLT-LMM | age-squared, age, sex, age-sex interaction, the first 30 principal components and genotyping array |
| Leisure sedentary behavior (computer use) | PMID: 32317632 | | Hours | | |
| Moderate to vigorous physical activity levels | PMID: 29899525 | Moderate-to-vigorous physical activity was calculated by taking the sum of total minutes/week of moderate physical activity multiplied by four and the total number of vigorous physical activity minutes/week multiplied by eight, corresponding to their metabolic equivalents. | MET-minutes/week | BOLT-LMM | Age, sex, genotyping chip, first 10 genomic principal components, and center as independent variables |
| 10+ minutes vigorous activity (⩾3 vs 0 days/week) | PMID: 29899525 | Participants were asked: “In a typical week, how many days did you do 10 minutes or more of vigorous physical activity? (These are activities that make you sweat or breathe hard such as fast cycling, aerobics, heavy lifting)”. Individuals who selected “prefer not to answer” or “do not know” on the above questions, those reporting not being able to walk, and individuals reporting more than 16 hours vigorous physical activity per day were excluded. | Events | | |
| Strenuous sports or other exercises (⩾2-3 vs. 0 days/week) | PMID: 29899525 | Participants were asked “In the last 4 weeks did you spend any time doing the following?” to assess the frequency and typical duration of “strenuous sports” and of “other exercises”. The possible responses to the initial question were: ‘walking for pleasure’, ‘other exercises’, ‘strenuous sports’, ‘light DIY’, ‘heavy DIY’, ‘none of the above’, and ‘prefer not to answer’. Individuals spending 2-3 days/week or more doing strenuous sports or other exercises were identified, for a duration of 15- 30 minutes or greater. Controls were those individuals who did not indicate spending any time in the last 4 weeks doing either strenuous sports or other exercises. Individuals that did not fall into either of these two groups were excluded. | Events | | |
| **Health status** | | | | | |
| Childhood-onset asthma | PMID: 30929738 | Childhood-onset asthma: a child or teenager, specifically at or before age 19 (n = 13,962) was first diagnosed as asthma; Adult-onset asthma: a young or middle- aged adult, between the ages of 20 and 39 (n = 11,709); or an older adult, between the ages of 40 and 60 (n = 14,873), was first diagnosed as asthma. | Events | BOLT-LMM | Age, sex, and an indicator of the genotyping array |
| Adult-onset asthma | PMID: 30929738 | | Events | | |
| Type 2 diabetes | PMID: 30297969 | Summary-level data for type 2 diabetes were retrieved from the DIAbetes Genetics Replication And Meta-analysis (DIAGRAM) consortium, which contains 32 studies for 898,130 European individuals (74124 cases and 824006 controls). | Events | Linear mixed model | Age, sex, and the first 10 genetic principal components |
| Coronary artery disease | PMID: 29212778 | CAD was defined using the following ICD 10 codes: I21-I25 covering ischemic heart diseases and the following Office of Population Censuses and Surveys Classification of Interventions and Procedures, version 4 (OPCS-4) codes: K40-K46, K49, K50 and K75 which includes replacement, transluminal balloon angioplasty, and other therapeutic transluminal operations on coronary artery and percutaneous transluminal balloon angioplasty and insertion of stent into coronary artery. Self-reported CAD was also used in the definition (heart attack/myocardial infarction, coronary angioplasty +/- stent and triple heart bypass). | Events | BOLT-LMM | Age, gender, the first 30 principal components to account for population stratification and genotyping array |
| Atrial fibrillation | PMID: 30061737 | Summary-level data from the selected cohorts including HUNT, deCODE, MGI, DiscovEHR, UK Biobank and AFGen Consortium. | Events | BOLT-LMM | Age, sex and the first 4 principal components |
| Ulcerative colitis | PMID: 26192919 | Recruitment of patients and matched controls genotyped with the Immunochip was performed in 15 countries in Europe, North America and Oceania. Diagnosis of IBD was based on accepted radiologic, endoscopic, and histopathologic evaluation. All included cases fulfill clinical criteria for IBD. | Events | Linear mixed model | The 7 principal components |
| Crohn's disease | PMID: 26192919 | | Events | | The 10 principal components |
| **Nutritional & biochemical biomarkers** | | | | | |
| Fasting glucose | PMID: 34059833 | Analyses included data for FG and 2hGlu measured in mmol/l, FI measured in pmol/l, and HbA1c in %. Individuals were excluded if they had type 1 or type 2 diabetes (defined by physician diagnosis); reported use of diabetes-relevant medication(s); or had a FG ≥7 mmol/L, 2hGlu ≥11.1mmol/L, or HbA1c ≥ 6.5%. 2h Glu measures were obtained 120 minutes after a glucose challenge in an oral glucose tolerance test (OGTT). Measures for FG and FI taken from whole blood were corrected to plasma level using the correction factor 1.1380. | mmol/L | Linear model | study-specific covariates, and principal components |
| Fating insulin | PMID: 34059833 | | pmol/L | | |
| 2-hour blood glucose | PMID: 34059833 | | mmol/L | | |
| Glycosylated hemoglobin (HbA1c) | PMID: 34059833 | | Percentage | | |
| HDL cholesterol | PMID: 24097068 | Blood lipid levels were typically measured after >8 h of fasting. Individuals known to be on lipid-lowering medication were excluded. LDL cholesterol levels were directly measured in ten studies (24% of total study individuals) and were estimated using the Friedewald formula. | mg/dL | BOLT-LMM and SAIGE | Age, age-squared, principal components of ancestry and any necessary study-specific covariates |
| LDL cholesterol | PMID: 24097068 | | mg/dL | | |
| Total cholesterol | PMID: 24097068 | | mg/dL | | |
| Triglycerides | PMID: 24097068 | | mg/dL | | |
| Adiponectin | PMID: 22479202 | Adiponectin levels were measured using ELISA or RIA methods | ln(mg/dL) | Addictive model | Age, sex, BMI, principal components of population stratification, study sites and for family structure in cohorts |
| Leptin | PMID: 26833098 | Circulating leptin levels in serum | log(ng/mL) | Linear regression model | Age, age-squared and any necessary study-specific covariates |

| | | | | | |
|---|---|---|---|---|---|
| Vitamin C | PMID: 33203707 | Plasma vitamin C was measured using high-performance liquid chromatography with ultraviolet detection in EPIC-InterAct and EPIC-CVD using samples that had been stored at −196°C (−150°C for Danish samples). In EPIC-Norfolk and Fenland, plasma vitamin C was measured with a fluorometric assay using samples stored at −70°C. For each of these studies, plasma was stabilized in a standardized volume of metaphosphoric acid. The cohorts included the meta-analysis of GWAS of plasma vitamin C levels were the Fenland study (n = 10,771), European Prospective Investigation into Cancer and Nutrition (EPIC)-InterAct study (n== 16,841), EPIC Norfolk study (n = 16,756, excluding duplicated samples with EPIC-InterAct), and the EPIC-CVD study (n = 7,650, excluding duplicated samples with EPIC-InterAct or EPIC-Norfolk). | μmol/L | Linear regression model | Age, sex, study center and the first 10 genetic principal components of ancestry |
| 25-Hydroxyvitamin D | PMID: 32242144 | Vitamin D 25OHD levels were measured in blood samples collected at two instances: the initial assessment visit, conducted between 2006 and 2010, and a repeat assessment visit, conducted between 2012 and 2013. The Diasorin Liason®, a chemiluminescent immunoassay (CLIA) was used for the quantitative determination of 25OHD. The assay measures total 25OHD concentration (i.e., 25OHD3 and 25OHD2). Participants with 25OHD concentrations below or above the validated range for the assay (10–375 nmol L−1) were excluded. The average within-laboratory coefficient of variation (CV) (and standard deviation) ranged from 5.04 (4.73) to 6.14 (2.21). | nmol/L | Linear mixed model | Age at time of assessment, sex, assessment month, assessment centre, supplement-intake information, genotyping batch and the first 40 ancestry principal components |
| **Nutritional & developmental status** | | | | | |
| Body mass index | MRC-IEU | Dataset: ukb-b-19953; Category: continuous; 21001: Output from GWAS pipeline using Phesant derived variables from UKBiobank | Kg/m^2 | BOLT-LMM | Sex and genotype array |
| Body fat percentage | MRC-IEU | Dataset: ukb-b-8909; Category: continuous; 23099: Output from GWAS pipeline using Phesant derived variables from UKBiobank | Percentage | | |
| Whole body fat mass | MRC-IEU | Dataset: ukb-b-19393; Category: continuous; 23100: Output from GWAS pipeline using Phesant derived variables from UKBiobank | Kg | | |
| Whole body fat-free mass | MRC-IEU | Dataset: ukb-b-13354; Category: continuous; 23101: Output from GWAS pipeline using Phesant derived variables from UKBiobank | Kg | | |
| Visceral adipose tissue | PMID: 31501611 | Initially, two UKBB subcohorts were constructed: one training dataset with VAT mass measured by DXA (instance 2; n=5109), to which the prediction models were calibrated; and one application dataset (instances 0 and 1; n=502638), in which VAT mass was predicted using the calibrated prediction models. To avoid potential bias due to population structure, only white British participants were included in the analysis, and all entries of "do not know", "prefer not to answer" or "NA" for any of the explored predictor variables or in the response were set to missing. After removal, n=4212 participants remained in the training dataset and n=397,170 participants remained in the application dataset. | Kg | BOLT-LMM | Age, a batch indicator variable for the two genotyping arrays and the first 10 principal components |
| Waist circumference | MRC-IEU | Dataset: ukb-b-9405; Category: continuous; 48: Output from GWAS pipeline using Phesant derived variables from UKBiobank | cm | BOLT-LMM | Sex and genotype array |
| Hip circumference | MRC-IEU | Dataset: ukb-b-15590; Category: continuous; 49: Output from GWAS pipeline using Phesant derived variables from UKBiobank | cm | | |
| Waist-to-hip ratio | PMID: 30239722 | The waist-to-hip ratio is the dimensionless ratio of the circumference of the waist to that of the hip. In this study, previous GWAS dataset of the GIANT consortium studies with new GWAS dataset of the waist-to-hip ratio in participants of the UK Biobank. | Percentage | BOLT-LMM | Sex, age, age-squared and assessment centre |
| Standing height | MRC-IEU | Dataset: ukb-b-10787; Category: continuous; 50: Output from GWAS pipeline using Phesant derived variables from UKBiobank | cm | BOLT-LMM | Sex and genotype array |
| Sitting height | MRC-IEU | Dataset: ukb-b-16881; Category: continuous; 20015: Output from GWAS pipeline using Phesant derived variables from UKBiobank | cm | | |
| Hand grip strength (left) | MRC-IEU | Dataset: ukb-b-7478; Category: continuous; 46: Output from GWAS pipeline using Phesant derived variables from UKBiobank. | Kg | | |
| Hand grip strength (right) | MRC-IEU | Dataset: ukb-b-10215; Category: continuous; 47: Output from GWAS pipeline using Phesant derived variables from UKBiobank | Kg | | |
| Birth weight | PMID: 31043758 | The data of birth weight was collected from the parental or self-reported report, birth register or medical record. | Kg | BOLT-LMM | Gestational age, principal components, array or kinship matrix |
| Childhood BMI | PMID: 26604143 | The discovery analysis included 20 studies with an age range from 3 to 10 years. Different centers had own standard to define recruit the obese child. The Danish Childhood Obesity Biobank defined that children were received from the age of three years if their BMI was above the 90th percentile for sex and age according to the Danish BMI charts. In GOYA study, Cases are defined as having at least one BMI above the age specific 95% percentile and controls as having all BMI below the 50th percentile. | Kg/m^2 | Linear regression model | Principal components if this was deemed needed in the individual studies |
| **Emotional factors** | | | | | |
| Major depressive disorder | PMID: 30718901 | Case and control status of broad depression was defined by the participants' response to the questions 'Have you ever seen a general practitioner for nerves, anxiety, tension or depression?' or 'Have you ever seen a psychiatrist for nerves, anxiety, tension or depression?'. Exclusions were applied to participants who were identified with bipolar disorder, schizophrenia, personality disorder or prescriptions for antipsychotic medications. | Events | BOLT-LMM | Sex, age, genotyping array, and the first 8 principal components |
| Anxious feeling | PMID: 29500382 | Neuroticism was measured using the Eysenck Personality Questionnaire, Revised Short Form (EPQ-R-S)11, consisting of 12 dichotomous items ('yes' or 'no'). Participants completing <9 items were excluded from further analysis. The description of anxious or tense feeling was recorded as "Would you call yourself tense or 'highly strung'?" | Events | Linear regression model | Sex, age, and townsend deprivation index, genotype array, the first 10 principal components |
| Schizophrenia | PMID: 35396580 | EUR schizophrenia patients and controls were obtained from the PGC. All samples of EUR ancestry were included in this study except for the deCODE samples (1513 cases and 66236 controls). | Events | Additive logistic regression model | The first 20 principal components |

| | | | | | |
|---|---|---|---|---|---|
| Bipolar disorder | PMID: 34002096 | Cases were required to meet international consensus criteria (DSM-IV, ICD-9, or ICD-10) for a lifetime diagnosis of bipolar disorder established using structured diagnostic instruments from assessments by trained interviewers, clinician-administered checklists, or medical record review. Controls in most samples were screened for the absence of lifetime psychiatric disorders, as indicated. | Events | Additive logistic regression model | Principal components 1-5 |
| Anorexia nervosa | PMID: 31308545 | The definition of anorexia nervosa established a lifetime diagnosis of anorexia nervosa via hospital or register records, structured clinical interviews, or on-line questionnaires, whereas in the UK Biobank cases self-reported a diagnosis of anorexia nervosa. Controls were carefully matched for ancestry, and some, but not all control cohorts were screened for lifetime eating and/or some or all psychiatric disorders | Events | Additive model | The phenotype in univariate analysis ($P < 0.05$) and the 5 ancestry principal components |
| Life satisfaction | PMID: 30643256 | Life satisfaction refers to a longer-term evaluation of one's life. A typical survey question would be "How satisfied are you with your life as a whole?" | Life satisfaction score | Linear regression model | Sex, z-score of age at the time of assessment, age-squared, the first 4 principal components, study-specific covariates such as study site or batch effects |
| Positive affect | PMID: 30643256 | Positive affect refers to the frequency and intensity of positive emotions and feeling happy. Typical survey questions that gauge positive affect include "During the past week, I was happy?" and "How would you rate your emotional wellbeing at present | Positive affect score | | |
| **Socioeconomic factors** | | | | | |
| Income | MRC-IEU | Dataset: ukb-b-7408; Category: Categorical Ordered; 738: Output from GWAS pipeline using Phesant derived variables from UKBiobank | Dollars | BOLT-LMM | Sex and genotype array |
| Educational attachment | PMID: 30038396 | The Educational years phenotype was constructed by mapping each major educational qualification that can be identified from the cohort's survey measure to an International Standard Classification of Education (ISCED) category and imputing a years-of-education equivalent for each ISCED category. | Years | BOLT-LMM | Age, sex, the first 10 principal components |
| Intelligence | PMID: 29942086 | Different measures of intelligence were assessed in each cohort but were all operationalized to index a common latent g factor underlying multiple dimensions of cognitive functioning. | *g* phenotype | Linear regression model | Age, sex, ancestry principal components |

MRC-IEU, MRC-IEU; BOLT-LMM, association analysis assuming a Bayesian mixture-of-normals prior for the random effect attributed to SNPs other than the one being tested; SAIGE, Scalable and Accurate Implementation of GEneralized mixed model.

## Table S3. The summary of statistical genetic correlation (*P*≤0.05)

| Exposure1 | Exposure2 | Rg | SE | Z-score | *P* value |
|---|---|---|---|---|---|
| 10+ minutes vigorous activity | 10+ minutes vigorous activity | 1 | 2.160E-07 | 4.630E+06 | 0 |
| 25-Hydroxyvitamin D | 25-Hydroxyvitamin D | 1 | 2.050E-07 | 4.880E+06 | 0 |
| 2-hour blood glucose | 2-hour blood glucose | 1 | 2.540E-07 | 3.940E+06 | 0 |
| Adiponectin | Adiponectin | 1 | 2.000E-04 | 6.152E+03 | 0 |
| Adult-onset asthma | Adult-onset asthma | 1 | 3.810E-07 | 2.620E+06 | 0 |
| Age of initiation of regular smoking | Age of initiation of regular smoking | 1 | 8.370E-07 | 1.190E+06 | 0 |
| Alcohol consumption | Alcohol consumption | 1 | 3.810E-06 | 2.625E+05 | 0 |
| Anorexia nervosa | Anorexia nervosa | 1 | 1.520E-08 | 6.590E+07 | 0 |
| Anxious feeling | Anxious feeling | 1 | 4.190E-06 | 2.389E+05 | 0 |
| Anxious feeling | Life satisfaction | -0.718 | 0.018 | -41.082 | 0 |
| Anxious feeling | Positive affect | -0.713 | 0.018 | -40.297 | 0 |
| Atrial fibrillation | Atrial fibrillation | 1 | 6.440E-06 | 1.553E+05 | 0 |
| Bipolar disorder | Bipolar disorder | 1 | 6.760E-07 | 1.480E+06 | 0 |
| Birth weight | Birth weight | 1 | 2.220E-07 | 4.510E+06 | 0 |
| BMI | BMI | 1 | 1.300E-07 | 7.720E+06 | 0 |
| BMI | Visceral adipose tissue | 0.932 | 4.415E-03 | 211.000 | 0 |
| BMI | Whole body fat mass | 0.897 | 5.066E-03 | 177.000 | 0 |
| BMI | Waist circumference | 0.900 | 5.310E-03 | 169.500 | 0 |
| BMI | Body fat percentage | 0.852 | 5.932E-03 | 143.600 | 0 |
| BMI | Hip circumference | 0.849 | 7.572E-03 | 112.100 | 0 |
| Body fat percentage | Body fat percentage | 1 | 4.540E-08 | 2.200E+07 | 0 |
| Body fat percentage | Whole body fat mass | 0.948 | 2.400E-03 | 387.342 | 0 |
| Body fat percentage | Visceral adipose tissue | 0.929 | 5.000E-03 | 184.798 | 0 |
| Body fat percentage | Waist circumference | 0.877 | 5.700E-03 | 152.524 | 0 |
| Body fat percentage | Hip circumference | 0.811 | 8.100E-03 | 99.559 | 0 |
| Breakfast skipping | Breakfast skipping | 1 | 1.080E-06 | 9.236E+05 | 0 |
| Caffeine consumption | Caffeine consumption | 1 | 9.270E-07 | 1.080E+06 | 0 |
| Childhood BMI | Childhood BMI | 1 | 8.930E-07 | 1.120E+06 | 0 |
| Childhood-onset asthma | Childhood-onset asthma | 1 | 1.200E-05 | 8.349E+04 | 0 |
| Cigarette consumption | Cigarette consumption | 1 | 5.900E-07 | 1.700E+06 | 0 |
| Coffee consumption | Coffee consumption | 1 | 1.890E-06 | 5.278E+05 | 0 |
| Coffee consumption | Caffeine consumption | 0.950 | 8.400E-03 | 113.101 | 0 |
| Coronary artery disease | Coronary artery disease | 1 | 2.040E-07 | 4.900E+06 | 0 |
| Crohn's disease | Crohn's disease | 1 | 3.010E-08 | 3.320E+07 | 0 |
| Daytime napping | Daytime napping | 1 | 1.830E-07 | 5.460E+06 | 0 |
| Daytime sleepiness | Daytime sleepiness | 1 | 1.400E-08 | 7.120E+07 | 0 |
| Daytime sleepiness | Daytime napping | 0.701 | 0.017 | 42.500 | 0 |
| Educational attainment | Educational attainment | 1 | 2.710E-08 | 3.690E+07 | 0 |
| Fasting glucose | Fasting glucose | 1 | 2.470E-06 | 4.051E+05 | 0 |
| Fasting insulin | Fasting insulin | 1 | 4.530E-08 | 2.210E+07 | 0 |

| | | | | | |
|---|---|---|---|---|---|
| Hand grip strength (left) | Hand grip strength (left) | 1 | 1.790E-07 | 5.600E+06 | 0 |
| Hand grip strength (left) | Hand grip strength (right) | 0.981 | 2.900E-03 | 341.203 | 0 |
| Hand grip strength (right) | Hand grip strength (right) | 1 | 3.020E-07 | 3.320E+06 | 0 |
| HbA1c | HbA1c | 1 | 1.700E-06 | 5.887E+05 | 0 |
| HDL cholesterol | HDL cholesterol | 1 | 5.810E-06 | 1.722E+05 | 0 |
| Hip circumference | Hip circumference | 1 | 8.000E-08 | 1.250E+07 | 0 |
| Income | Income | 1 | 4.200E-08 | 2.380E+07 | 0 |
| Income | Educational attainment | 0.804 | 0.012 | 67.234 | 0 |
| Insomnia | Insomnia | 1 | 2.590E-07 | 3.860E+06 | 0 |
| Intake of carbohydrate | Intake of carbohydrate | 1 | 5.510E-08 | 1.820E+07 | 0 |
| Intake of fat | Intake of fat | 1 | 3.180E-07 | 3.140E+06 | 0 |
| Intake of total sugar | Intake of total sugar | 1 | 1.330E-06 | 7.500E+05 | 0 |
| Intelligence | Intelligence | 1 | 1.010E-06 | 9.857E+05 | 0 |
| Intelligence | Educational attainment | 0.807 | 0.011 | 65.687 | 0 |
| LDL cholesterol | LDL cholesterol | 1 | 2.000E-04 | 4.559E+03 | 0 |
| LDL cholesterol | Total cholesterol | 0.969 | 0.022 | 46.387 | 0 |
| Leisure sedentary behaviour (computer use) | Leisure sedentary behaviour (computer use) | 1 | 1.430E-07 | 7.000E+06 | 0 |
| Leisure sedentary behaviour (TV watching) | Leisure sedentary behaviour (TV watching) | 1 | 6.860E-08 | 1.460E+07 | 0 |
| Leisure sedentary behaviour (TV watching) | Educational attainment | -0.667 | 0.012 | -54.600 | 0 |
| Leptin | Leptin | 1 | 1.310E-06 | 7.659E+05 | 0 |
| Life satisfaction | Life satisfaction | 1 | 5.470E-06 | 1.828E+05 | 0 |
| Life satisfaction | Positive affect | 0.975 | 2.900E-03 | 341.356 | 0 |
| Lifetime smoking | Lifetime smoking | 1 | 1.050E-06 | 9.496E+05 | 0 |
| Long sleep | Long sleep | 1 | 3.690E-07 | 2.710E+06 | 0 |
| Major depressive disorder | Major depressive disorder | 1 | 5.000E-09 | 2.000E+08 | 0 |
| Major depressive disorder | Life satisfaction | -0.859 | 0.011 | -81.156 | 0 |
| Major depressive disorder | Positive affect | -0.844 | 0.011 | -74.688 | 0 |
| Moderate to vigorous physical activity levels | Moderate to vigorous physical activity levels | 1 | 2.430E-07 | 4.120E+06 | 0 |
| Moderate to vigorous physical activity levels | 10+ minutes vigorous activity | 0.853 | 0.015 | 58.800 | 0 |
| Morning person | Morning person | 1 | 7.930E-09 | 1.260E+08 | 0 |
| Positive affect | Positive affect | 1 | 1.570E-06 | 6.372E+05 | 0 |
| Schizophrenia | Schizophrenia | 1 | 1.150E-07 | 8.710E+06 | 0 |
| Schizophrenia | Bipolar disorder | 0.684 | 0.017 | 41.381 | 0 |
| Short sleep | Short sleep | 1 | 2.720E-07 | 3.670E+06 | 0 |
| Sitting height | Sitting height | 1 | 2.270E-06 | 4.407E+05 | 0 |
| Smoking cessation | Smoking cessation | 1 | 6.930E-07 | 1.440E+06 | 0 |
| Smoking initiation | Smoking initiation | 1 | 5.200E-07 | 1.920E+06 | 0 |
| Smoking initiation | Lifetime smoking | 0.869 | 0.011 | 77.467 | 0 |
| Standing height | Standing height | 1 | 3.570E-06 | 2.803E+05 | 0 |

| | | | | | |
|---|---|---|---|---|---|
| Standing height | Sitting height | 0.846 | 6.600E-03 | 129.010 | 0 |
| Strenuous sports or other exercises | Strenuous sports or other exercises | 1 | 1.910E-06 | 5.227E+05 | 0 |
| Total cholesterol | Total cholesterol | 1 | 3.000E-04 | 2.970E+03 | 0 |
| Triglycerides | Triglycerides | 1 | 4.560E-05 | 2.191E+04 | 0 |
| Type 2 diabetes | Type 2 diabetes | 1 | 6.490E-09 | 1.540E+08 | 0 |
| Ulcerative colitis | Ulcerative colitis | 1 | 6.450E-07 | 1.550E+06 | 0 |
| Visceral adipose tissue | Visceral adipose tissue | 1 | 3.940E-07 | 2.540E+06 | 0 |
| Visceral adipose tissue | Waist circumference | 0.979 | 2.600E-03 | 369.786 | 0 |
| Visceral adipose tissue | Hip circumference | 0.829 | 0.010 | 82.933 | 0 |
| Visceral adipose tissue | Waist-to-hip ratio | 0.790 | 0.012 | 67.832 | 0 |
| Vitamin C | Vitamin C | 1 | 4.940E-06 | 2.023E+05 | 0 |
| Waist circumference | Waist circumference | 1 | 1.110E-07 | 8.990E+06 | 0 |
| Waist circumference | Hip circumference | 0.868 | 7.400E-03 | 117.960 | 0 |
| Waist circumference | Waist-to-hip ratio | 0.777 | 0.010 | 75.845 | 0 |
| Waist-to-hip ratio | Waist-to-hip ratio | 1 | 7.620E-09 | 1.310E+08 | 0 |
| Whole body fat mass | Whole body fat mass | 1 | 1.060E-07 | 9.400E+06 | 0 |
| Whole body fat mass | Waist circumference | 0.947 | 3.300E-03 | 283.106 | 0 |
| Whole body fat mass | Hip circumference | 0.927 | 3.300E-03 | 283.153 | 0 |
| Whole body fat mass | Visceral adipose tissue | 0.959 | 3.800E-03 | 250.087 | 0 |
| Whole body fat mass | Whole body fat-free mass | 0.646 | 0.012 | 54.756 | 0 |
| Whole body fat-free mass | Whole body fat-free mass | 1 | 8.360E-07 | 1.200E+06 | 0 |
| Whole body fat-free mass | Hip circumference | 0.764 | 9.100E-03 | 84.395 | 0 |
| Whole body fat-free mass | Waist circumference | 0.660 | 0.012 | 55.764 | 0 |
| Whole body fat-free mass | Sitting height | 0.650 | 0.013 | 50.285 | 0 |
| Whole body fat-free mass | Standing height | 0.643 | 0.013 | 48.033 | 0 |
| 10+ minutes vigorous activity | Strenuous sports or other exercises | 0.698 | 0.019 | 36.100 | 3.100E-285 |
| Whole body fat-free mass | Visceral adipose tissue | 0.568 | 0.016 | 35.950 | 5.160E-283 |
| Intake of total sugar | Intake of carbohydrate | 0.730 | 0.021 | 35.539 | 1.230E-276 |
| BMI | Whole body fat-free mass | 0.572 | 0.017 | 33.940 | 1.450E-252 |
| BMI | Waist-to-hip ratio | 0.610 | 0.018 | 33.880 | 1.360E-251 |
| Insomnia | Short sleep | 0.645 | 0.019 | 33.473 | 1.210E-245 |
| Leisure sedentary behaviour (computer use) | Educational attainment | 0.508 | 0.016 | 32.600 | 1.910E-232 |
| Lifetime smoking | Age of initiation of regular smoking | -0.674 | 0.021 | -31.880 | 5.050E-223 |
| Lifetime smoking | Educational attainment | -0.471 | 0.015 | -31.637 | 1.160E-219 |
| Whole body fat mass | Waist-to-hip ratio | 0.608 | 0.020 | 31.178 | 2.100E-213 |
| Body fat percentage | Waist-to-hip ratio | 0.621 | 0.021 | 30.124 | 2.350E-199 |
| Smoking initiation | Age of initiation of regular smoking | -0.684 | 0.023 | -30.115 | 3.090E-199 |
| Major depressive disorder | Anxious feeling | 0.648 | 0.022 | 30.022 | 5.010E-198 |
| Leisure sedentary behaviour (TV watching) | Income | -0.574 | 0.019 | -29.600 | 1.560E-192 |
| Type 2 diabetes | Waist circumference | 0.602 | 0.021 | 29.053 | 1.400E-185 |
| Type 2 diabetes | Waist-to-hip ratio | 0.584 | 0.020 | 28.943 | 3.420E-184 |
| Leisure sedentary behaviour (TV | Visceral adipose tissue | 0.463 | 0.017 | 27.900 | 7.840E-172 |

| | | | | | |
|---|---|---|---|---|---|
| watching) | | | | | |
| Standing height | Hand grip strength (right) | 0.460 | 0.017 | 27.791 | 5.630E-170 |
| Age of initiation of regular smoking | Educational attainment | 0.599 | 0.022 | 27.531 | 7.400E-167 |
| Whole body fat-free mass | Hand grip strength (left) | 0.409 | 0.015 | 27.240 | 2.190E-163 |
| Leisure sedentary behaviour (TV watching) | Body fat percentage | 0.436 | 0.016 | 27.000 | 1.530E-160 |
| Intelligence | Leisure sedentary behaviour (computer use) | 0.501 | 0.019 | 26.793 | 3.920E-158 |
| Lifetime smoking | Smoking cessation | 0.658 | 0.025 | 26.702 | 4.440E-157 |
| Type 2 diabetes | Visceral adipose tissue | 0.614 | 0.023 | 26.667 | 1.130E-156 |
| Standing height | Hand grip strength (left) | 0.429 | 0.016 | 26.509 | 7.670E-155 |
| Waist-to-hip ratio | Educational attainment | -0.313 | 0.012 | -26.360 | 3.910E-153 |
| Strenuous sports or other exercises | Educational attainment | 0.508 | 0.019 | 26.350 | 5.160E-153 |
| Visceral adipose tissue | Educational attainment | -0.381 | 0.015 | -26.302 | 1.820E-152 |
| Lifetime smoking | Cigarette consumption | 0.545 | 0.021 | 26.225 | 1.370E-151 |
| Body fat percentage | Educational attainment | -0.342 | 0.013 | -25.914 | 4.630E-148 |
| Leisure sedentary behaviour (computer use) | Income | 0.498 | 0.019 | 25.700 | 3.100E-145 |
| Whole body fat-free mass | Hand grip strength (right) | 0.422 | 0.017 | 25.510 | 1.540E-143 |
| Intelligence | Leisure sedentary behaviour (TV watching) | -0.442 | 0.017 | -25.390 | 3.250E-142 |
| BMI | Educational attainment | -0.333 | 0.013 | -25.220 | 2.550E-140 |
| BMI | Type 2 diabetes | 0.566 | 0.023 | 25.043 | 2.100E-138 |
| Lifetime smoking | Income | -0.452 | 0.019 | -24.448 | 5.250E-132 |
| Smoking initiation | Educational attainment | -0.362 | 0.015 | -24.094 | 2.860E-128 |
| Leisure sedentary behaviour (TV watching) | Whole body fat mass | 0.397 | 0.017 | 23.700 | 3.070E-124 |
| Lifetime smoking | Waist circumference | 0.352 | 0.015 | 23.604 | 3.510E-123 |
| Leisure sedentary behaviour (TV watching) | Waist circumference | 0.388 | 0.017 | 23.600 | 7.230E-123 |
| BMI | Leisure sedentary behaviour (TV watching) | 0.404 | 0.017 | 23.370 | 8.480E-121 |
| BMI | Lifetime smoking | 0.354 | 0.015 | 23.210 | 3.290E-119 |
| Sitting height | Hand grip strength (right) | 0.414 | 0.018 | 23.163 | 1.080E-118 |
| Hip circumference | Sitting height | 0.347 | 0.015 | 23.059 | 1.190E-117 |
| Lifetime smoking | Visceral adipose tissue | 0.401 | 0.018 | 22.866 | 1.020E-115 |
| Lifetime smoking | Body fat percentage | 0.327 | 0.014 | 22.715 | 3.200E-114 |
| Waist circumference | Educational attainment | -0.294 | 0.013 | -22.600 | 4.350E-113 |
| Lifetime smoking | Whole body fat mass | 0.325 | 0.014 | 22.504 | 3.770E-112 |
| Type 2 diabetes | Whole body fat mass | 0.540 | 0.024 | 22.479 | 6.600E-112 |
| Strenuous sports or other exercises | Income | 0.542 | 0.024 | 22.322 | 2.270E-110 |
| Insomnia | Life satisfaction | -0.451 | 0.020 | -22.266 | 7.950E-110 |
| Whole body fat mass | Educational attainment | -0.294 | 0.013 | -22.173 | 6.330E-109 |
| Sitting height | Hand grip strength (left) | 0.384 | 0.017 | 22.106 | 2.800E-108 |

| | | | | | |
|---|---|---|---|---|---|
| Insomnia | Positive affect | -0.446 | 0.020 | -22.051 | 9.250E-108 |
| Leisure sedentary behaviour (TV watching) | Waist-to-hip ratio | 0.352 | 0.017 | 21.300 | 1.690E-100 |
| Alcohol consumption | Intake of carbohydrate | -0.644 | 0.030 | -21.270 | 1.970E-100 |
| Type 2 diabetes | Body fat percentage | 0.525 | 0.025 | 21.106 | 7.040E-99 |
| Lifetime smoking | Waist-to-hip ratio | 0.349 | 0.017 | 21.096 | 8.600E-99 |
| Whole body fat-free mass | Birth weight | 0.370 | 0.018 | 21.057 | 1.990E-98 |
| BMI | Childhood BMI | 0.641 | 0.031 | 20.950 | 1.900E-97 |
| Bipolar disorder | Major depressive disorder | 0.451 | 0.022 | 20.708 | 2.940E-95 |
| Smoking cessation | Educational attainment | -0.502 | 0.024 | -20.689 | 4.310E-95 |
| Insomnia | Major depressive disorder | 0.458 | 0.022 | 20.530 | 1.150E-93 |
| Alcohol consumption | Smoking initiation | 0.407 | 0.020 | 20.400 | 1.710E-92 |
| Short sleep | Educational attainment | -0.358 | 0.018 | -19.859 | 9.310E-88 |
| Waist-to-hip ratio | Income | -0.318 | 0.016 | -19.816 | 2.150E-87 |
| Standing height | Birth weight | 0.379 | 0.019 | 19.589 | 1.930E-85 |
| Childhood-onset asthma | Adult-onset asthma | 0.737 | 0.038 | 19.461 | 2.350E-84 |
| Body fat percentage | Whole body fat-free mass | 0.369 | 0.019 | 19.252 | 1.350E-82 |
| Hip circumference | Standing height | 0.298 | 0.016 | 18.997 | 1.810E-80 |
| Coronary artery disease | Waist-to-hip ratio | 0.365 | 0.019 | 18.993 | 1.960E-80 |
| Intake of fat | Intake of carbohydrate | -0.602 | 0.032 | -18.891 | 1.350E-79 |
| Leisure sedentary behaviour (TV watching) | Strenuous sports or other exercises | -0.467 | 0.025 | -18.800 | 7.150E-79 |
| Intelligence | Moderate to vigorous physical activity levels | -0.438 | 0.024 | -18.520 | 1.420E-76 |
| Type 2 diabetes | Coronary artery disease | 0.406 | 0.022 | 18.431 | 7.440E-76 |
| Lifetime smoking | Leisure sedentary behaviour (TV watching) | 0.380 | 0.021 | 18.119 | 2.270E-73 |
| Hip circumference | Childhood BMI | 0.563 | 0.031 | 18.101 | 3.140E-73 |
| Age of initiation of regular smoking | Income | 0.532 | 0.029 | 18.081 | 4.530E-73 |
| BMI | Income | -0.297 | 0.017 | -17.970 | 3.320E-72 |
| Visceral adipose tissue | Income | -0.345 | 0.019 | -17.768 | 1.260E-70 |
| Age of initiation of regular smoking | Leisure sedentary behaviour (TV watching) | -0.456 | 0.026 | -17.374 | 1.300E-67 |
| Alcohol consumption | Lifetime smoking | 0.341 | 0.020 | 17.080 | 1.950E-65 |
| Schizophrenia | Life satisfaction | -0.339 | 0.020 | -17.083 | 1.980E-65 |
| Body fat percentage | Income | -0.297 | 0.018 | -16.885 | 5.850E-64 |
| Whole body fat mass | Childhood BMI | 0.534 | 0.032 | 16.869 | 7.600E-64 |
| Schizophrenia | Positive affect | -0.342 | 0.020 | -16.835 | 1.340E-63 |
| Smoking initiation | Visceral adipose tissue | 0.303 | 0.018 | 16.680 | 1.830E-62 |
| Smoking initiation | Income | -0.321 | 0.019 | -16.651 | 3.000E-62 |
| Coronary artery disease | Waist circumference | 0.330 | 0.020 | 16.597 | 7.340E-62 |
| Smoking initiation | Waist-to-hip ratio | 0.257 | 0.016 | 16.590 | 8.280E-62 |
| Leisure sedentary behaviour (TV watching) | Hip circumference | 0.297 | 0.018 | 16.500 | 5.000E-61 |

| | | | | | |
|---|---|---|---|---|---|
| Lifetime smoking | Hip circumference | 0.242 | 0.015 | 16.160 | 9.740E-59 |
| Intake of carbohydrate | Breakfast skipping | -0.536 | 0.033 | -16.149 | 1.160E-58 |
| Waist circumference | Income | -0.269 | 0.017 | -16.113 | 2.070E-58 |
| Whole body fat mass | Sitting height | 0.219 | 0.014 | 15.946 | 3.060E-57 |
| Sitting height | Birth weight | 0.340 | 0.021 | 15.941 | 3.280E-57 |
| Bipolar disorder | Life satisfaction | -0.385 | 0.024 | -15.927 | 4.140E-57 |
| Long sleep | Daytime napping | 0.421 | 0.026 | 15.926 | 4.200E-57 |
| Waist circumference | Childhood BMI | 0.517 | 0.033 | 15.903 | 6.040E-57 |
| Type 2 diabetes | Hip circumference | 0.435 | 0.027 | 15.863 | 1.140E-56 |
| Leisure sedentary behaviour (TV watching) | Leisure sedentary behaviour (computer use) | -0.316 | 0.020 | -15.800 | 2.780E-56 |
| Lifetime smoking | Major depressive disorder | 0.336 | 0.021 | 15.795 | 3.370E-56 |
| BMI | Smoking initiation | 0.259 | 0.016 | 15.790 | 3.860E-56 |
| Smoking cessation | Income | -0.480 | 0.031 | -15.759 | 5.950E-56 |
| Smoking initiation | Waist circumference | 0.266 | 0.017 | 15.722 | 1.080E-55 |
| Long sleep | Educational attainment | -0.351 | 0.022 | -15.618 | 5.540E-55 |
| Coronary artery disease | Educational attainment | -0.260 | 0.017 | -15.539 | 1.900E-54 |
| BMI | Coronary artery disease | 0.325 | 0.021 | 15.497 | 3.620E-54 |
| Coronary artery disease | Visceral adipose tissue | 0.375 | 0.024 | 15.442 | 8.590E-54 |
| Smoking initiation | Whole body fat mass | 0.247 | 0.016 | 15.265 | 1.300E-52 |
| Schizophrenia | Anxious feeling | 0.365 | 0.024 | 15.185 | 4.470E-52 |
| Schizophrenia | Major depressive disorder | 0.324 | 0.021 | 15.180 | 4.770E-52 |
| Standing height | Educational attainment | 0.182 | 0.012 | 15.108 | 1.440E-51 |
| Insomnia | Educational attainment | -0.268 | 0.018 | -15.035 | 4.310E-51 |
| Type 2 diabetes | Educational attainment | -0.252 | 0.017 | -14.878 | 4.580E-50 |
| Visceral adipose tissue | Childhood BMI | 0.526 | 0.035 | 14.853 | 6.630E-50 |
| Bipolar disorder | Positive affect | -0.376 | 0.025 | -14.838 | 8.300E-50 |
| Smoking initiation | Body fat percentage | 0.238 | 0.016 | 14.784 | 1.860E-49 |
| Lifetime smoking | Breakfast skipping | 0.450 | 0.031 | 14.684 | 8.230E-49 |
| Atrial fibrillation | Whole body fat-free mass | 0.307 | 0.021 | 14.672 | 9.790E-49 |
| Smoking initiation | Major depressive disorder | 0.309 | 0.021 | 14.582 | 3.670E-48 |
| BMI | Anorexia nervosa | -0.329 | 0.023 | -14.560 | 5.390E-48 |
| Age of initiation of regular smoking | Waist-to-hip ratio | -0.317 | 0.022 | -14.526 | 8.310E-48 |
| Strenuous sports or other exercises | Body fat percentage | -0.298 | 0.021 | -14.479 | 1.640E-47 |
| Short sleep | Income | -0.355 | 0.025 | -14.443 | 2.790E-47 |
| Body fat percentage | Childhood BMI | 0.460 | 0.032 | 14.440 | 2.890E-47 |
| Ulcerative colitis | Crohn's disease | 0.701 | 0.049 | 14.409 | 4.570E-47 |
| Bipolar disorder | Anxious feeling | 0.369 | 0.026 | 14.402 | 5.000E-47 |
| Type 2 diabetes | Lifetime smoking | 0.252 | 0.018 | 14.376 | 7.350E-47 |
| Intelligence | Waist-to-hip ratio | -0.205 | 0.014 | -14.370 | 8.010E-47 |
| Smoking initiation | Intake of carbohydrate | -0.386 | 0.027 | -14.311 | 1.880E-46 |
| Intelligence | Age of initiation of regular smoking | 0.361 | 0.025 | 14.237 | 5.390E-46 |
| Intelligence | Long sleep | -0.380 | 0.027 | -14.171 | 1.380E-45 |
| Whole body fat mass | Standing height | 0.198 | 0.014 | 14.163 | 1.560E-45 |

| | | | | | |
|---|---|---|---|---|---|
| Standing height | Income | 0.233 | 0.016 | 14.160 | 1.610E-45 |
| Anorexia nervosa | Body fat percentage | -0.347 | 0.025 | -14.158 | 1.660E-45 |
| Anorexia nervosa | Whole body fat mass | -0.336 | 0.024 | -14.018 | 1.210E-44 |
| Strenuous sports or other exercises | Visceral adipose tissue | -0.326 | 0.024 | -13.911 | 5.460E-44 |
| Coronary artery disease | Body fat percentage | 0.310 | 0.022 | 13.858 | 1.140E-43 |
| Coronary artery disease | Lifetime smoking | 0.293 | 0.021 | 13.775 | 3.630E-43 |
| Whole body fat mass | Income | -0.237 | 0.017 | -13.766 | 4.080E-43 |
| Hip circumference | Educational attainment | -0.190 | 0.014 | -13.740 | 5.830E-43 |
| Anorexia nervosa | Waist circumference | -0.334 | 0.024 | -13.721 | 7.630E-43 |
| Atrial fibrillation | Hip circumference | 0.284 | 0.021 | 13.703 | 9.740E-43 |
| Cigarette consumption | Smoking cessation | 0.440 | 0.032 | 13.641 | 2.290E-42 |
| Hip circumference | Waist-to-hip ratio | 0.368 | 0.027 | 13.576 | 5.520E-42 |
| Alcohol consumption | Intake of total sugar | -0.423 | 0.031 | -13.550 | 7.470E-42 |
| BMI | Age of initiation of regular smoking | -0.307 | 0.023 | -13.510 | 1.380E-41 |
| Anorexia nervosa | Visceral adipose tissue | -0.354 | 0.026 | -13.509 | 1.390E-41 |
| Coronary artery disease | Whole body fat mass | 0.285 | 0.021 | 13.405 | 5.680E-41 |
| Age of initiation of regular smoking | Body fat percentage | -0.295 | 0.022 | -13.358 | 1.070E-40 |
| Smoking initiation | Smoking cessation | 0.391 | 0.029 | 13.320 | 1.770E-40 |
| Age of initiation of regular smoking | Visceral adipose tissue | -0.356 | 0.027 | -13.309 | 2.060E-40 |
| Coffee consumption | Waist circumference | 0.293 | 0.022 | 13.220 | 6.720E-40 |
| Short sleep | Leisure sedentary behaviour (TV watching) | 0.313 | 0.024 | 13.198 | 8.970E-40 |
| Intake of total sugar | Breakfast skipping | -0.531 | 0.040 | -13.185 | 1.070E-39 |
| Lifetime smoking | Intake of carbohydrate | -0.356 | 0.027 | -13.178 | 1.170E-39 |
| Major depressive disorder | Income | -0.306 | 0.023 | -13.173 | 1.250E-39 |
| Age of initiation of regular smoking | Waist circumference | -0.300 | 0.023 | -13.172 | 1.280E-39 |
| Smoking initiation | Breakfast skipping | 0.403 | 0.031 | 13.135 | 2.070E-39 |
| Type 2 diabetes | Leisure sedentary behaviour (TV watching) | 0.287 | 0.022 | 13.128 | 2.290E-39 |
| Anorexia nervosa | Hip circumference | -0.302 | 0.023 | -13.114 | 2.750E-39 |
| Moderate to vigorous physical activity levels | Strenuous sports or other exercises | 0.347 | 0.026 | 13.100 | 3.010E-39 |
| Smoking initiation | Leisure sedentary behaviour (TV watching) | 0.275 | 0.021 | 13.079 | 4.340E-39 |
| Intelligence | Visceral adipose tissue | -0.233 | 0.018 | -13.066 | 5.180E-39 |
| Insomnia | Leisure sedentary behaviour (TV watching) | 0.279 | 0.022 | 13.007 | 1.120E-38 |
| Leptin | Body fat percentage | 0.829 | 0.064 | 12.960 | 2.070E-38 |
| Lifetime smoking | Short sleep | 0.333 | 0.026 | 12.956 | 2.170E-38 |
| BMI | Coffee consumption | 0.301 | 0.023 | 12.920 | 3.320E-38 |
| Leptin | Whole body fat mass | 0.814 | 0.063 | 12.882 | 5.710E-38 |
| Lifetime smoking | Life satisfaction | -0.280 | 0.022 | -12.842 | 9.580E-38 |
| Leptin | Visceral adipose tissue | 0.791 | 0.062 | 12.799 | 1.660E-37 |
| Intelligence | Lifetime smoking | -0.252 | 0.020 | -12.765 | 2.570E-37 |

| | | | | | |
|---|---|---|---|---|---|
| Smoking initiation | Life satisfaction | -0.252 | 0.020 | -12.730 | 4.020E-37 |
| Intake of total sugar | Intake of fat | -0.511 | 0.040 | -12.693 | 6.480E-37 |
| Whole body fat-free mass | Childhood BMI | 0.447 | 0.035 | 12.690 | 6.750E-37 |
| Whole body fat-free mass | Waist-to-hip ratio | 0.277 | 0.022 | 12.641 | 1.250E-36 |
| Life satisfaction | Income | 0.318 | 0.025 | 12.624 | 1.560E-36 |
| Type 2 diabetes | Whole body fat-free mass | 0.303 | 0.024 | 12.607 | 1.930E-36 |
| Insomnia | Anxious feeling | 0.326 | 0.026 | 12.569 | 3.140E-36 |
| Smoking cessation | Leisure sedentary behaviour (TV watching) | 0.388 | 0.031 | 12.534 | 4.850E-36 |
| Coffee consumption | Whole body fat mass | 0.283 | 0.023 | 12.529 | 5.210E-36 |
| Leisure sedentary behaviour (computer use) | Moderate to vigorous physical activity levels | -0.329 | 0.026 | -12.500 | 8.880E-36 |
| Atrial fibrillation | Whole body fat mass | 0.256 | 0.021 | 12.473 | 1.040E-35 |
| Type 2 diabetes | HbA1c | 0.445 | 0.036 | 12.452 | 1.360E-35 |
| Insomnia | Income | -0.292 | 0.024 | -12.421 | 2.000E-35 |
| Coffee consumption | Hip circumference | 0.306 | 0.025 | 12.352 | 4.740E-35 |
| Strenuous sports or other exercises | Whole body fat mass | -0.258 | 0.021 | -12.351 | 4.790E-35 |
| Smoking initiation | Positive affect | -0.243 | 0.020 | -12.348 | 5.000E-35 |
| Positive affect | Income | 0.308 | 0.025 | 12.333 | 6.040E-35 |
| Intelligence | BMI | -0.181 | 0.015 | -12.328 | 6.370E-35 |
| Lifetime smoking | Strenuous sports or other exercises | -0.305 | 0.025 | -12.328 | 6.400E-35 |
| Lifetime smoking | Positive affect | -0.271 | 0.022 | -12.281 | 1.140E-34 |
| Cigarette consumption | Income | -0.286 | 0.023 | -12.278 | 1.190E-34 |
| Leptin | Waist circumference | 0.774 | 0.063 | 12.205 | 2.920E-34 |
| Short sleep | Major depressive disorder | 0.309 | 0.026 | 12.028 | 2.530E-33 |
| Intelligence | Body fat percentage | -0.191 | 0.016 | -12.025 | 2.630E-33 |
| Strenuous sports or other exercises | Waist-to-hip ratio | -0.258 | 0.022 | -11.923 | 9.030E-33 |
| Caffeine consumption | Educational attainment | 0.244 | 0.021 | 11.920 | 9.350E-33 |
| Atrial fibrillation | Waist circumference | 0.251 | 0.021 | 11.910 | 1.050E-32 |
| Short sleep | Life satisfaction | -0.284 | 0.024 | -11.891 | 1.330E-32 |
| Lifetime smoking | Insomnia | 0.275 | 0.023 | 11.885 | 1.410E-32 |
| Strenuous sports or other exercises | Waist circumference | -0.266 | 0.022 | -11.883 | 1.450E-32 |
| Cigarette consumption | Waist circumference | 0.372 | 0.031 | 11.882 | 1.480E-32 |
| Atrial fibrillation | Standing height | 0.213 | 0.018 | 11.843 | 2.340E-32 |
| BMI | Leptin | 0.770 | 0.065 | 11.810 | 3.640E-32 |
| BMI | Cigarette consumption | 0.350 | 0.030 | 11.800 | 3.840E-32 |
| Age of initiation of regular smoking | Whole body fat mass | -0.276 | 0.023 | -11.796 | 4.090E-32 |
| Hip circumference | Birth weight | 0.201 | 0.017 | 11.792 | 4.320E-32 |
| Daytime napping | Waist circumference | 0.183 | 0.016 | 11.800 | 4.890E-32 |
| Daytime napping | Waist-to-hip ratio | 0.181 | 0.015 | 11.700 | 8.790E-32 |
| Leptin | Hip circumference | 0.741 | 0.064 | 11.576 | 5.430E-31 |
| Coronary artery disease | Leisure sedentary behaviour (TV watching) | 0.262 | 0.023 | 11.574 | 5.560E-31 |
| Smoking initiation | Hip circumference | 0.184 | 0.016 | 11.475 | 1.760E-30 |

| | | | | | |
|---|---|---|---|---|---|
| Cigarette consumption | Hip circumference | 0.309 | 0.027 | 11.410 | 3.720E-30 |
| Short sleep | Visceral adipose tissue | 0.260 | 0.023 | 11.381 | 5.180E-30 |
| Atrial fibrillation | Sitting height | 0.212 | 0.019 | 11.363 | 6.390E-30 |
| BMI | Smoking cessation | 0.285 | 0.025 | 11.330 | 9.360E-30 |
| Short sleep | Positive affect | -0.276 | 0.025 | -11.279 | 1.670E-29 |
| Long sleep | Income | -0.373 | 0.033 | -11.224 | 3.120E-29 |
| Alcohol consumption | Breakfast skipping | 0.321 | 0.029 | 11.200 | 3.870E-29 |
| Cigarette consumption | Educational attainment | -0.285 | 0.026 | -11.135 | 8.440E-29 |
| Cigarette consumption | Whole body fat mass | 0.345 | 0.031 | 11.103 | 1.210E-28 |
| Sitting height | Educational attainment | 0.143 | 0.013 | 11.091 | 1.380E-28 |
| Intelligence | Standing height | 0.153 | 0.014 | 11.056 | 2.060E-28 |
| Short sleep | Strenuous sports or other exercises | -0.293 | 0.027 | -11.038 | 2.490E-28 |
| Short sleep | Waist-to-hip ratio | 0.222 | 0.020 | 11.031 | 2.720E-28 |
| Hand grip strength (right) | Birth weight | 0.240 | 0.022 | 11.026 | 2.850E-28 |
| Anorexia nervosa | Waist-to-hip ratio | -0.251 | 0.023 | -11.006 | 3.600E-28 |
| Coronary artery disease | Smoking initiation | 0.223 | 0.021 | 10.909 | 1.040E-27 |
| Sitting height | Income | 0.203 | 0.019 | 10.896 | 1.210E-27 |
| BMI | Short sleep | 0.249 | 0.023 | 10.880 | 1.470E-27 |
| Daytime napping | Whole body fat mass | 0.170 | 0.016 | 10.900 | 1.590E-27 |
| Cigarette consumption | Body fat percentage | 0.330 | 0.030 | 10.846 | 2.090E-27 |
| Daytime napping | Body fat percentage | 0.175 | 0.016 | 10.800 | 2.220E-27 |
| Hand grip strength (left) | Birth weight | 0.232 | 0.022 | 10.750 | 5.940E-27 |
| Coffee consumption | Visceral adipose tissue | 0.256 | 0.024 | 10.718 | 8.410E-27 |
| Waist circumference | Standing height | 0.149 | 0.014 | 10.706 | 9.530E-27 |
| Smoking cessation | Strenuous sports or other exercises | -0.397 | 0.037 | -10.696 | 1.060E-26 |
| Smoking initiation | Short sleep | 0.236 | 0.022 | 10.695 | 1.070E-26 |
| 10+ minutes vigorous activity | Body fat percentage | -0.219 | 0.021 | -10.600 | 2.740E-26 |
| Coffee consumption | Body fat percentage | 0.227 | 0.022 | 10.542 | 5.560E-26 |
| Intelligence | Smoking cessation | -0.303 | 0.029 | -10.529 | 6.380E-26 |
| Smoking cessation | Waist circumference | 0.265 | 0.025 | 10.418 | 2.040E-25 |
| BMI | Strenuous sports or other exercises | -0.232 | 0.022 | -10.400 | 2.390E-25 |
| Waist circumference | Sitting height | 0.147 | 0.014 | 10.401 | 2.460E-25 |
| Coronary artery disease | Insomnia | 0.242 | 0.023 | 10.367 | 3.510E-25 |
| Coffee consumption | Whole body fat-free mass | 0.283 | 0.027 | 10.338 | 4.760E-25 |
| Insomnia | Strenuous sports or other exercises | -0.268 | 0.026 | -10.334 | 4.950E-25 |
| Coronary artery disease | Major depressive disorder | 0.207 | 0.020 | 10.330 | 5.140E-25 |
| Type 2 diabetes | HDL cholesterol | -0.431 | 0.042 | -10.306 | 6.590E-25 |
| Type 2 diabetes | Daytime napping | 0.189 | 0.018 | 10.279 | 8.800E-25 |
| Coronary artery disease | Atrial fibrillation | 0.241 | 0.024 | 10.274 | 9.220E-25 |
| Daytime napping | Visceral adipose tissue | 0.205 | 0.020 | 10.300 | 1.060E-24 |
| HDL cholesterol | Waist-to-hip ratio | -0.425 | 0.041 | -10.258 | 1.090E-24 |
| Cigarette consumption | Waist-to-hip ratio | 0.299 | 0.029 | 10.234 | 1.390E-24 |
| HDL cholesterol | Visceral adipose tissue | -0.398 | 0.039 | -10.218 | 1.640E-24 |
| Schizophrenia | Long sleep | 0.310 | 0.030 | 10.218 | 1.650E-24 |

| | | | | | |
|---|---|---|---|---|---|
| Short sleep | Waist circumference | 0.232 | 0.023 | 10.194 | 2.100E-24 |
| Smoking cessation | Visceral adipose tissue | 0.297 | 0.029 | 10.194 | 2.100E-24 |
| Coronary artery disease | Income | -0.238 | 0.023 | -10.180 | 2.430E-24 |
| Type 2 diabetes | Cigarette consumption | 0.307 | 0.030 | 10.174 | 2.600E-24 |
| Anorexia nervosa | Positive affect | -0.295 | 0.029 | -10.165 | 2.830E-24 |
| Age of initiation of regular smoking | Strenuous sports or other exercises | 0.342 | 0.034 | 10.150 | 3.310E-24 |
| Intelligence | Smoking initiation | -0.185 | 0.018 | -10.144 | 3.510E-24 |
| Smoking cessation | Body fat percentage | 0.251 | 0.025 | 10.100 | 5.530E-24 |
| Intelligence | Strenuous sports or other exercises | 0.249 | 0.025 | 10.095 | 5.840E-24 |
| Cigarette consumption | Age of initiation of regular smoking | -0.369 | 0.037 | -10.035 | 1.070E-23 |
| Leptin | Waist-to-hip ratio | 0.556 | 0.056 | 10.018 | 1.270E-23 |
| Lifetime smoking | Whole body fat-free mass | 0.158 | 0.016 | 9.919 | 3.440E-23 |
| Anorexia nervosa | Educational attainment | 0.239 | 0.024 | 9.906 | 3.910E-23 |
| Intelligence | Schizophrenia | -0.203 | 0.021 | -9.845 | 7.210E-23 |
| Schizophrenia | Intelligence | -0.203 | 0.021 | -9.845 | 7.210E-23 |
| Intelligence | Caffeine consumption | 0.229 | 0.023 | 9.844 | 7.240E-23 |
| Anorexia nervosa | Life satisfaction | -0.289 | 0.029 | -9.836 | 7.850E-23 |
| HDL cholesterol | Waist circumference | -0.396 | 0.040 | -9.813 | 9.860E-23 |
| Lifetime smoking | Long sleep | 0.285 | 0.029 | 9.797 | 1.160E-22 |
| Alcohol consumption | Caffeine consumption | 0.267 | 0.027 | 9.786 | 1.290E-22 |
| Intelligence | Anxious feeling | -0.227 | 0.023 | -9.785 | 1.310E-22 |
| Breakfast skipping | Body fat percentage | 0.273 | 0.028 | 9.781 | 1.350E-22 |
| Anorexia nervosa | Leisure sedentary behaviour (TV watching) | -0.287 | 0.030 | -9.707 | 2.810E-22 |
| Atrial fibrillation | Visceral adipose tissue | 0.210 | 0.022 | 9.707 | 2.820E-22 |
| BMI | Atrial fibrillation | 0.203 | 0.021 | 9.641 | 5.350E-22 |
| Intelligence | Whole body fat mass | -0.149 | 0.016 | -9.625 | 6.290E-22 |
| BMI | Daytime napping | 0.154 | 0.016 | 9.617 | 6.760E-22 |
| Type 2 diabetes | Income | -0.214 | 0.022 | -9.591 | 8.700E-22 |
| Smoking cessation | Waist-to-hip ratio | 0.244 | 0.025 | 9.573 | 1.040E-21 |
| Daytime napping | Life satisfaction | -0.209 | 0.022 | -9.520 | 1.650E-21 |
| Major depressive disorder | Educational attainment | -0.173 | 0.018 | -9.525 | 1.650E-21 |
| Intelligence | Sitting height | 0.148 | 0.016 | 9.516 | 1.800E-21 |
| Age of initiation of regular smoking | Short sleep | -0.318 | 0.033 | -9.505 | 2.010E-21 |
| Short sleep | Body fat percentage | 0.206 | 0.022 | 9.498 | 2.140E-21 |
| Insomnia | Body fat percentage | 0.198 | 0.021 | 9.473 | 2.730E-21 |
| Strenuous sports or other exercises | Hip circumference | -0.190 | 0.020 | -9.426 | 4.270E-21 |
| Smoking cessation | Whole body fat mass | 0.238 | 0.025 | 9.425 | 4.300E-21 |
| Waist-to-hip ratio | Sitting height | -0.154 | 0.016 | -9.421 | 4.480E-21 |
| Age of initiation of regular smoking | Major depressive disorder | -0.261 | 0.028 | -9.403 | 5.300E-21 |
| Cigarette consumption | Visceral adipose tissue | 0.366 | 0.039 | 9.402 | 5.340E-21 |
| 10+ minutes vigorous activity | Whole body fat mass | -0.201 | 0.021 | -9.400 | 5.660E-21 |
| Coronary artery disease | HDL cholesterol | -0.297 | 0.032 | -9.391 | 5.960E-21 |
| Smoking initiation | Whole body fat-free mass | 0.145 | 0.016 | 9.383 | 6.400E-21 |

| | | | | | |
|---|---|---|---|---|---|
| Daytime sleepiness | Life satisfaction | -0.234 | 0.025 | -9.380 | 6.830E-21 |
| Schizophrenia | Lifetime smoking | 0.185 | 0.020 | 9.366 | 7.550E-21 |
| Age of initiation of regular smoking | Insomnia | -0.291 | 0.031 | -9.362 | 7.810E-21 |
| Long sleep | Body fat percentage | 0.223 | 0.024 | 9.345 | 9.160E-21 |
| BMI | Standing height | -0.142 | 0.015 | -9.337 | 9.930E-21 |
| Body fat percentage | Hand grip strength (left) | -0.156 | 0.017 | -9.309 | 1.290E-20 |
| Long sleep | Major depressive disorder | 0.302 | 0.033 | 9.295 | 1.470E-20 |
| Intelligence | Waist circumference | -0.141 | 0.015 | -9.239 | 2.480E-20 |
| Short sleep | Whole body fat mass | 0.201 | 0.022 | 9.197 | 3.690E-20 |
| Cigarette consumption | Whole body fat-free mass | 0.210 | 0.023 | 9.187 | 4.040E-20 |
| Daytime napping | Positive affect | -0.200 | 0.022 | -9.180 | 4.190E-20 |
| Strenuous sports or other exercises | Major depressive disorder | -0.253 | 0.028 | -9.178 | 4.380E-20 |
| BMI | HDL cholesterol | -0.359 | 0.039 | -9.113 | 7.990E-20 |
| Coronary artery disease | Hip circumference | 0.194 | 0.021 | 9.105 | 8.610E-20 |
| Caffeine consumption | Hip circumference | 0.220 | 0.024 | 9.071 | 1.180E-19 |
| Smoking cessation | Leisure sedentary behaviour (computer use) | -0.291 | 0.032 | -9.060 | 1.310E-19 |
| Smoking initiation | Insomnia | 0.195 | 0.022 | 9.053 | 1.400E-19 |
| Anorexia nervosa | Major depressive disorder | 0.287 | 0.032 | 9.049 | 1.440E-19 |
| Type 2 diabetes | Smoking initiation | 0.155 | 0.017 | 9.029 | 1.730E-19 |
| Caffeine consumption | Whole body fat-free mass | 0.244 | 0.027 | 9.008 | 2.100E-19 |
| Daytime sleepiness | Positive affect | -0.225 | 0.025 | -9.000 | 2.170E-19 |
| Coronary artery disease | Triglycerides | 0.288 | 0.032 | 8.997 | 2.320E-19 |
| Fasting insulin | Waist-to-hip ratio | 0.279 | 0.031 | 8.950 | 3.480E-19 |
| Type 2 diabetes | Strenuous sports or other exercises | -0.231 | 0.026 | -8.938 | 3.970E-19 |
| Strenuous sports or other exercises | Life satisfaction | 0.250 | 0.028 | 8.927 | 4.390E-19 |
| Anorexia nervosa | Anxious feeling | 0.331 | 0.037 | 8.921 | 4.620E-19 |
| Cigarette consumption | Daytime napping | 0.211 | 0.024 | 8.907 | 5.240E-19 |
| Insomnia | Waist-to-hip ratio | 0.189 | 0.021 | 8.881 | 6.630E-19 |
| Type 2 diabetes | Age of initiation of regular smoking | -0.233 | 0.026 | -8.876 | 6.920E-19 |
| Hip circumference | Anxious feeling | -0.174 | 0.020 | -8.874 | 7.050E-19 |
| Coronary artery disease | Short sleep | 0.207 | 0.023 | 8.865 | 7.690E-19 |
| Cigarette consumption | Strenuous sports or other exercises | -0.311 | 0.035 | -8.860 | 8.010E-19 |
| Insomnia | Visceral adipose tissue | 0.213 | 0.024 | 8.853 | 8.550E-19 |
| Daytime napping | Major depressive disorder | 0.195 | 0.022 | 8.820 | 1.120E-18 |
| Waist-to-hip ratio | Major depressive disorder | 0.159 | 0.018 | 8.812 | 1.230E-18 |
| BMI | Breakfast skipping | 0.267 | 0.030 | 8.810 | 1.260E-18 |
| Moderate to vigorous physical activity levels | Educational attainment | -0.210 | 0.024 | -8.770 | 1.750E-18 |
| Atrial fibrillation | Body fat percentage | 0.184 | 0.021 | 8.754 | 2.070E-18 |
| Strenuous sports or other exercises | Positive affect | 0.247 | 0.028 | 8.733 | 2.480E-18 |
| 25-Hydroxyvitamin D | Educational attainment | -0.208 | 0.024 | -8.732 | 2.510E-18 |
| Type 2 diabetes | Fasting glucose | 0.415 | 0.048 | 8.713 | 2.950E-18 |
| Coronary artery disease | Intelligence | -0.157 | 0.018 | -8.703 | 3.240E-18 |

| | | | | | |
|---|---|---|---|---|---|
| Body fat percentage | Hand grip strength (right) | -0.145 | 0.017 | -8.687 | 3.710E-18 |
| Breakfast skipping | Leisure sedentary behaviour (TV watching) | 0.272 | 0.032 | 8.650 | 5.140E-18 |
| Visceral adipose tissue | Major depressive disorder | 0.172 | 0.020 | 8.648 | 5.250E-18 |
| 10+ minutes vigorous activity | Visceral adipose tissue | -0.206 | 0.024 | -8.640 | 5.590E-18 |
| Daytime napping | Hip circumference | 0.138 | 0.016 | 8.630 | 6.140E-18 |
| Caffeine consumption | Intake of carbohydrate | -0.299 | 0.035 | -8.577 | 9.720E-18 |
| Intelligence | Short sleep | -0.182 | 0.021 | -8.572 | 1.020E-17 |
| Leisure sedentary behaviour (computer use) | Whole body fat-free mass | 0.147 | 0.017 | 8.550 | 1.190E-17 |
| Breakfast skipping | Visceral adipose tissue | 0.274 | 0.032 | 8.493 | 2.020E-17 |
| Waist-to-hip ratio | Childhood BMI | 0.278 | 0.033 | 8.484 | 2.170E-17 |
| Breakfast skipping | Waist-to-hip ratio | 0.241 | 0.028 | 8.464 | 2.590E-17 |
| Insomnia | Daytime sleepiness | 0.237 | 0.028 | 8.444 | 3.080E-17 |
| Caffeine consumption | Income | 0.261 | 0.031 | 8.439 | 3.210E-17 |
| Long sleep | Visceral adipose tissue | 0.248 | 0.029 | 8.418 | 3.820E-17 |
| Breakfast skipping | Whole body fat mass | 0.243 | 0.029 | 8.414 | 3.960E-17 |
| Smoking initiation | Cigarette consumption | 0.284 | 0.034 | 8.414 | 3.960E-17 |
| Breakfast skipping | Waist circumference | 0.253 | 0.030 | 8.368 | 5.860E-17 |
| Coronary artery disease | Standing height | -0.142 | 0.017 | -8.354 | 6.580E-17 |
| Coronary artery disease | Age of initiation of regular smoking | -0.248 | 0.030 | -8.335 | 7.730E-17 |
| BMI | Adult-onset asthma | 0.191 | 0.023 | 8.332 | 7.960E-17 |
| Adult-onset asthma | Major depressive disorder | 0.254 | 0.031 | 8.329 | 8.170E-17 |
| BMI | Caffeine consumption | 0.201 | 0.024 | 8.318 | 8.950E-17 |
| Triglycerides | Waist-to-hip ratio | 0.383 | 0.046 | 8.313 | 9.350E-17 |
| Long sleep | Strenuous sports or other exercises | -0.308 | 0.037 | -8.312 | 9.450E-17 |
| Breakfast skipping | Short sleep | 0.315 | 0.038 | 8.307 | 9.810E-17 |
| Long sleep | Waist-to-hip ratio | 0.227 | 0.027 | 8.285 | 1.190E-16 |
| 10+ minutes vigorous activity | Hip circumference | -0.187 | 0.023 | -8.270 | 1.360E-16 |
| Schizophrenia | Anorexia nervosa | 0.211 | 0.026 | 8.258 | 1.480E-16 |
| Leisure sedentary behaviour (computer use) | Sitting height | 0.143 | 0.017 | 8.250 | 1.520E-16 |
| 10+ minutes vigorous activity | Waist circumference | -0.192 | 0.023 | -8.250 | 1.580E-16 |
| Hip circumference | Income | -0.145 | 0.018 | -8.250 | 1.580E-16 |
| Caffeine consumption | Waist circumference | 0.194 | 0.024 | 8.200 | 2.410E-16 |
| Alcohol consumption | Coffee consumption | 0.223 | 0.027 | 8.188 | 2.650E-16 |
| HDL cholesterol | Triglycerides | -0.650 | 0.080 | -8.161 | 3.330E-16 |
| Cigarette consumption | Insomnia | 0.225 | 0.028 | 8.149 | 3.660E-16 |
| Coronary artery disease | Strenuous sports or other exercises | -0.197 | 0.024 | -8.134 | 4.160E-16 |
| Insomnia | Waist circumference | 0.189 | 0.023 | 8.124 | 4.500E-16 |
| Cigarette consumption | Breakfast skipping | 0.344 | 0.042 | 8.110 | 5.080E-16 |
| Bipolar disorder | Lifetime smoking | 0.172 | 0.021 | 8.073 | 6.870E-16 |
| Intake of fat | Breakfast skipping | 0.376 | 0.047 | 8.067 | 7.210E-16 |
| Waist circumference | Major depressive disorder | 0.143 | 0.018 | 8.058 | 7.780E-16 |

| | | | | | |
|---|---|---|---|---|---|
| HDL cholesterol | Whole body fat mass | -0.330 | 0.041 | -8.052 | 8.140E-16 |
| Age of initiation of regular smoking | Hip circumference | -0.193 | 0.024 | -8.048 | 8.430E-16 |
| Smoking cessation | Hip circumference | 0.195 | 0.024 | 8.036 | 9.290E-16 |
| Smoking initiation | Intake of total sugar | -0.239 | 0.030 | -8.016 | 1.090E-15 |
| Insomnia | Daytime napping | 0.199 | 0.025 | 8.015 | 1.100E-15 |
| Type 2 diabetes | Leptin | 0.503 | 0.063 | 7.967 | 1.630E-15 |
| Whole body fat mass | Birth weight | 0.134 | 0.017 | 7.965 | 1.660E-15 |
| Bipolar disorder | Anorexia nervosa | 0.229 | 0.029 | 7.954 | 1.800E-15 |
| Type 2 diabetes | 2-hour blood glucose | 0.413 | 0.052 | 7.954 | 1.810E-15 |
| Body fat percentage | Major depressive disorder | 0.130 | 0.016 | 7.950 | 1.860E-15 |
| Moderate to vigorous physical activity levels | Income | -0.228 | 0.029 | -7.860 | 3.740E-15 |
| Caffeine consumption | Whole body fat mass | 0.184 | 0.023 | 7.856 | 3.960E-15 |
| Intelligence | 10+ minutes vigorous activity | -0.214 | 0.027 | -7.847 | 4.270E-15 |
| Long sleep | Leisure sedentary behaviour (computer use) | -0.226 | 0.029 | -7.823 | 5.150E-15 |
| Breakfast skipping | Educational attainment | -0.214 | 0.027 | -7.794 | 6.490E-15 |
| Lifetime smoking | Daytime napping | 0.167 | 0.021 | 7.791 | 6.630E-15 |
| Long sleep | Whole body fat mass | 0.186 | 0.024 | 7.782 | 7.150E-15 |
| Intelligence | 25-Hydroxyvitamin D | -0.220 | 0.028 | -7.775 | 7.550E-15 |
| Cigarette consumption | Leisure sedentary behaviour (TV watching) | 0.228 | 0.029 | 7.773 | 7.680E-15 |
| HbA1c | Waist circumference | 0.190 | 0.025 | 7.691 | 1.460E-14 |
| Schizophrenia | Daytime napping | 0.158 | 0.021 | 7.682 | 1.570E-14 |
| Adult-onset asthma | Body fat percentage | 0.171 | 0.022 | 7.659 | 1.880E-14 |
| Life satisfaction | Educational attainment | 0.161 | 0.021 | 7.649 | 2.030E-14 |
| HbA1c | Waist-to-hip ratio | 0.199 | 0.026 | 7.641 | 2.150E-14 |
| Insomnia | Whole body fat mass | 0.169 | 0.022 | 7.587 | 3.290E-14 |
| BMI | Insomnia | 0.181 | 0.024 | 7.580 | 3.450E-14 |
| Adult-onset asthma | Waist circumference | 0.184 | 0.024 | 7.562 | 3.980E-14 |
| BMI | Anxious feeling | -0.149 | 0.020 | -7.561 | 4.010E-14 |
| Type 2 diabetes | Childhood BMI | 0.291 | 0.039 | 7.541 | 4.660E-14 |
| Bipolar disorder | Leisure sedentary behaviour (TV watching) | -0.155 | 0.021 | -7.534 | 4.940E-14 |
| Breakfast skipping | Strenuous sports or other exercises | -0.291 | 0.039 | -7.521 | 5.440E-14 |
| Leptin | Whole body fat-free mass | 0.398 | 0.053 | 7.513 | 5.780E-14 |
| Smoking cessation | Breakfast skipping | 0.366 | 0.049 | 7.506 | 6.090E-14 |
| Insomnia | Hand grip strength (left) | -0.167 | 0.022 | -7.491 | 6.830E-14 |
| Whole body fat-free mass | Anxious feeling | -0.144 | 0.019 | -7.472 | 7.900E-14 |
| HbA1c | Visceral adipose tissue | 0.204 | 0.027 | 7.445 | 9.670E-14 |
| Leisure sedentary behaviour (TV watching) | Fasting insulin | 0.188 | 0.025 | 7.420 | 1.130E-13 |
| Moderate to vigorous physical activity levels | Body fat percentage | -0.145 | 0.020 | -7.420 | 1.140E-13 |

| | | | | | |
|---|---|---|---|---|---|
| HDL cholesterol | Body fat percentage | -0.308 | 0.041 | -7.421 | 1.160E-13 |
| HDL cholesterol | Educational attainment | 0.202 | 0.027 | 7.399 | 1.370E-13 |
| Smoking cessation | Short sleep | 0.258 | 0.035 | 7.396 | 1.400E-13 |
| HDL cholesterol | Whole body fat-free mass | -0.222 | 0.030 | -7.385 | 1.530E-13 |
| Daytime sleepiness | Waist circumference | 0.151 | 0.021 | 7.370 | 1.690E-13 |
| Leisure sedentary behaviour (TV watching) | Standing height | -0.121 | 0.017 | -7.360 | 1.890E-13 |
| Intake of total sugar | Body fat percentage | -0.184 | 0.025 | -7.344 | 2.080E-13 |
| BMI | Major depressive disorder | 0.131 | 0.018 | 7.334 | 2.240E-13 |
| Leisure sedentary behaviour (computer use) | Standing height | 0.122 | 0.017 | 7.330 | 2.320E-13 |
| Coronary artery disease | Life satisfaction | -0.154 | 0.021 | -7.327 | 2.360E-13 |
| Caffeine consumption | Leisure sedentary behaviour (TV watching) | -0.183 | 0.025 | -7.325 | 2.400E-13 |
| Leisure sedentary behaviour (TV watching) | Triglycerides | 0.195 | 0.027 | 7.310 | 2.730E-13 |
| BMI | HbA1c | 0.178 | 0.024 | 7.296 | 2.970E-13 |
| Schizophrenia | Income | -0.156 | 0.022 | -7.261 | 3.850E-13 |
| Insomnia | Hand grip strength (right) | -0.157 | 0.022 | -7.249 | 4.210E-13 |
| Type 2 diabetes | Intelligence | -0.132 | 0.018 | -7.246 | 4.280E-13 |
| Coffee consumption | Waist-to-hip ratio | 0.166 | 0.023 | 7.246 | 4.290E-13 |
| Caffeine consumption | Smoking initiation | 0.172 | 0.024 | 7.245 | 4.320E-13 |
| Type 2 diabetes | Anorexia nervosa | -0.195 | 0.027 | -7.242 | 4.410E-13 |
| Triglycerides | Waist circumference | 0.285 | 0.039 | 7.238 | 4.560E-13 |
| Short sleep | Long sleep | -0.286 | 0.040 | -7.233 | 4.720E-13 |
| Smoking initiation | Strenuous sports or other exercises | -0.190 | 0.026 | -7.221 | 5.150E-13 |
| Coffee consumption | Intake of carbohydrate | -0.269 | 0.037 | -7.214 | 5.430E-13 |
| Short sleep | Hip circumference | 0.160 | 0.022 | 7.205 | 5.820E-13 |
| Cigarette consumption | Short sleep | 0.223 | 0.031 | 7.178 | 7.080E-13 |
| Adult-onset asthma | Waist-to-hip ratio | 0.168 | 0.024 | 7.124 | 1.050E-12 |
| Leisure sedentary behaviour (TV watching) | 10+ minutes vigorous activity | -0.199 | 0.028 | -7.100 | 1.230E-12 |
| Adult-onset asthma | Whole body fat mass | 0.165 | 0.023 | 7.097 | 1.280E-12 |
| Long sleep | Waist circumference | 0.176 | 0.025 | 7.083 | 1.410E-12 |
| Moderate to vigorous physical activity levels | Hand grip strength (left) | 0.157 | 0.022 | 7.080 | 1.460E-12 |
| Type 2 diabetes | Insomnia | 0.187 | 0.026 | 7.074 | 1.510E-12 |
| Anorexia nervosa | Whole body fat-free mass | -0.164 | 0.023 | -7.070 | 1.550E-12 |
| Age of initiation of regular smoking | Smoking cessation | -0.291 | 0.041 | -7.056 | 1.710E-12 |
| Type 2 diabetes | Smoking cessation | 0.199 | 0.028 | 7.036 | 1.980E-12 |
| Fasting insulin | Childhood BMI | -0.324 | 0.046 | -7.030 | 2.060E-12 |
| Fasting glucose | HbA1c | 0.410 | 0.058 | 7.030 | 2.070E-12 |
| Intake of carbohydrate | Body fat percentage | -0.182 | 0.026 | -7.028 | 2.100E-12 |
| Moderate to vigorous physical activity | 25-Hydroxyvitamin D | 0.256 | 0.037 | 7.020 | 2.290E-12 |

| | | | | | |
|---|---|---|---|---|---|
| levels | | | | | |
| Daytime sleepiness | Visceral adipose tissue | 0.156 | 0.022 | 7.010 | 2.450E-12 |
| BMI | Daytime sleepiness | 0.139 | 0.020 | 6.999 | 2.580E-12 |
| Type 2 diabetes | Short sleep | 0.182 | 0.026 | 6.997 | 2.620E-12 |
| Bipolar disorder | Smoking initiation | 0.151 | 0.022 | 6.984 | 2.870E-12 |
| Lifetime smoking | Intake of total sugar | -0.221 | 0.032 | -6.962 | 3.340E-12 |
| Smoking cessation | Insomnia | 0.228 | 0.033 | 6.952 | 3.610E-12 |
| Cigarette consumption | HDL cholesterol | -0.220 | 0.032 | -6.942 | 3.860E-12 |
| HbA1c | Whole body fat mass | 0.169 | 0.024 | 6.933 | 4.130E-12 |
| Breakfast skipping | Insomnia | 0.225 | 0.033 | 6.919 | 4.540E-12 |
| Intelligence | Daytime napping | -0.134 | 0.019 | -6.916 | 4.660E-12 |
| Smoking cessation | Major depressive disorder | 0.229 | 0.033 | 6.915 | 4.680E-12 |
| Age of initiation of regular smoking | Long sleep | -0.272 | 0.039 | -6.909 | 4.870E-12 |
| Cigarette consumption | Long sleep | 0.263 | 0.038 | 6.905 | 5.010E-12 |
| Strenuous sports or other exercises | 25-Hydroxyvitamin D | 0.222 | 0.032 | 6.904 | 5.060E-12 |
| Caffeine consumption | Leisure sedentary behaviour (computer use) | 0.192 | 0.028 | 6.895 | 5.390E-12 |
| 2-hour blood glucose | Whole body fat-free mass | -0.243 | 0.035 | -6.890 | 5.420E-12 |
| Daytime napping | Triglycerides | 0.176 | 0.026 | 6.880 | 5.790E-12 |
| 10+ minutes vigorous activity | 25-Hydroxyvitamin D | 0.296 | 0.043 | 6.860 | 6.930E-12 |
| Schizophrenia | Morning person | -0.136 | 0.020 | -6.843 | 7.760E-12 |
| Leisure sedentary behaviour (computer use) | 25-Hydroxyvitamin D | -0.193 | 0.028 | -6.830 | 8.460E-12 |
| Anorexia nervosa | Strenuous sports or other exercises | 0.231 | 0.034 | 6.822 | 9.000E-12 |
| BMI | Schizophrenia | -0.109 | 0.016 | -6.790 | 1.120E-11 |
| Coronary artery disease | Daytime napping | 0.137 | 0.020 | 6.785 | 1.160E-11 |
| Intake of carbohydrate | Whole body fat mass | -0.175 | 0.026 | -6.782 | 1.190E-11 |
| Long sleep | Life satisfaction | -0.207 | 0.031 | -6.781 | 1.200E-11 |
| Waist-to-hip ratio | Hand grip strength (right) | -0.120 | 0.018 | -6.773 | 1.260E-11 |
| Waist-to-hip ratio | Life satisfaction | -0.126 | 0.019 | -6.773 | 1.260E-11 |
| Fasting insulin | Educational attainment | -0.142 | 0.021 | -6.770 | 1.300E-11 |
| Cigarette consumption | Life satisfaction | -0.199 | 0.029 | -6.767 | 1.320E-11 |
| Schizophrenia | Smoking initiation | 0.143 | 0.021 | 6.736 | 1.630E-11 |
| Anxious feeling | Educational attainment | -0.146 | 0.022 | -6.733 | 1.660E-11 |
| Whole body fat mass | Anxious feeling | -0.138 | 0.021 | -6.732 | 1.670E-11 |
| Type 2 diabetes | Fasting insulin | 0.283 | 0.042 | 6.729 | 1.710E-11 |
| Morning person | 25-Hydroxyvitamin D | 0.148 | 0.022 | 6.724 | 1.770E-11 |
| Whole body fat mass | Major depressive disorder | 0.111 | 0.017 | 6.708 | 1.980E-11 |
| Cigarette consumption | 10+ minutes vigorous activity | -0.227 | 0.034 | -6.703 | 2.050E-11 |
| Alcohol consumption | Morning person | -0.137 | 0.021 | -6.689 | 2.250E-11 |
| Intelligence | Coffee consumption | 0.156 | 0.023 | 6.678 | 2.430E-11 |
| Adult-onset asthma | Visceral adipose tissue | 0.179 | 0.027 | 6.669 | 2.580E-11 |
| Daytime sleepiness | Major depressive disorder | 0.167 | 0.025 | 6.650 | 2.850E-11 |
| Intake of fat | Strenuous sports or other exercises | -0.251 | 0.038 | -6.654 | 2.850E-11 |

| | | | | | |
|---|---|---|---|---|---|
| Strenuous sports or other exercises | Fasting insulin | -0.220 | 0.033 | -6.651 | 2.920E-11 |
| Long sleep | Leisure sedentary behaviour (TV watching) | 0.218 | 0.033 | 6.648 | 2.970E-11 |
| Intake of carbohydrate | Waist-to-hip ratio | -0.175 | 0.026 | -6.637 | 3.210E-11 |
| Age of initiation of regular smoking | Life satisfaction | 0.190 | 0.029 | 6.630 | 3.360E-11 |
| Hip circumference | Hand grip strength (right) | 0.121 | 0.018 | 6.619 | 3.620E-11 |
| HDL cholesterol | Hip circumference | -0.274 | 0.042 | -6.604 | 4.010E-11 |
| Coronary artery disease | Daytime sleepiness | 0.146 | 0.022 | 6.584 | 4.570E-11 |
| Anxious feeling | Income | -0.182 | 0.028 | -6.554 | 5.590E-11 |
| Smoking cessation | Life satisfaction | -0.220 | 0.034 | -6.554 | 5.610E-11 |
| Coronary artery disease | Positive affect | -0.143 | 0.022 | -6.543 | 6.020E-11 |
| Waist-to-hip ratio | Hand grip strength (left) | -0.116 | 0.018 | -6.527 | 6.710E-11 |
| Intake of total sugar | Whole body fat mass | -0.166 | 0.025 | -6.525 | 6.780E-11 |
| BMI | Long sleep | 0.169 | 0.026 | 6.519 | 7.100E-11 |
| Type 2 diabetes | Major depressive disorder | 0.139 | 0.021 | 6.513 | 7.360E-11 |
| Positive affect | Educational attainment | 0.140 | 0.022 | 6.505 | 7.750E-11 |
| Intake of carbohydrate | Waist circumference | -0.173 | 0.027 | -6.492 | 8.500E-11 |
| Leisure sedentary behaviour (TV watching) | HDL cholesterol | -0.199 | 0.031 | -6.490 | 8.640E-11 |
| Adult-onset asthma | Insomnia | 0.195 | 0.030 | 6.485 | 8.890E-11 |
| Cigarette consumption | Positive affect | -0.193 | 0.030 | -6.484 | 8.940E-11 |
| Type 2 diabetes | Triglycerides | 0.351 | 0.054 | 6.475 | 9.490E-11 |
| Lifetime smoking | HDL cholesterol | -0.166 | 0.026 | -6.472 | 9.650E-11 |
| Bipolar disorder | Long sleep | 0.226 | 0.035 | 6.470 | 9.780E-11 |
| Schizophrenia | Hip circumference | -0.112 | 0.017 | -6.469 | 9.840E-11 |
| Hand grip strength (right) | Income | 0.144 | 0.022 | 6.461 | 1.040E-10 |
| Coronary artery disease | Cigarette consumption | 0.294 | 0.046 | 6.455 | 1.080E-10 |
| Cigarette consumption | Triglycerides | 0.193 | 0.030 | 6.454 | 1.090E-10 |
| Daytime napping | Income | -0.153 | 0.024 | -6.450 | 1.110E-10 |
| Waist circumference | Birth weight | 0.110 | 0.017 | 6.433 | 1.250E-10 |
| Bipolar disorder | Educational attainment | 0.112 | 0.018 | 6.433 | 1.260E-10 |
| Long sleep | Positive affect | -0.202 | 0.032 | -6.406 | 1.500E-10 |
| HbA1c | Body fat percentage | 0.158 | 0.025 | 6.400 | 1.560E-10 |
| Moderate to vigorous physical activity levels | Whole body fat mass | -0.133 | 0.021 | -6.390 | 1.700E-10 |
| Coronary artery disease | Smoking cessation | 0.187 | 0.029 | 6.365 | 1.950E-10 |
| Intelligence | Insomnia | -0.131 | 0.021 | -6.363 | 1.980E-10 |
| Coronary artery disease | Birth weight | -0.160 | 0.025 | -6.360 | 2.010E-10 |
| Triglycerides | Body fat percentage | 0.231 | 0.036 | 6.350 | 2.160E-10 |
| BMI | Triglycerides | 0.246 | 0.039 | 6.342 | 2.270E-10 |
| Intake of fat | Leisure sedentary behaviour (TV watching) | 0.194 | 0.031 | 6.336 | 2.360E-10 |
| Coffee consumption | Educational attainment | 0.120 | 0.019 | 6.318 | 2.660E-10 |
| Hand grip strength (right) | Life satisfaction | 0.144 | 0.023 | 6.295 | 3.070E-10 |

| | | | | | |
|---|---|---|---|---|---|
| Triglycerides | Visceral adipose tissue | 0.293 | 0.047 | 6.279 | 3.410E-10 |
| Daytime sleepiness | Hip circumference | 0.129 | 0.021 | 6.270 | 3.710E-10 |
| Waist-to-hip ratio | Positive affect | -0.116 | 0.019 | -6.247 | 4.180E-10 |
| Schizophrenia | Whole body fat-free mass | -0.097 | 0.016 | -6.239 | 4.400E-10 |
| Type 2 diabetes | Long sleep | 0.214 | 0.034 | 6.239 | 4.420E-10 |
| Fasting insulin | HDL cholesterol | -0.316 | 0.051 | -6.240 | 4.500E-10 |
| Intake of total sugar | Waist circumference | -0.158 | 0.025 | -6.226 | 4.780E-10 |
| Hand grip strength (left) | Life satisfaction | 0.138 | 0.022 | 6.210 | 5.310E-10 |
| Caffeine consumption | Long sleep | -0.238 | 0.039 | -6.187 | 6.110E-10 |
| Smoking cessation | Positive affect | -0.211 | 0.034 | -6.188 | 6.110E-10 |
| Age of initiation of regular smoking | Positive affect | 0.176 | 0.029 | 6.187 | 6.120E-10 |
| Leisure sedentary behaviour (TV watching) | Life satisfaction | -0.141 | 0.023 | -6.180 | 6.410E-10 |
| Schizophrenia | Waist circumference | -0.097 | 0.016 | -6.171 | 6.800E-10 |
| Coffee consumption | Lifetime smoking | 0.179 | 0.029 | 6.158 | 7.370E-10 |
| 25-Hydroxyvitamin D | Whole body fat-free mass | 0.091 | 0.015 | 6.152 | 7.660E-10 |
| Lifetime smoking | HbA1c | 0.152 | 0.025 | 6.145 | 7.980E-10 |
| Anorexia nervosa | 10+ minutes vigorous activity | 0.236 | 0.038 | 6.142 | 8.130E-10 |
| Coffee consumption | Childhood BMI | 0.285 | 0.046 | 6.139 | 8.310E-10 |
| Birth weight | Childhood BMI | 0.212 | 0.035 | 6.125 | 9.050E-10 |
| Cigarette consumption | Intake of carbohydrate | -0.207 | 0.034 | -6.108 | 1.010E-09 |
| Hand grip strength (right) | Educational attainment | 0.100 | 0.016 | 6.100 | 1.060E-09 |
| Hand grip strength (right) | Positive affect | 0.141 | 0.023 | 6.100 | 1.060E-09 |
| Schizophrenia | Whole body fat mass | -0.098 | 0.016 | -6.095 | 1.100E-09 |
| Birth weight | Educational attainment | 0.113 | 0.019 | 6.083 | 1.180E-09 |
| Intake of carbohydrate | Visceral adipose tissue | -0.174 | 0.029 | -6.074 | 1.250E-09 |
| Moderate to vigorous physical activity levels | Hip circumference | -0.133 | 0.022 | -6.070 | 1.290E-09 |
| Coronary artery disease | HbA1c | 0.182 | 0.030 | 6.064 | 1.330E-09 |
| Adult-onset asthma | Hip circumference | 0.146 | 0.024 | 6.040 | 1.540E-09 |
| Hand grip strength (left) | Positive affect | 0.135 | 0.022 | 6.028 | 1.660E-09 |
| Daytime napping | Strenuous sports or other exercises | -0.144 | 0.024 | -6.020 | 1.780E-09 |
| Daytime sleepiness | Whole body fat mass | 0.124 | 0.021 | 6.020 | 1.780E-09 |
| Type 2 diabetes | Daytime sleepiness | 0.123 | 0.021 | 5.999 | 1.990E-09 |
| Coffee consumption | Smoking initiation | 0.150 | 0.025 | 5.994 | 2.040E-09 |
| Leisure sedentary behaviour (computer use) | Hip circumference | 0.109 | 0.018 | 5.990 | 2.040E-09 |
| Hip circumference | Hand grip strength (left) | 0.103 | 0.017 | 5.983 | 2.190E-09 |
| Leisure sedentary behaviour (TV watching) | Whole body fat-free mass | 0.111 | 0.019 | 5.980 | 2.210E-09 |
| Lifetime smoking | Standing height | -0.082 | 0.014 | -5.981 | 2.220E-09 |
| Daytime sleepiness | Waist-to-hip ratio | 0.119 | 0.020 | 5.970 | 2.380E-09 |
| Triglycerides | Whole body fat mass | 0.219 | 0.037 | 5.955 | 2.600E-09 |
| Caffeine consumption | Intake of total sugar | -0.235 | 0.040 | -5.954 | 2.620E-09 |

| | | | | | |
|---|---|---|---|---|---|
| Alcohol consumption | HDL cholesterol | 0.195 | 0.033 | 5.951 | 2.670E-09 |
| Smoking cessation | Long sleep | 0.260 | 0.044 | 5.905 | 3.540E-09 |
| Lifetime smoking | Triglycerides | 0.158 | 0.027 | 5.890 | 3.870E-09 |
| Adult-onset asthma | Type 2 diabetes | 0.160 | 0.027 | 5.831 | 5.500E-09 |
| Bipolar disorder | Daytime napping | 0.133 | 0.023 | 5.817 | 6.000E-09 |
| Short sleep | Standing height | -0.101 | 0.017 | -5.810 | 6.260E-09 |
| Coronary artery disease | Long sleep | 0.175 | 0.030 | 5.804 | 6.490E-09 |
| Smoking initiation | Daytime napping | 0.123 | 0.021 | 5.784 | 7.280E-09 |
| Intake of total sugar | Moderate to vigorous physical activity levels | 0.233 | 0.040 | 5.769 | 7.990E-09 |
| Daytime sleepiness | Body fat percentage | 0.117 | 0.020 | 5.760 | 8.340E-09 |
| Caffeine consumption | Lifetime smoking | 0.162 | 0.028 | 5.752 | 8.810E-09 |
| 2-hour blood glucose | HbA1c | 0.311 | 0.054 | 5.750 | 8.890E-09 |
| Triglycerides | Educational attainment | -0.169 | 0.029 | -5.740 | 9.470E-09 |
| Breakfast skipping | Income | -0.195 | 0.034 | -5.731 | 9.990E-09 |
| Morning person | Moderate to vigorous physical activity levels | 0.134 | 0.023 | 5.727 | 1.020E-08 |
| Hand grip strength (right) | Major depressive disorder | -0.117 | 0.020 | -5.722 | 1.050E-08 |
| Age of initiation of regular smoking | HDL cholesterol | 0.218 | 0.038 | 5.719 | 1.070E-08 |
| Leisure sedentary behaviour (TV watching) | Positive affect | -0.132 | 0.023 | -5.700 | 1.200E-08 |
| Age of initiation of regular smoking | Breakfast skipping | -0.261 | 0.046 | -5.683 | 1.320E-08 |
| Short sleep | Anxious feeling | 0.159 | 0.028 | 5.681 | 1.340E-08 |
| Intake of total sugar | Waist-to-hip ratio | -0.147 | 0.026 | -5.669 | 1.440E-08 |
| BMI | Intake of carbohydrate | -0.149 | 0.026 | -5.665 | 1.470E-08 |
| Morning person | Educational attainment | -0.093 | 0.017 | -5.653 | 1.580E-08 |
| BMI | 10+ minutes vigorous activity | -0.129 | 0.023 | -5.647 | 1.630E-08 |
| Breakfast skipping | Hip circumference | 0.166 | 0.029 | 5.633 | 1.770E-08 |
| Adult-onset asthma | Life satisfaction | -0.156 | 0.028 | -5.623 | 1.880E-08 |
| Short sleep | HbA1c | 0.148 | 0.026 | 5.618 | 1.930E-08 |
| Leisure sedentary behaviour (TV watching) | Leptin | 0.271 | 0.048 | 5.600 | 2.150E-08 |
| Coronary artery disease | Hand grip strength (left) | -0.115 | 0.021 | -5.597 | 2.180E-08 |
| Waist circumference | Life satisfaction | -0.104 | 0.019 | -5.590 | 2.270E-08 |
| Intake of total sugar | Visceral adipose tissue | -0.157 | 0.028 | -5.585 | 2.340E-08 |
| Fasting insulin | Birth weight | -0.203 | 0.036 | -5.580 | 2.350E-08 |
| Insomnia | Hip circumference | 0.125 | 0.023 | 5.584 | 2.360E-08 |
| Daytime napping | Leisure sedentary behaviour (TV watching) | 0.115 | 0.021 | 5.570 | 2.490E-08 |
| Visceral adipose tissue | Life satisfaction | -0.112 | 0.020 | -5.566 | 2.600E-08 |
| 10+ minutes vigorous activity | Waist-to-hip ratio | -0.129 | 0.023 | -5.560 | 2.660E-08 |
| Daytime sleepiness | Anxious feeling | 0.155 | 0.028 | 5.550 | 2.820E-08 |
| Type 2 diabetes | Birth weight | -0.168 | 0.030 | -5.538 | 3.070E-08 |
| Smoking cessation | Daytime napping | 0.176 | 0.032 | 5.536 | 3.100E-08 |

| | | | | | |
|---|---|---|---|---|---|
| Triglycerides | 25-Hydroxyvitamin D | -0.210 | 0.038 | -5.490 | 4.020E-08 |
| Bipolar disorder | Alcohol consumption | 0.135 | 0.025 | 5.487 | 4.100E-08 |
| Triglycerides | Income | -0.180 | 0.033 | -5.480 | 4.260E-08 |
| Strenuous sports or other exercises | HDL cholesterol | 0.182 | 0.033 | 5.460 | 4.770E-08 |
| Fasting glucose | Childhood BMI | -0.219 | 0.040 | -5.455 | 4.900E-08 |
| Anorexia nervosa | HDL cholesterol | 0.199 | 0.036 | 5.454 | 4.920E-08 |
| Waist circumference | Anxious feeling | -0.117 | 0.021 | -5.454 | 4.920E-08 |
| BMI | 25-Hydroxyvitamin D | 0.088 | 0.016 | 5.438 | 5.370E-08 |
| Intake of fat | 10+ minutes vigorous activity | -0.234 | 0.043 | -5.434 | 5.500E-08 |
| Anorexia nervosa | Intake of carbohydrate | 0.200 | 0.037 | 5.430 | 5.620E-08 |
| Childhood-onset asthma | Educational attainment | 0.118 | 0.022 | 5.423 | 5.870E-08 |
| Smoking cessation | Standing height | -0.113 | 0.021 | -5.421 | 5.930E-08 |
| Leisure sedentary behaviour (computer use) | Strenuous sports or other exercises | 0.139 | 0.026 | 5.420 | 5.980E-08 |
| Schizophrenia | 25-Hydroxyvitamin D | -0.119 | 0.022 | -5.419 | 6.000E-08 |
| Age of initiation of regular smoking | Daytime napping | -0.157 | 0.029 | -5.407 | 6.410E-08 |
| 2-hour blood glucose | Hip circumference | -0.198 | 0.037 | -5.390 | 7.200E-08 |
| 10+ minutes vigorous activity | Hand grip strength (left) | 0.133 | 0.025 | 5.380 | 7.290E-08 |
| Coffee consumption | Cigarette consumption | 0.198 | 0.037 | 5.356 | 8.520E-08 |
| Type 2 diabetes | Breakfast skipping | 0.181 | 0.034 | 5.348 | 8.920E-08 |
| Atrial fibrillation | Waist-to-hip ratio | 0.108 | 0.020 | 5.335 | 9.530E-08 |
| 2-hour blood glucose | Birth weight | -0.242 | 0.046 | -5.310 | 1.100E-07 |
| Breakfast skipping | Life satisfaction | -0.177 | 0.033 | -5.298 | 1.170E-07 |
| Intelligence | Birth weight | 0.111 | 0.021 | 5.284 | 1.260E-07 |
| Long sleep | Anxious feeling | 0.181 | 0.034 | 5.283 | 1.270E-07 |
| HbA1c | Hip circumference | 0.131 | 0.025 | 5.281 | 1.290E-07 |
| Daytime napping | Educational attainment | -0.109 | 0.021 | -5.270 | 1.350E-07 |
| Smoking initiation | Morning person | -0.102 | 0.019 | -5.266 | 1.400E-07 |
| Moderate to vigorous physical activity levels | Waist circumference | -0.116 | 0.022 | -5.260 | 1.410E-07 |
| Insomnia | Leisure sedentary behaviour (computer use) | -0.116 | 0.022 | -5.254 | 1.490E-07 |
| Caffeine consumption | Body fat percentage | 0.123 | 0.023 | 5.253 | 1.500E-07 |
| Age of initiation of regular smoking | Standing height | 0.102 | 0.020 | 5.251 | 1.520E-07 |
| Intelligence | Major depressive disorder | -0.101 | 0.019 | -5.243 | 1.580E-07 |
| Insomnia | 10+ minutes vigorous activity | -0.158 | 0.030 | -5.242 | 1.590E-07 |
| Coffee consumption | Leisure sedentary behaviour (computer use) | 0.146 | 0.028 | 5.232 | 1.670E-07 |
| Short sleep | Daytime sleepiness | 0.150 | 0.029 | 5.233 | 1.670E-07 |
| Coffee consumption | Income | 0.158 | 0.030 | 5.227 | 1.720E-07 |
| Body fat percentage | Anxious feeling | -0.109 | 0.021 | -5.214 | 1.840E-07 |
| Schizophrenia | Leisure sedentary behaviour (TV watching) | -0.112 | 0.021 | -5.210 | 1.890E-07 |
| Fasting insulin | 2-hour blood glucose | 0.303 | 0.058 | 5.200 | 2.000E-07 |

| | | | | | |
|---|---|---|---|---|---|
| Adult-onset asthma | Positive affect | -0.144 | 0.028 | -5.182 | 2.200E-07 |
| Hand grip strength (right) | Anxious feeling | -0.107 | 0.021 | -5.169 | 2.350E-07 |
| Caffeine consumption | Anxious feeling | -0.160 | 0.031 | -5.163 | 2.430E-07 |
| Coronary artery disease | Hand grip strength (right) | -0.109 | 0.021 | -5.153 | 2.570E-07 |
| Intelligence | Morning person | -0.094 | 0.018 | -5.146 | 2.660E-07 |
| Leptin | Educational attainment | -0.219 | 0.043 | -5.146 | 2.670E-07 |
| Alcohol consumption | Age of initiation of regular smoking | -0.160 | 0.031 | -5.142 | 2.720E-07 |
| Daytime napping | Anxious feeling | 0.136 | 0.026 | 5.140 | 2.760E-07 |
| Short sleep | Hand grip strength (right) | -0.113 | 0.022 | -5.139 | 2.760E-07 |
| Age of initiation of regular smoking | Leisure sedentary behaviour (computer use) | 0.169 | 0.033 | 5.134 | 2.840E-07 |
| Breakfast skipping | Major depressive disorder | 0.179 | 0.035 | 5.133 | 2.850E-07 |
| Moderate to vigorous physical activity levels | Visceral adipose tissue | -0.114 | 0.022 | -5.110 | 3.270E-07 |
| Smoking cessation | Intake of carbohydrate | -0.233 | 0.046 | -5.103 | 3.340E-07 |
| Smoking initiation | HDL cholesterol | -0.130 | 0.026 | -5.084 | 3.700E-07 |
| HDL cholesterol | Income | 0.161 | 0.032 | 5.081 | 3.760E-07 |
| Waist-to-hip ratio | Standing height | -0.081 | 0.016 | -5.068 | 4.020E-07 |
| Caffeine consumption | Visceral adipose tissue | 0.136 | 0.027 | 5.055 | 4.300E-07 |
| Intake of carbohydrate | Morning person | 0.147 | 0.029 | 5.047 | 4.500E-07 |
| Leisure sedentary behaviour (computer use) | Anxious feeling | -0.134 | 0.027 | -5.040 | 4.560E-07 |
| Cigarette consumption | HbA1c | 0.148 | 0.029 | 5.033 | 4.840E-07 |
| Intake of total sugar | 10+ minutes vigorous activity | 0.200 | 0.040 | 5.031 | 4.890E-07 |
| Insomnia | Standing height | -0.088 | 0.018 | -5.020 | 5.160E-07 |
| Hand grip strength (left) | Income | 0.109 | 0.022 | 5.019 | 5.180E-07 |
| Coffee consumption | Long sleep | -0.199 | 0.040 | -5.011 | 5.430E-07 |
| Daytime napping | Whole body fat-free mass | 0.076 | 0.015 | 5.010 | 5.440E-07 |
| Anorexia nervosa | Moderate to vigorous physical activity levels | 0.176 | 0.035 | 4.991 | 6.000E-07 |
| Triglycerides | Major depressive disorder | 0.130 | 0.026 | 4.974 | 6.570E-07 |
| Waist circumference | Positive affect | -0.093 | 0.019 | -4.955 | 7.250E-07 |
| Intelligence | Cigarette consumption | -0.114 | 0.023 | -4.952 | 7.350E-07 |
| Body fat percentage | Life satisfaction | -0.089 | 0.018 | -4.949 | 7.470E-07 |
| Cigarette consumption | Intake of total sugar | -0.153 | 0.031 | -4.908 | 9.200E-07 |
| Hand grip strength (left) | Educational attainment | 0.081 | 0.017 | 4.897 | 9.710E-07 |
| HbA1c | Educational attainment | -0.106 | 0.022 | -4.891 | 1.000E-06 |
| Short sleep | Sitting height | -0.084 | 0.017 | -4.890 | 1.010E-06 |
| Standing height | Major depressive disorder | -0.068 | 0.014 | -4.882 | 1.050E-06 |
| Coffee consumption | Intake of total sugar | -0.206 | 0.042 | -4.875 | 1.090E-06 |
| Adult-onset asthma | Anxious feeling | 0.163 | 0.033 | 4.865 | 1.150E-06 |
| Breakfast skipping | Positive affect | -0.164 | 0.034 | -4.853 | 1.220E-06 |
| Bipolar disorder | Daytime sleepiness | 0.119 | 0.025 | 4.848 | 1.250E-06 |
| Caffeine consumption | Birth weight | 0.125 | 0.026 | 4.821 | 1.430E-06 |

| | | | | | |
|---|---|---|---|---|---|
| Strenuous sports or other exercises | Triglycerides | -0.157 | 0.033 | -4.821 | 1.430E-06 |
| Age of initiation of regular smoking | 25-Hydroxyvitamin D | -0.129 | 0.027 | -4.810 | 1.510E-06 |
| Smoking initiation | Triglycerides | 0.124 | 0.026 | 4.803 | 1.570E-06 |
| Visceral adipose tissue | Positive affect | -0.100 | 0.021 | -4.795 | 1.620E-06 |
| Coronary artery disease | Fasting insulin | 0.157 | 0.033 | 4.782 | 1.740E-06 |
| Schizophrenia | Visceral adipose tissue | -0.085 | 0.018 | -4.781 | 1.740E-06 |
| Caffeine consumption | 2-hour blood glucose | -0.244 | 0.051 | -4.780 | 1.760E-06 |
| Anorexia nervosa | Daytime napping | -0.157 | 0.033 | -4.778 | 1.770E-06 |
| HbA1c | Whole body fat-free mass | 0.111 | 0.023 | 4.768 | 1.860E-06 |
| Smoking initiation | Long sleep | 0.135 | 0.028 | 4.740 | 2.140E-06 |
| BMI | Intake of total sugar | -0.131 | 0.028 | -4.736 | 2.180E-06 |
| Daytime napping | HDL cholesterol | -0.134 | 0.028 | -4.730 | 2.270E-06 |
| Alcohol consumption | Strenuous sports or other exercises | 0.127 | 0.027 | 4.714 | 2.430E-06 |
| Breakfast skipping | Long sleep | 0.221 | 0.047 | 4.698 | 2.620E-06 |
| Leisure sedentary behaviour (TV watching) | Sitting height | -0.075 | 0.016 | -4.690 | 2.760E-06 |
| Schizophrenia | Moderate to vigorous physical activity levels | 0.127 | 0.027 | 4.688 | 2.760E-06 |
| 25-Hydroxyvitamin D | Major depressive disorder | -0.092 | 0.020 | -4.686 | 2.790E-06 |
| Bipolar disorder | Cigarette consumption | 0.123 | 0.026 | 4.684 | 2.810E-06 |
| Schizophrenia | Body fat percentage | -0.078 | 0.017 | -4.683 | 2.830E-06 |
| Moderate to vigorous physical activity levels | Sitting height | -0.094 | 0.020 | -4.680 | 2.840E-06 |
| Adult-onset asthma | Smoking initiation | 0.110 | 0.024 | 4.675 | 2.930E-06 |
| Adult-onset asthma | Leisure sedentary behaviour (TV watching) | 0.125 | 0.027 | 4.673 | 2.970E-06 |
| 25-Hydroxyvitamin D | Positive affect | 0.092 | 0.020 | 4.668 | 3.040E-06 |
| Morning person | Positive affect | 0.103 | 0.022 | 4.667 | 3.050E-06 |
| Type 2 diabetes | 10+ minutes vigorous activity | -0.122 | 0.026 | -4.657 | 3.210E-06 |
| Strenuous sports or other exercises | Hand grip strength (left) | 0.109 | 0.023 | 4.644 | 3.410E-06 |
| Intake of carbohydrate | Hip circumference | -0.118 | 0.025 | -4.642 | 3.460E-06 |
| LDL cholesterol | Triglycerides | 0.380 | 0.082 | 4.620 | 3.840E-06 |
| Morning person | 10+ minutes vigorous activity | 0.127 | 0.028 | 4.612 | 3.980E-06 |
| Breakfast skipping | Standing height | -0.123 | 0.027 | -4.610 | 4.040E-06 |
| Caffeine consumption | Strenuous sports or other exercises | 0.157 | 0.034 | 4.608 | 4.060E-06 |
| Anorexia nervosa | Age of initiation of regular smoking | 0.174 | 0.038 | 4.607 | 4.080E-06 |
| Lifetime smoking | Morning person | -0.105 | 0.023 | -4.607 | 4.090E-06 |
| Morning person | Daytime napping | 0.104 | 0.023 | 4.591 | 4.410E-06 |
| Hip circumference | Major depressive disorder | 0.077 | 0.017 | 4.582 | 4.610E-06 |
| Hand grip strength (left) | Major depressive disorder | -0.095 | 0.021 | -4.577 | 4.730E-06 |
| Moderate to vigorous physical activity levels | Anxious feeling | 0.154 | 0.034 | 4.570 | 4.790E-06 |
| Moderate to vigorous physical activity levels | Hand grip strength (right) | 0.102 | 0.022 | 4.570 | 4.850E-06 |

| | | | | | |
|---|---|---|---|---|---|
| Ulcerative colitis | Anxious feeling | 0.191 | 0.042 | 4.571 | 4.860E-06 |
| Anorexia nervosa | Intake of fat | -0.196 | 0.043 | -4.568 | 4.930E-06 |
| Schizophrenia | Cigarette consumption | 0.111 | 0.024 | 4.567 | 4.940E-06 |
| Intake of total sugar | Morning person | 0.146 | 0.032 | 4.567 | 4.950E-06 |
| Atrial fibrillation | Lifetime smoking | 0.095 | 0.021 | 4.564 | 5.020E-06 |
| Cigarette consumption | Childhood BMI | 0.173 | 0.038 | 4.561 | 5.100E-06 |
| 2-hour blood glucose | Standing height | -0.146 | 0.032 | -4.560 | 5.110E-06 |
| Leisure sedentary behaviour (TV watching) | Birth weight | -0.106 | 0.023 | -4.550 | 5.280E-06 |
| Coronary artery disease | Leptin | 0.221 | 0.049 | 4.549 | 5.380E-06 |
| Intelligence | Life satisfaction | 0.104 | 0.023 | 4.546 | 5.460E-06 |
| Short sleep | Hand grip strength (left) | -0.104 | 0.023 | -4.512 | 6.410E-06 |
| Anorexia nervosa | Fasting insulin | -0.172 | 0.038 | -4.509 | 6.520E-06 |
| Anorexia nervosa | Income | 0.134 | 0.030 | 4.508 | 6.550E-06 |
| Smoking initiation | 2-hour blood glucose | -0.164 | 0.037 | -4.503 | 6.710E-06 |
| Morning person | Life satisfaction | 0.098 | 0.022 | 4.502 | 6.730E-06 |
| Type 2 diabetes | Life satisfaction | -0.109 | 0.024 | -4.496 | 6.940E-06 |
| Short sleep | Whole body fat-free mass | 0.090 | 0.020 | 4.485 | 7.280E-06 |
| Leptin | Income | -0.245 | 0.055 | -4.483 | 7.350E-06 |
| Type 2 diabetes | Atrial fibrillation | 0.106 | 0.024 | 4.475 | 7.630E-06 |
| Coronary artery disease | Whole body fat-free mass | 0.083 | 0.019 | 4.468 | 7.890E-06 |
| Birth weight | Income | 0.107 | 0.024 | 4.461 | 8.160E-06 |
| 25-Hydroxyvitamin D | Hip circumference | 0.077 | 0.017 | 4.431 | 9.390E-06 |
| Alcohol consumption | Insomnia | 0.100 | 0.023 | 4.426 | 9.620E-06 |
| Lifetime smoking | Leptin | 0.215 | 0.049 | 4.415 | 1.008E-05 |
| Coronary artery disease | 2-hour blood glucose | 0.175 | 0.040 | 4.411 | 1.030E-05 |
| Fasting glucose | 2-hour blood glucose | 0.303 | 0.069 | 4.402 | 1.070E-05 |
| Daytime sleepiness | Leisure sedentary behaviour (TV watching) | 0.115 | 0.026 | 4.400 | 1.090E-05 |
| Intake of total sugar | Hip circumference | -0.112 | 0.025 | -4.397 | 1.096E-05 |
| Coronary artery disease | Breakfast skipping | 0.145 | 0.033 | 4.394 | 1.110E-05 |
| Long sleep | Daytime sleepiness | 0.143 | 0.033 | 4.385 | 1.160E-05 |
| 25-Hydroxyvitamin D | Life satisfaction | 0.085 | 0.019 | 4.376 | 1.210E-05 |
| Leisure sedentary behaviour (TV watching) | 25-Hydroxyvitamin D | 0.090 | 0.020 | 4.380 | 1.210E-05 |
| Intelligence | Intake of total sugar | -0.120 | 0.028 | -4.368 | 1.260E-05 |
| Atrial fibrillation | Major depressive disorder | 0.111 | 0.026 | 4.366 | 1.270E-05 |
| Fasting glucose | Birth weight | -0.145 | 0.033 | -4.350 | 1.360E-05 |
| Fasting glucose | Fasting insulin | 0.252 | 0.058 | 4.342 | 1.410E-05 |
| Cigarette consumption | Leptin | 0.301 | 0.069 | 4.340 | 1.424E-05 |
| Age of initiation of regular smoking | Intake of carbohydrate | 0.185 | 0.043 | 4.338 | 1.440E-05 |
| Body fat percentage | Positive affect | -0.078 | 0.018 | -4.326 | 1.520E-05 |
| BMI | Life satisfaction | -0.086 | 0.020 | -4.323 | 1.540E-05 |
| Age of initiation of regular smoking | Triglycerides | -0.165 | 0.038 | -4.318 | 1.580E-05 |

| | | | | | |
|---|---|---|---|---|---|
| Total cholesterol | Standing height | -0.101 | 0.023 | -4.315 | 1.600E-05 |
| Childhood-onset asthma | Intelligence | 0.099 | 0.023 | 4.305 | 1.670E-05 |
| Coffee consumption | Birth weight | 0.109 | 0.025 | 4.305 | 1.670E-05 |
| Childhood-onset asthma | Leisure sedentary behaviour (computer use) | 0.106 | 0.025 | 4.303 | 1.690E-05 |
| Age of initiation of regular smoking | Whole body fat-free mass | -0.102 | 0.024 | -4.300 | 1.710E-05 |
| Daytime napping | Leptin | 0.226 | 0.053 | 4.300 | 1.730E-05 |
| Schizophrenia | Hand grip strength (right) | -0.082 | 0.019 | -4.278 | 1.890E-05 |
| Ulcerative colitis | Schizophrenia | 0.144 | 0.034 | 4.277 | 1.900E-05 |
| Coronary artery disease | Sitting height | -0.073 | 0.017 | -4.249 | 2.150E-05 |
| Cigarette consumption | 25-Hydroxyvitamin D | -0.093 | 0.022 | -4.244 | 2.201E-05 |
| Smoking cessation | Birth weight | -0.122 | 0.029 | -4.232 | 2.319E-05 |
| HDL cholesterol | Childhood BMI | -0.208 | 0.049 | -4.217 | 2.480E-05 |
| Lifetime smoking | Anxious feeling | 0.105 | 0.025 | 4.195 | 2.728E-05 |
| Adult-onset asthma | Strenuous sports or other exercises | -0.132 | 0.032 | -4.195 | 2.730E-05 |
| Lifetime smoking | Childhood BMI | 0.143 | 0.034 | 4.191 | 2.783E-05 |
| Caffeine consumption | Childhood BMI | 0.192 | 0.046 | 4.185 | 2.846E-05 |
| Visceral adipose tissue | Sitting height | 0.066 | 0.016 | 4.184 | 2.860E-05 |
| Total cholesterol | Triglycerides | 0.487 | 0.117 | 4.184 | 2.870E-05 |
| Fasting insulin | Hip circumference | -0.120 | 0.029 | -4.180 | 2.920E-05 |
| Triglycerides | Hip circumference | 0.140 | 0.034 | 4.172 | 3.020E-05 |
| 10+ minutes vigorous activity | Sitting height | -0.079 | 0.019 | -4.160 | 3.150E-05 |
| 2-hour blood glucose | Waist-to-hip ratio | 0.149 | 0.036 | 4.150 | 3.300E-05 |
| Intake of carbohydrate | Moderate to vigorous physical activity levels | 0.150 | 0.036 | 4.137 | 3.516E-05 |
| Coffee consumption | Breakfast skipping | 0.193 | 0.047 | 4.134 | 3.570E-05 |
| Caffeine consumption | Major depressive disorder | -0.106 | 0.026 | -4.133 | 3.575E-05 |
| Smoking cessation | HbA1c | 0.140 | 0.034 | 4.133 | 3.588E-05 |
| Coffee consumption | Anxious feeling | -0.125 | 0.030 | -4.124 | 3.720E-05 |
| 2-hour blood glucose | 25-Hydroxyvitamin D | -0.165 | 0.040 | -4.120 | 3.760E-05 |
| Daytime sleepiness | Income | -0.108 | 0.026 | -4.120 | 3.830E-05 |
| Intake of fat | Moderate to vigorous physical activity levels | -0.173 | 0.042 | -4.111 | 3.940E-05 |
| Atrial fibrillation | Smoking initiation | 0.076 | 0.018 | 4.105 | 4.050E-05 |
| Morning person | Strenuous sports or other exercises | 0.095 | 0.023 | 4.097 | 4.190E-05 |
| Smoking initiation | Childhood BMI | 0.137 | 0.034 | 4.072 | 4.667E-05 |
| Anorexia nervosa | Intake of total sugar | 0.161 | 0.040 | 4.064 | 4.830E-05 |
| Caffeine consumption | Cigarette consumption | 0.130 | 0.032 | 4.053 | 5.057E-05 |
| Crohn's disease | Long sleep | 0.179 | 0.044 | 4.051 | 5.100E-05 |
| Anorexia nervosa | Leptin | -0.267 | 0.066 | -4.045 | 5.240E-05 |
| Lifetime smoking | Leisure sedentary behaviour (computer use) | -0.091 | 0.023 | -4.037 | 5.420E-05 |
| Bipolar disorder | Hand grip strength (right) | -0.086 | 0.021 | -4.036 | 5.450E-05 |
| 2-hour blood glucose | Hand grip strength (left) | -0.133 | 0.033 | -4.000 | 6.270E-05 |

| | | | | | |
|---|---|---|---|---|---|
| Schizophrenia | Alcohol consumption | 0.087 | 0.022 | 3.999 | 6.360E-05 |
| Age of initiation of regular smoking | Anxious feeling | -0.137 | 0.034 | -3.998 | 6.380E-05 |
| Cigarette consumption | Moderate to vigorous physical activity levels | -0.116 | 0.029 | -3.993 | 6.538E-05 |
| Smoking initiation | Anxious feeling | 0.098 | 0.025 | 3.988 | 6.661E-05 |
| Age of initiation of regular smoking | Daytime sleepiness | -0.140 | 0.035 | -3.981 | 6.860E-05 |
| Whole body fat mass | Life satisfaction | -0.073 | 0.018 | -3.977 | 6.990E-05 |
| Intelligence | Positive affect | 0.092 | 0.023 | 3.963 | 7.410E-05 |
| Moderate to vigorous physical activity levels | Standing height | -0.081 | 0.020 | -3.950 | 7.710E-05 |
| Coronary artery disease | Fasting glucose | 0.113 | 0.029 | 3.952 | 7.760E-05 |
| Caffeine consumption | Breakfast skipping | 0.186 | 0.047 | 3.948 | 7.875E-05 |
| Alcohol consumption | 2-hour blood glucose | -0.166 | 0.042 | -3.945 | 7.960E-05 |
| Alcohol consumption | Fasting insulin | -0.126 | 0.032 | -3.943 | 8.040E-05 |
| Leisure sedentary behaviour (computer use) | 10+ minutes vigorous activity | -0.122 | 0.031 | -3.940 | 8.070E-05 |
| Leisure sedentary behaviour (TV watching) | Hand grip strength (left) | -0.074 | 0.019 | -3.940 | 8.080E-05 |
| Adult-onset asthma | Short sleep | 0.132 | 0.033 | 3.941 | 8.110E-05 |
| Hand grip strength (left) | Anxious feeling | -0.082 | 0.021 | -3.932 | 8.430E-05 |
| Caffeine consumption | Life satisfaction | 0.111 | 0.028 | 3.932 | 8.442E-05 |
| Schizophrenia | Coffee consumption | -0.098 | 0.025 | -3.926 | 8.620E-05 |
| Daytime napping | Fasting insulin | 0.101 | 0.026 | 3.920 | 8.750E-05 |
| Leisure sedentary behaviour (TV watching) | Hand grip strength (right) | -0.075 | 0.019 | -3.900 | 9.490E-05 |
| Type 2 diabetes | Positive affect | -0.095 | 0.024 | -3.899 | 9.660E-05 |
| Triglycerides | Standing height | -0.080 | 0.021 | -3.889 | 1.010E-04 |
| 10+ minutes vigorous activity | Hand grip strength (right) | 0.092 | 0.024 | 3.880 | 1.040E-04 |
| Smoking cessation | Whole body fat-free mass | 0.093 | 0.024 | 3.864 | 1.114E-04 |
| Coffee consumption | 2-hour blood glucose | -0.210 | 0.055 | -3.861 | 1.130E-04 |
| Triglycerides | Life satisfaction | -0.094 | 0.024 | -3.862 | 1.130E-04 |
| Breakfast skipping | Sitting height | -0.105 | 0.027 | -3.853 | 1.170E-04 |
| Atrial fibrillation | Hand grip strength (left) | 0.088 | 0.023 | 3.848 | 1.190E-04 |
| Strenuous sports or other exercises | Leptin | -0.233 | 0.061 | -3.839 | 1.240E-04 |
| HDL cholesterol | Leptin | -0.246 | 0.064 | -3.837 | 1.250E-04 |
| Type 2 diabetes | Intake of total sugar | -0.135 | 0.035 | -3.836 | 1.250E-04 |
| Adult-onset asthma | Hand grip strength (left) | -0.100 | 0.026 | -3.834 | 1.260E-04 |
| Caffeine consumption | Fasting insulin | -0.150 | 0.039 | -3.825 | 1.306E-04 |
| Long sleep | Hip circumference | 0.094 | 0.025 | 3.823 | 1.320E-04 |
| Atrial fibrillation | Hand grip strength (right) | 0.090 | 0.023 | 3.821 | 1.330E-04 |
| Leisure sedentary behaviour (TV watching) | Total cholesterol | 0.111 | 0.029 | 3.810 | 1.380E-04 |
| Age of initiation of regular smoking | Hand grip strength (right) | 0.100 | 0.026 | 3.808 | 1.400E-04 |
| Total cholesterol | Sitting height | -0.094 | 0.025 | -3.804 | 1.420E-04 |

| | | | | | |
|---|---|---|---|---|---|
| 25-Hydroxyvitamin D | Income | -0.077 | 0.020 | -3.798 | 1.460E-04 |
| Adult-onset asthma | Income | -0.104 | 0.027 | -3.784 | 1.540E-04 |
| Coronary artery disease | Childhood BMI | 0.127 | 0.034 | 3.756 | 1.730E-04 |
| 2-hour blood glucose | Sitting height | -0.125 | 0.033 | -3.750 | 1.760E-04 |
| Leisure sedentary behaviour (TV watching) | LDL cholesterol | 0.133 | 0.036 | 3.750 | 1.790E-04 |
| Bipolar disorder | Strenuous sports or other exercises | 0.094 | 0.025 | 3.742 | 1.830E-04 |
| Daytime sleepiness | Whole body fat-free mass | 0.073 | 0.020 | 3.740 | 1.850E-04 |
| Leisure sedentary behaviour (computer use) | Hand grip strength (right) | 0.079 | 0.021 | 3.730 | 1.880E-04 |
| Schizophrenia | Leisure sedentary behaviour (computer use) | -0.075 | 0.020 | -3.734 | 1.880E-04 |
| Lifetime smoking | Birth weight | -0.070 | 0.019 | -3.721 | 1.981E-04 |
| Intake of fat | Educational attainment | -0.110 | 0.029 | -3.719 | 2.002E-04 |
| 10+ minutes vigorous activity | Positive affect | 0.116 | 0.031 | 3.710 | 2.040E-04 |
| Coronary artery disease | 10+ minutes vigorous activity | -0.105 | 0.028 | -3.714 | 2.040E-04 |
| Coronary artery disease | LDL cholesterol | 0.183 | 0.049 | 3.711 | 2.070E-04 |
| Fasting insulin | Income | -0.113 | 0.030 | -3.700 | 2.130E-04 |
| Short sleep | Fasting glucose | 0.119 | 0.032 | 3.701 | 2.150E-04 |
| LDL cholesterol | Standing height | -0.107 | 0.029 | -3.700 | 2.160E-04 |
| Adult-onset asthma | Atrial fibrillation | 0.121 | 0.033 | 3.690 | 2.250E-04 |
| Fasting glucose | Educational attainment | -0.075 | 0.021 | -3.676 | 2.370E-04 |
| Intelligence | Fasting insulin | -0.095 | 0.026 | -3.675 | 2.380E-04 |
| Daytime napping | 25-Hydroxyvitamin D | -0.071 | 0.019 | -3.670 | 2.420E-04 |
| Age of initiation of regular smoking | Fasting insulin | -0.117 | 0.032 | -3.669 | 2.440E-04 |
| Strenuous sports or other exercises | Anxious feeling | -0.124 | 0.034 | -3.667 | 2.450E-04 |
| Atrial fibrillation | Educational attainment | -0.067 | 0.018 | -3.666 | 2.460E-04 |
| Total cholesterol | Whole body fat-free mass | -0.098 | 0.027 | -3.666 | 2.460E-04 |
| Caffeine consumption | Positive affect | 0.104 | 0.028 | 3.661 | 2.511E-04 |
| Insomnia | Birth weight | -0.092 | 0.025 | -3.659 | 2.530E-04 |
| Schizophrenia | Smoking cessation | 0.108 | 0.030 | 3.655 | 2.570E-04 |
| Age of initiation of regular smoking | HbA1c | -0.133 | 0.036 | -3.645 | 2.670E-04 |
| Standing height | Life satisfaction | 0.059 | 0.016 | 3.642 | 2.710E-04 |
| Atrial fibrillation | Age of initiation of regular smoking | -0.094 | 0.026 | -3.639 | 2.740E-04 |
| Daytime sleepiness | Hand grip strength (left) | -0.089 | 0.024 | -3.630 | 2.820E-04 |
| Intelligence | Daytime sleepiness | -0.082 | 0.023 | -3.631 | 2.830E-04 |
| Leptin | Childhood BMI | 0.333 | 0.092 | 3.626 | 2.880E-04 |
| Strenuous sports or other exercises | Hand grip strength (right) | 0.084 | 0.023 | 3.626 | 2.880E-04 |
| Short sleep | Triglycerides | 0.116 | 0.032 | 3.625 | 2.890E-04 |
| Atrial fibrillation | Insomnia | 0.094 | 0.026 | 3.621 | 2.930E-04 |
| Fasting insulin | Whole body fat-free mass | -0.099 | 0.028 | -3.600 | 3.140E-04 |
| Triglycerides | Birth weight | -0.112 | 0.031 | -3.603 | 3.140E-04 |
| Cigarette consumption | Intake of fat | 0.139 | 0.039 | 3.602 | 3.160E-04 |
| Adult-onset asthma | Leisure sedentary behaviour | 0.107 | 0.030 | 3.589 | 3.320E-04 |

| | | | | | |
|---|---|---|---|---|---|
| | (computer use) | | | | |
| Intake of fat | Morning person | -0.131 | 0.037 | -3.587 | 3.347E-04 |
| Bipolar disorder | Intelligence | -0.069 | 0.019 | -3.585 | 3.370E-04 |
| Intelligence | Bipolar disorder | -0.069 | 0.019 | -3.585 | 3.370E-04 |
| Insomnia | Fasting glucose | 0.095 | 0.026 | 3.584 | 3.390E-04 |
| Long sleep | 2-hour blood glucose | 0.219 | 0.061 | 3.584 | 3.390E-04 |
| HbA1c | LDL cholesterol | 0.226 | 0.063 | 3.573 | 3.530E-04 |
| Visceral adipose tissue | Anxious feeling | -0.077 | 0.022 | -3.563 | 3.660E-04 |
| Morning person | Major depressive disorder | -0.078 | 0.022 | -3.561 | 3.690E-04 |
| BMI | Positive affect | -0.072 | 0.020 | -3.550 | 3.859E-04 |
| Smoking initiation | HbA1c | 0.080 | 0.023 | 3.545 | 3.930E-04 |
| 25-Hydroxyvitamin D | Childhood BMI | 0.110 | 0.031 | 3.541 | 3.980E-04 |
| Visceral adipose tissue | Birth weight | 0.066 | 0.019 | 3.533 | 4.110E-04 |
| Intake of total sugar | 25-Hydroxyvitamin D | 0.100 | 0.028 | 3.527 | 4.210E-04 |
| Atrial fibrillation | Short sleep | 0.092 | 0.026 | 3.524 | 4.250E-04 |
| 10+ minutes vigorous activity | Life satisfaction | 0.108 | 0.031 | 3.510 | 4.420E-04 |
| Atrial fibrillation | Birth weight | 0.085 | 0.024 | 3.508 | 4.510E-04 |
| HbA1c | Total cholesterol | 0.198 | 0.056 | 3.506 | 4.540E-04 |
| Ulcerative colitis | Major depressive disorder | 0.121 | 0.035 | 3.498 | 4.680E-04 |
| Lifetime smoking | Intake of fat | 0.134 | 0.038 | 3.493 | 4.772E-04 |
| HbA1c | Income | -0.098 | 0.028 | -3.493 | 4.780E-04 |
| Atrial fibrillation | Childhood BMI | 0.144 | 0.041 | 3.493 | 4.790E-04 |
| Intelligence | Hand grip strength (right) | 0.068 | 0.020 | 3.490 | 4.840E-04 |
| Coffee consumption | Fasting insulin | -0.129 | 0.037 | -3.487 | 4.880E-04 |
| Ulcerative colitis | Life satisfaction | -0.125 | 0.036 | -3.486 | 4.900E-04 |
| 25-Hydroxyvitamin D | Whole body fat mass | 0.057 | 0.016 | 3.484 | 4.940E-04 |
| BMI | Birth weight | 0.058 | 0.017 | 3.477 | 5.066E-04 |
| Total cholesterol | Childhood BMI | -0.182 | 0.052 | -3.477 | 5.070E-04 |
| BMI | 2-hour blood glucose | -0.120 | 0.034 | -3.471 | 5.188E-04 |
| Intelligence | HDL cholesterol | 0.093 | 0.027 | 3.470 | 5.210E-04 |
| Schizophrenia | Daytime sleepiness | 0.084 | 0.024 | 3.461 | 5.380E-04 |
| Coronary artery disease | Anxious feeling | 0.077 | 0.022 | 3.444 | 5.740E-04 |
| 10+ minutes vigorous activity | Fasting insulin | -0.138 | 0.040 | -3.440 | 5.820E-04 |
| Lifetime smoking | 10+ minutes vigorous activity | -0.089 | 0.026 | -3.437 | 5.893E-04 |
| Anorexia nervosa | HbA1c | -0.135 | 0.039 | -3.434 | 5.940E-04 |
| Triglycerides | Positive affect | -0.083 | 0.024 | -3.433 | 5.970E-04 |
| Smoking initiation | Intake of fat | 0.106 | 0.031 | 3.432 | 5.994E-04 |
| Intake of carbohydrate | Leisure sedentary behaviour (TV watching) | -0.096 | 0.028 | -3.430 | 6.040E-04 |
| Lifetime smoking | Daytime sleepiness | 0.094 | 0.027 | 3.427 | 6.107E-04 |
| Ulcerative colitis | Positive affect | -0.126 | 0.037 | -3.420 | 6.260E-04 |
| Daytime sleepiness | Strenuous sports or other exercises | -0.099 | 0.029 | -3.420 | 6.270E-04 |
| Type 2 diabetes | Moderate to vigorous physical activity levels | -0.082 | 0.024 | -3.408 | 6.530E-04 |

| | | | | | |
|---|---|---|---|---|---|
| Alcohol consumption | HbA1c | -0.091 | 0.027 | -3.397 | 6.821E-04 |
| Bipolar disorder | Insomnia | 0.080 | 0.024 | 3.397 | 6.830E-04 |
| Atrial fibrillation | HDL cholesterol | -0.113 | 0.033 | -3.383 | 7.180E-04 |
| Leptin | Hand grip strength (right) | -0.156 | 0.046 | -3.382 | 7.200E-04 |
| Crohn's disease | Anxious feeling | 0.115 | 0.034 | 3.378 | 7.300E-04 |
| BMI | Fasting insulin | -0.091 | 0.027 | -3.377 | 7.333E-04 |
| Whole body fat mass | Positive affect | -0.063 | 0.019 | -3.370 | 7.510E-04 |
| Leisure sedentary behaviour (TV watching) | HbA1c | 0.086 | 0.026 | 3.370 | 7.540E-04 |
| Morning person | Leisure sedentary behaviour (computer use) | -0.070 | 0.021 | -3.364 | 7.670E-04 |
| Adult-onset asthma | Hand grip strength (right) | -0.094 | 0.028 | -3.359 | 7.840E-04 |
| 2-hour blood glucose | Hand grip strength (right) | -0.115 | 0.034 | -3.350 | 8.090E-04 |
| 2-hour blood glucose | Childhood BMI | -0.220 | 0.066 | -3.340 | 8.380E-04 |
| Smoking cessation | Sitting height | -0.066 | 0.020 | -3.332 | 8.609E-04 |
| 10+ minutes vigorous activity | Major depressive disorder | -0.100 | 0.030 | -3.330 | 8.720E-04 |
| Daytime sleepiness | Hand grip strength (right) | -0.076 | 0.023 | -3.330 | 8.820E-04 |
| Crohn's disease | Major depressive disorder | 0.093 | 0.028 | 3.321 | 8.960E-04 |
| Intake of fat | Daytime sleepiness | 0.122 | 0.037 | 3.321 | 8.971E-04 |
| 25-Hydroxyvitamin D | Waist-to-hip ratio | -0.052 | 0.016 | -3.311 | 9.290E-04 |
| Fasting insulin | 25-Hydroxyvitamin D | -0.105 | 0.032 | -3.310 | 9.340E-04 |
| Type 2 diabetes | Intake of carbohydrate | -0.113 | 0.034 | -3.299 | 9.710E-04 |
| 2-hour blood glucose | Whole body fat mass | -0.113 | 0.034 | -3.300 | 9.790E-04 |
| BMI | Fasting glucose | -0.070 | 0.021 | -3.296 | 9.797E-04 |
| Age of initiation of regular smoking | 10+ minutes vigorous activity | 0.115 | 0.035 | 3.294 | 9.870E-04 |
| Fasting glucose | Waist-to-hip ratio | 0.090 | 0.028 | 3.283 | 1.030E-03 |
| Fasting insulin | Triglycerides | 0.284 | 0.087 | 3.280 | 1.030E-03 |
| Alcohol consumption | Smoking cessation | 0.108 | 0.033 | 3.282 | 1.032E-03 |
| Caffeine consumption | Standing height | 0.079 | 0.024 | 3.273 | 1.064E-03 |
| Daytime sleepiness | Triglycerides | 0.100 | 0.031 | 3.270 | 1.090E-03 |
| Short sleep | HDL cholesterol | -0.097 | 0.030 | -3.264 | 1.100E-03 |
| Bipolar disorder | Hip circumference | -0.060 | 0.018 | -3.248 | 1.160E-03 |
| Insomnia | HbA1c | 0.088 | 0.027 | 3.240 | 1.200E-03 |
| Age of initiation of regular smoking | Intake of fat | -0.142 | 0.044 | -3.236 | 1.210E-03 |
| Intelligence | Hip circumference | -0.051 | 0.016 | -3.233 | 1.220E-03 |
| Daytime sleepiness | Educational attainment | -0.067 | 0.021 | -3.230 | 1.230E-03 |
| LDL cholesterol | Educational attainment | -0.125 | 0.039 | -3.231 | 1.230E-03 |
| Insomnia | Sitting height | -0.058 | 0.018 | -3.225 | 1.260E-03 |
| Short sleep | Birth weight | -0.077 | 0.024 | -3.222 | 1.270E-03 |
| Type 2 diabetes | Standing height | -0.059 | 0.018 | -3.224 | 1.270E-03 |
| Adult-onset asthma | Childhood BMI | 0.144 | 0.045 | 3.221 | 1.280E-03 |
| Strenuous sports or other exercises | Fasting glucose | -0.104 | 0.032 | -3.221 | 1.280E-03 |
| Intake of carbohydrate | HDL cholesterol | -0.125 | 0.039 | -3.218 | 1.293E-03 |
| Intake of fat | HbA1c | 0.123 | 0.038 | 3.208 | 1.335E-03 |

| | | | | | |
|---|---|---|---|---|---|
| Breakfast skipping | Hand grip strength (right) | -0.098 | 0.031 | -3.192 | 1.410E-03 |
| Anorexia nervosa | Triglycerides | -0.122 | 0.038 | -3.189 | 1.430E-03 |
| Smoking cessation | Triglycerides | 0.141 | 0.044 | 3.187 | 1.437E-03 |
| Leisure sedentary behaviour (computer use) | Waist circumference | 0.058 | 0.018 | 3.160 | 1.580E-03 |
| Alcohol consumption | Intake of fat | -0.122 | 0.039 | -3.151 | 1.625E-03 |
| Sitting height | Major depressive disorder | -0.051 | 0.016 | -3.147 | 1.650E-03 |
| Crohn's disease | Schizophrenia | 0.099 | 0.031 | 3.141 | 1.680E-03 |
| Childhood BMI | Anxious feeling | -0.122 | 0.039 | -3.138 | 1.700E-03 |
| Smoking cessation | HDL cholesterol | -0.152 | 0.049 | -3.135 | 1.719E-03 |
| Caffeine consumption | Waist-to-hip ratio | 0.080 | 0.025 | 3.127 | 1.765E-03 |
| Alcohol consumption | Major depressive disorder | 0.073 | 0.024 | 3.126 | 1.770E-03 |
| Lifetime smoking | Hand grip strength (right) | -0.058 | 0.019 | -3.117 | 1.825E-03 |
| Leptin | Anxious feeling | -0.160 | 0.052 | -3.104 | 1.910E-03 |
| Smoking cessation | Leptin | 0.226 | 0.073 | 3.102 | 1.926E-03 |
| Intake of total sugar | Major depressive disorder | -0.100 | 0.032 | -3.088 | 2.019E-03 |
| Fasting insulin | Adiponectin | -0.831 | 0.270 | -3.080 | 2.100E-03 |
| Atrial fibrillation | Cigarette consumption | 0.088 | 0.029 | 3.062 | 2.200E-03 |
| Morning person | Short sleep | 0.074 | 0.024 | 3.059 | 2.220E-03 |
| Lifetime smoking | Sitting height | -0.046 | 0.015 | -3.053 | 2.267E-03 |
| Fasting glucose | Sitting height | 0.064 | 0.021 | 3.050 | 2.290E-03 |
| Standing height | Positive affect | 0.050 | 0.017 | 3.038 | 2.380E-03 |
| Age of initiation of regular smoking | Sitting height | 0.065 | 0.022 | 3.036 | 2.390E-03 |
| Caffeine consumption | Triglycerides | -0.114 | 0.038 | -3.033 | 2.419E-03 |
| Long sleep | 10+ minutes vigorous activity | -0.116 | 0.038 | -3.030 | 2.450E-03 |
| Fasting insulin | Anxious feeling | 0.100 | 0.033 | 3.020 | 2.530E-03 |
| Crohn's disease | Educational attainment | -0.090 | 0.030 | -3.015 | 2.570E-03 |
| Smoking initiation | Daytime sleepiness | 0.074 | 0.025 | 3.011 | 2.604E-03 |
| Crohn's disease | Daytime sleepiness | 0.110 | 0.037 | 3.008 | 2.630E-03 |
| Total cholesterol | Educational attainment | -0.099 | 0.033 | -3.005 | 2.650E-03 |
| Bipolar disorder | Whole body fat mass | -0.055 | 0.018 | -2.998 | 2.720E-03 |
| Strenuous sports or other exercises | Standing height | 0.054 | 0.018 | 2.996 | 2.740E-03 |
| Smoking cessation | Hand grip strength (right) | -0.084 | 0.028 | -2.989 | 2.800E-03 |
| Smoking cessation | Morning person | -0.085 | 0.029 | -2.985 | 2.837E-03 |
| Fasting glucose | Hip circumference | -0.075 | 0.025 | -2.984 | 2.840E-03 |
| Fasting glucose | Standing height | 0.061 | 0.020 | 2.977 | 2.910E-03 |
| 10+ minutes vigorous activity | HDL cholesterol | 0.108 | 0.037 | 2.960 | 3.050E-03 |
| Intake of fat | 25-Hydroxyvitamin D | -0.098 | 0.033 | -2.961 | 3.064E-03 |
| LDL cholesterol | Waist-to-hip ratio | 0.155 | 0.052 | 2.953 | 3.150E-03 |
| Type 2 diabetes | Hand grip strength (left) | -0.061 | 0.021 | -2.950 | 3.170E-03 |
| Type 2 diabetes | Alcohol consumption | -0.067 | 0.023 | -2.946 | 3.220E-03 |
| Type 2 diabetes | Schizophrenia | -0.055 | 0.019 | -2.944 | 3.240E-03 |
| Lifetime smoking | Total cholesterol | 0.096 | 0.033 | 2.939 | 3.292E-03 |
| Leisure sedentary behaviour (computer | Birth weight | 0.072 | 0.025 | 2.920 | 3.470E-03 |

| | | | | | |
|---|---|---|---|---|---|
| use) | | | | | |
| Smoking cessation | Intake of fat | 0.158 | 0.054 | 2.922 | 3.482E-03 |
| Coffee consumption | Positive affect | 0.083 | 0.029 | 2.914 | 3.570E-03 |
| Morning person | Daytime sleepiness | 0.063 | 0.022 | 2.909 | 3.630E-03 |
| Bipolar disorder | Age of initiation of regular smoking | -0.076 | 0.026 | -2.902 | 3.710E-03 |
| HbA1c | Leptin | 0.180 | 0.062 | 2.901 | 3.720E-03 |
| Crohn's disease | Life satisfaction | -0.087 | 0.030 | -2.897 | 3.770E-03 |
| Crohn's disease | Positive affect | -0.088 | 0.031 | -2.892 | 3.830E-03 |
| 25-Hydroxyvitamin D | Hand grip strength (left) | 0.053 | 0.018 | 2.890 | 3.860E-03 |
| Childhood-onset asthma | Smoking cessation | -0.099 | 0.034 | -2.886 | 3.900E-03 |
| Alcohol consumption | Cigarette consumption | 0.083 | 0.029 | 2.883 | 3.935E-03 |
| Type 2 diabetes | Hand grip strength (right) | -0.059 | 0.021 | -2.883 | 3.940E-03 |
| Type 2 diabetes | Intake of fat | 0.124 | 0.043 | 2.882 | 3.950E-03 |
| Age of initiation of regular smoking | Childhood BMI | -0.142 | 0.050 | -2.872 | 4.080E-03 |
| Crohn's disease | Bipolar disorder | 0.098 | 0.034 | 2.872 | 4.080E-03 |
| Intake of carbohydrate | Insomnia | -0.090 | 0.031 | -2.869 | 4.112E-03 |
| 10+ minutes vigorous activity | Standing height | -0.056 | 0.020 | -2.870 | 4.120E-03 |
| Leptin | Hand grip strength (left) | -0.136 | 0.048 | -2.863 | 4.200E-03 |
| Anorexia nervosa | Lifetime smoking | -0.083 | 0.029 | -2.853 | 4.330E-03 |
| Adult-onset asthma | Age of initiation of regular smoking | -0.108 | 0.038 | -2.848 | 4.390E-03 |
| Intake of fat | Income | -0.102 | 0.036 | -2.842 | 4.483E-03 |
| Short sleep | Leisure sedentary behaviour (computer use) | -0.075 | 0.026 | -2.840 | 4.510E-03 |
| Alcohol consumption | Income | 0.071 | 0.025 | 2.832 | 4.619E-03 |
| Anorexia nervosa | Intelligence | 0.085 | 0.030 | 2.832 | 4.620E-03 |
| Intelligence | Anorexia Nervosa | 0.085 | 0.030 | 2.832 | 4.620E-03 |
| Coffee consumption | Major depressive disorder | -0.076 | 0.027 | -2.820 | 4.800E-03 |
| Intake of carbohydrate | 10+ minutes vigorous activity | 0.106 | 0.038 | 2.820 | 4.800E-03 |
| BMI | Moderate to vigorous physical activity levels | -0.060 | 0.021 | -2.820 | 4.807E-03 |
| Crohn's disease | Visceral adipose tissue | 0.079 | 0.028 | 2.819 | 4.820E-03 |
| LDL cholesterol | Sitting height | -0.082 | 0.029 | -2.816 | 4.870E-03 |
| Cigarette consumption | Daytime sleepiness | 0.081 | 0.029 | 2.815 | 4.874E-03 |
| Anorexia nervosa | Long sleep | -0.114 | 0.041 | -2.813 | 4.910E-03 |
| Caffeine consumption | HDL cholesterol | 0.101 | 0.036 | 2.811 | 4.932E-03 |
| Adult-onset asthma | Whole body fat-free mass | 0.073 | 0.026 | 2.811 | 4.940E-03 |
| Insomnia | Fasting insulin | 0.087 | 0.031 | 2.806 | 5.010E-03 |
| Coffee consumption | Life satisfaction | 0.080 | 0.029 | 2.805 | 5.030E-03 |
| Intake of fat | Waist-to-hip ratio | 0.086 | 0.031 | 2.803 | 5.060E-03 |
| Coffee consumption | Leptin | 0.163 | 0.058 | 2.792 | 5.240E-03 |
| Sitting height | Anxious feeling | -0.052 | 0.019 | -2.789 | 5.290E-03 |
| Insomnia | Triglycerides | 0.075 | 0.027 | 2.781 | 5.430E-03 |
| Intake of total sugar | Positive affect | 0.093 | 0.034 | 2.762 | 5.741E-03 |
| Daytime sleepiness | 25-Hydroxyvitamin D | -0.062 | 0.022 | -2.760 | 5.860E-03 |

| | | | | | |
|---|---|---|---|---|---|
| Intake of fat | Daytime napping | 0.085 | 0.031 | 2.751 | 5.944E-03 |
| 10+ minutes vigorous activity | Educational attainment | 0.067 | 0.024 | 2.740 | 6.090E-03 |
| Daytime sleepiness | HDL cholesterol | -0.088 | 0.032 | -2.740 | 6.190E-03 |
| Smoking cessation | Hand grip strength (left) | -0.080 | 0.029 | -2.731 | 6.317E-03 |
| Intake of fat | Body fat percentage | 0.085 | 0.031 | 2.729 | 6.346E-03 |
| Coffee consumption | Standing height | 0.066 | 0.024 | 2.728 | 6.370E-03 |
| Strenuous sports or other exercises | HbA1c | -0.085 | 0.031 | -2.727 | 6.390E-03 |
| Ulcerative colitis | Childhood BMI | -0.172 | 0.063 | -2.724 | 6.460E-03 |
| Breakfast skipping | Daytime napping | 0.090 | 0.033 | 2.718 | 6.570E-03 |
| Long sleep | Leptin | 0.216 | 0.080 | 2.708 | 6.770E-03 |
| Intake of fat | Whole body fat mass | 0.089 | 0.033 | 2.707 | 6.782E-03 |
| Morning person | Fasting insulin | -0.082 | 0.030 | -2.706 | 6.820E-03 |
| Age of initiation of regular smoking | Leptin | -0.172 | 0.064 | -2.701 | 6.910E-03 |
| Cigarette consumption | Hand grip strength (left) | -0.058 | 0.022 | -2.700 | 6.941E-03 |
| Alcohol consumption | Anxious feeling | 0.072 | 0.027 | 2.699 | 6.959E-03 |
| Ulcerative colitis | Bipolar disorder | 0.093 | 0.035 | 2.685 | 7.250E-03 |
| 25-Hydroxyvitamin D | Hand grip strength (right) | 0.048 | 0.018 | 2.684 | 7.270E-03 |
| Smoking cessation | LDL cholesterol | 0.169 | 0.063 | 2.684 | 7.271E-03 |
| Intake of carbohydrate | Whole body fat-free mass | -0.068 | 0.025 | -2.682 | 7.323E-03 |
| Triglycerides | Leptin | 0.210 | 0.078 | 2.681 | 7.350E-03 |
| Hip circumference | Life satisfaction | -0.050 | 0.019 | -2.680 | 7.360E-03 |
| Daytime napping | 10+ minutes vigorous activity | -0.073 | 0.028 | -2.660 | 7.730E-03 |
| Intake of fat | Waist circumference | 0.086 | 0.032 | 2.662 | 7.770E-03 |
| Atrial fibrillation | Leisure sedentary behaviour (TV watching) | 0.055 | 0.021 | 2.662 | 7.770E-03 |
| Adiponectin | Waist-to-hip ratio | -0.286 | 0.108 | -2.662 | 7.800E-03 |
| Adult-onset asthma | Standing height | -0.062 | 0.023 | -2.661 | 7.800E-03 |
| Caffeine consumption | Insomnia | -0.092 | 0.035 | -2.654 | 7.952E-03 |
| Total cholesterol | Waist-to-hip ratio | 0.102 | 0.039 | 2.654 | 7.960E-03 |
| BMI | Hand grip strength (right) | -0.045 | 0.017 | -2.651 | 8.017E-03 |
| 2-hour blood glucose | Adiponectin | -0.651 | 0.246 | -2.650 | 8.080E-03 |
| Crohn's disease | Body fat percentage | 0.075 | 0.028 | 2.647 | 8.110E-03 |
| Childhood-onset asthma | Bipolar disorder | 0.075 | 0.028 | 2.641 | 8.280E-03 |
| Adiponectin | Hip circumference | 0.250 | 0.095 | 2.636 | 8.400E-03 |
| Daytime napping | Fasting glucose | 0.067 | 0.025 | 2.630 | 8.500E-03 |
| Long sleep | HbA1c | 0.094 | 0.036 | 2.626 | 8.650E-03 |
| Atrial fibrillation | Income | -0.061 | 0.023 | -2.623 | 8.720E-03 |
| HDL cholesterol | Adiponectin | 0.691 | 0.265 | 2.612 | 8.990E-03 |
| Breakfast skipping | 10+ minutes vigorous activity | -0.107 | 0.041 | -2.605 | 9.190E-03 |
| Bipolar disorder | Hand grip strength (left) | -0.059 | 0.023 | -2.605 | 9.200E-03 |
| Intake of total sugar | Strenuous sports or other exercises | 0.096 | 0.037 | 2.603 | 9.255E-03 |
| Anorexia nervosa | Breakfast skipping | -0.115 | 0.044 | -2.600 | 9.320E-03 |
| Coronary artery disease | Intake of total sugar | -0.079 | 0.030 | -2.600 | 9.330E-03 |
| Lifetime smoking | LDL cholesterol | 0.105 | 0.040 | 2.595 | 9.454E-03 |

| | | | | | |
|---|---|---|---|---|---|
| Birth weight | Anxious feeling | -0.065 | 0.025 | -2.589 | 9.620E-03 |
| Intake of total sugar | Life satisfaction | 0.085 | 0.033 | 2.578 | 9.928E-03 |
| Intake of fat | Childhood BMI | -0.159 | 0.062 | -2.573 | 0.010 |
| Bipolar disorder | Leisure sedentary behaviour (computer use) | 0.062 | 0.024 | 2.572 | 0.010 |
| Childhood-onset asthma | Moderate to vigorous physical activity levels | -0.074 | 0.029 | -2.571 | 0.010 |
| Bipolar disorder | Fasting insulin | -0.077 | 0.030 | -2.570 | 0.010 |
| HbA1c | Anxious feeling | -0.073 | 0.028 | -2.569 | 0.010 |
| Breakfast skipping | Hand grip strength (left) | -0.080 | 0.031 | -2.562 | 0.010 |
| Bipolar disorder | Morning person | -0.056 | 0.022 | -2.560 | 0.011 |
| Adult-onset asthma | Bipolar disorder | 0.073 | 0.029 | 2.555 | 0.011 |
| Strenuous sports or other exercises | 2-hour blood glucose | -0.132 | 0.052 | -2.556 | 0.011 |
| Short sleep | 10+ minutes vigorous activity | -0.081 | 0.032 | -2.553 | 0.011 |
| Type 2 diabetes | Adiponectin | -0.265 | 0.104 | -2.553 | 0.011 |
| Crohn's disease | Whole body fat mass | 0.068 | 0.027 | 2.548 | 0.011 |
| Triglycerides | Whole body fat-free mass | 0.079 | 0.031 | 2.545 | 0.011 |
| Breakfast skipping | 25-Hydroxyvitamin D | -0.076 | 0.030 | -2.541 | 0.011 |
| Long sleep | Triglycerides | 0.130 | 0.051 | 2.543 | 0.011 |
| Alcohol consumption | 25-Hydroxyvitamin D | 0.058 | 0.023 | 2.541 | 0.011 |
| Sitting height | Life satisfaction | 0.046 | 0.018 | 2.536 | 0.011 |
| Bipolar disorder | Whole body fat-free mass | -0.045 | 0.018 | -2.535 | 0.011 |
| Whole body fat-free mass | Educational attainment | -0.035 | 0.014 | -2.530 | 0.011 |
| Intake of fat | Positive affect | -0.092 | 0.036 | -2.526 | 0.012 |
| Bipolar disorder | 25-Hydroxyvitamin D | -0.062 | 0.025 | -2.524 | 0.012 |
| 10+ minutes vigorous activity | Whole body fat-free mass | -0.054 | 0.022 | -2.520 | 0.012 |
| Insomnia | Total cholesterol | 0.096 | 0.038 | 2.521 | 0.012 |
| Insomnia | Childhood BMI | -0.103 | 0.041 | -2.512 | 0.012 |
| Total cholesterol | Income | -0.104 | 0.042 | -2.509 | 0.012 |
| LDL cholesterol | Income | -0.120 | 0.048 | -2.507 | 0.012 |
| Crohn's disease | Smoking initiation | 0.068 | 0.027 | 2.498 | 0.013 |
| Insomnia | LDL cholesterol | 0.093 | 0.037 | 2.493 | 0.013 |
| Smoking initiation | Moderate to vigorous physical activity levels | 0.061 | 0.025 | 2.491 | 0.013 |
| Bipolar disorder | Body fat percentage | -0.046 | 0.019 | -2.485 | 0.013 |
| Long sleep | HDL cholesterol | -0.114 | 0.046 | -2.483 | 0.013 |
| 10+ minutes vigorous activity | Leptin | -0.178 | 0.072 | -2.480 | 0.013 |
| Fasting glucose | 25-Hydroxyvitamin D | -0.070 | 0.028 | -2.480 | 0.013 |
| Alcohol consumption | Triglycerides | -0.097 | 0.039 | -2.481 | 0.013 |
| BMI | Hand grip strength (left) | -0.042 | 0.017 | -2.476 | 0.013 |
| Crohn's disease | Daytime napping | 0.080 | 0.032 | 2.472 | 0.014 |
| Morning person | Sitting height | -0.037 | 0.015 | -2.469 | 0.014 |
| Schizophrenia | Hand grip strength (left) | -0.049 | 0.020 | -2.465 | 0.014 |
| Type 2 diabetes | Morning person | 0.050 | 0.020 | 2.464 | 0.014 |

| | | | | | |
|---|---|---|---|---|---|
| Coffee consumption | Leisure sedentary behaviour (TV watching) | -0.058 | 0.024 | -2.459 | 0.014 |
| Leisure sedentary behaviour (computer use) | Whole body fat mass | 0.046 | 0.019 | 2.460 | 0.014 |
| Age of initiation of regular smoking | Hand grip strength (left) | 0.066 | 0.027 | 2.443 | 0.015 |
| Cigarette consumption | Fasting insulin | 0.079 | 0.032 | 2.442 | 0.015 |
| Caffeine consumption | Sitting height | 0.059 | 0.024 | 2.439 | 0.015 |
| Childhood-onset asthma | Income | 0.062 | 0.025 | 2.436 | 0.015 |
| Crohn's disease | Intelligence | -0.077 | 0.032 | -2.432 | 0.015 |
| Childhood BMI | Educational attainment | -0.072 | 0.030 | -2.422 | 0.015 |
| Intelligence | Triglycerides | -0.061 | 0.025 | -2.424 | 0.015 |
| Leisure sedentary behaviour (TV watching) | Fasting glucose | 0.069 | 0.028 | 2.420 | 0.015 |
| Schizophrenia | Caffeine consumption | -0.060 | 0.025 | -2.423 | 0.015 |
| Smoking cessation | Total cholesterol | 0.130 | 0.054 | 2.421 | 0.015 |
| Intake of fat | Visceral adipose tissue | 0.084 | 0.035 | 2.421 | 0.015 |
| Triglycerides | Adiponectin | -0.523 | 0.216 | -2.418 | 0.016 |
| Daytime sleepiness | Leptin | 0.139 | 0.058 | 2.410 | 0.016 |
| 10+ minutes vigorous activity | Childhood BMI | 0.107 | 0.044 | 2.410 | 0.016 |
| Total cholesterol | Birth weight | -0.078 | 0.033 | -2.401 | 0.016 |
| Moderate to vigorous physical activity levels | Waist-to-hip ratio | -0.049 | 0.021 | -2.390 | 0.017 |
| Coffee consumption | Strenuous sports or other exercises | 0.080 | 0.033 | 2.387 | 0.017 |
| Adult-onset asthma | Coronary artery disease | 0.071 | 0.030 | 2.381 | 0.017 |
| Atrial fibrillation | Coffee consumption | 0.067 | 0.028 | 2.371 | 0.018 |
| LDL cholesterol | Birth weight | -0.095 | 0.040 | -2.367 | 0.018 |
| Schizophrenia | Waist-to-hip ratio | -0.039 | 0.017 | -2.367 | 0.018 |
| BMI | Bipolar disorder | -0.043 | 0.018 | -2.364 | 0.018 |
| Intake of fat | Life satisfaction | -0.084 | 0.036 | -2.360 | 0.018 |
| Anorexia nervosa | Short sleep | 0.077 | 0.033 | 2.357 | 0.018 |
| Moderate to vigorous physical activity levels | Leptin | -0.164 | 0.070 | -2.350 | 0.019 |
| Alcohol consumption | Life satisfaction | -0.061 | 0.026 | -2.343 | 0.019 |
| Coronary artery disease | Adiponectin | -0.216 | 0.092 | -2.340 | 0.019 |
| Intake of total sugar | Leisure sedentary behaviour (TV watching) | -0.063 | 0.027 | -2.338 | 0.019 |
| Daytime napping | Hand grip strength (left) | -0.050 | 0.021 | -2.330 | 0.020 |
| Fasting insulin | Standing height | 0.061 | 0.026 | 2.330 | 0.020 |
| Schizophrenia | 10+ minutes vigorous activity | 0.067 | 0.029 | 2.332 | 0.020 |
| Intelligence | Alcohol consumption | 0.052 | 0.022 | 2.329 | 0.020 |
| Breakfast skipping | Leisure sedentary behaviour (computer use) | -0.080 | 0.035 | -2.323 | 0.020 |
| Short sleep | Moderate to vigorous physical activity levels | 0.072 | 0.031 | 2.322 | 0.020 |

| | | | | | |
|---|---|---|---|---|---|
| 25-Hydroxyvitamin D | Visceral adipose tissue | 0.039 | 0.017 | 2.320 | 0.020 |
| Adiponectin | Whole body fat mass | 0.161 | 0.070 | 2.318 | 0.020 |
| Adiponectin | Birth weight | 0.265 | 0.115 | 2.304 | 0.021 |
| Fasting glucose | Adiponectin | -0.326 | 0.142 | -2.301 | 0.021 |
| Adult-onset asthma | Cigarette consumption | 0.100 | 0.044 | 2.297 | 0.022 |
| Total cholesterol | Major depressive disorder | 0.081 | 0.035 | 2.294 | 0.022 |
| Cigarette consumption | LDL cholesterol | 0.100 | 0.044 | 2.288 | 0.022 |
| Cigarette consumption | Total cholesterol | 0.086 | 0.038 | 2.285 | 0.022 |
| Childhood-onset asthma | Alcohol consumption | -0.059 | 0.026 | -2.285 | 0.022 |
| Atrial fibrillation | Life satisfaction | -0.057 | 0.025 | -2.282 | 0.023 |
| Fasting glucose | Anxious feeling | 0.082 | 0.036 | 2.278 | 0.023 |
| Intake of carbohydrate | 2-hour blood glucose | 0.127 | 0.056 | 2.275 | 0.023 |
| Age of initiation of regular smoking | Intake of total sugar | 0.092 | 0.040 | 2.271 | 0.023 |
| Moderate to vigorous physical activity levels | Fasting insulin | -0.086 | 0.038 | -2.270 | 0.023 |
| Sitting height | Positive affect | 0.041 | 0.018 | 2.262 | 0.024 |
| HDL cholesterol | Total cholesterol | 0.227 | 0.100 | 2.255 | 0.024 |
| Leisure sedentary behaviour (computer use) | Hand grip strength (left) | 0.048 | 0.021 | 2.260 | 0.024 |
| LDL cholesterol | Childhood BMI | -0.127 | 0.057 | -2.252 | 0.024 |
| Smoking cessation | Moderate to vigorous physical activity levels | 0.086 | 0.038 | 2.252 | 0.024 |
| Age of initiation of regular smoking | Birth weight | 0.063 | 0.028 | 2.248 | 0.025 |
| BMI | Sitting height | -0.031 | 0.014 | -2.247 | 0.025 |
| Hip circumference | Positive affect | -0.043 | 0.019 | -2.246 | 0.025 |
| Caffeine consumption | Moderate to vigorous physical activity levels | -0.075 | 0.033 | -2.239 | 0.025 |
| Cigarette consumption | Hand grip strength (right) | -0.047 | 0.021 | -2.237 | 0.025 |
| Fasting insulin | Life satisfaction | -0.068 | 0.030 | -2.230 | 0.026 |
| Ulcerative colitis | Whole body fat mass | -0.063 | 0.028 | -2.233 | 0.026 |
| Fasting insulin | Leptin | 0.177 | 0.079 | 2.230 | 0.026 |
| Adult-onset asthma | Triglycerides | 0.079 | 0.035 | 2.224 | 0.026 |
| Adiponectin | Body fat percentage | 0.153 | 0.069 | 2.223 | 0.026 |
| Daytime napping | Sitting height | 0.034 | 0.015 | 2.220 | 0.026 |
| Breakfast skipping | 2-hour blood glucose | -0.138 | 0.062 | -2.220 | 0.026 |
| HbA1c | Triglycerides | 0.103 | 0.046 | 2.220 | 0.026 |
| Leisure sedentary behaviour (computer use) | Fasting glucose | -0.060 | 0.027 | -2.220 | 0.026 |
| Alcohol consumption | Positive affect | -0.060 | 0.027 | -2.217 | 0.027 |
| Long sleep | Standing height | -0.052 | 0.024 | -2.200 | 0.028 |
| Coffee consumption | Adiponectin | 0.265 | 0.121 | 2.187 | 0.029 |
| Leisure sedentary behaviour (TV watching) | Anxious feeling | 0.053 | 0.024 | 2.190 | 0.029 |
| Intake of carbohydrate | Anxious feeling | 0.069 | 0.032 | 2.182 | 0.029 |

| | | | | | |
|---|---|---|---|---|---|
| Intelligence | HbA1c | -0.056 | 0.026 | -2.180 | 0.029 |
| Schizophrenia | Age of initiation of regular smoking | -0.056 | 0.026 | -2.180 | 0.029 |
| Bipolar disorder | 10+ minutes vigorous activity | 0.066 | 0.030 | 2.179 | 0.029 |
| Anorexia nervosa | Insomnia | 0.072 | 0.033 | 2.175 | 0.030 |
| Fasting glucose | Income | -0.058 | 0.027 | -2.174 | 0.030 |
| Moderate to vigorous physical activity levels | Childhood BMI | 0.088 | 0.040 | 2.170 | 0.030 |
| Caffeine consumption | Adiponectin | 0.298 | 0.138 | 2.160 | 0.031 |
| Intake of fat | Fasting insulin | 0.133 | 0.062 | 2.159 | 0.031 |
| Adult-onset asthma | Leptin | 0.141 | 0.065 | 2.158 | 0.031 |
| Daytime sleepiness | HbA1c | 0.062 | 0.029 | 2.150 | 0.031 |
| Bipolar disorder | Moderate to vigorous physical activity levels | 0.064 | 0.030 | 2.149 | 0.032 |
| Type 2 diabetes | 25-Hydroxyvitamin D | -0.045 | 0.021 | -2.149 | 0.032 |
| BMI | Adiponectin | 0.143 | 0.067 | 2.149 | 0.032 |
| Fasting insulin | Waist circumference | 0.055 | 0.026 | 2.140 | 0.032 |
| Adiponectin | 25-Hydroxyvitamin D | 0.287 | 0.134 | 2.140 | 0.032 |
| Standing height | Childhood BMI | -0.075 | 0.035 | -2.138 | 0.033 |
| Childhood-onset asthma | Intake of fat | -0.081 | 0.038 | -2.130 | 0.033 |
| Coffee consumption | HbA1c | 0.065 | 0.030 | 2.121 | 0.034 |
| Smoking cessation | Anxious feeling | 0.082 | 0.039 | 2.120 | 0.034 |
| HDL cholesterol | 25-Hydroxyvitamin D | 0.068 | 0.032 | 2.111 | 0.035 |
| LDL cholesterol | Body fat percentage | 0.100 | 0.047 | 2.111 | 0.035 |
| 10+ minutes vigorous activity | Income | 0.068 | 0.032 | 2.110 | 0.035 |
| Adult-onset asthma | Intake of fat | -0.087 | 0.041 | -2.107 | 0.035 |
| Adult-onset asthma | Smoking cessation | -0.088 | 0.042 | -2.101 | 0.036 |
| Intelligence | Leptin | -0.104 | 0.050 | -2.100 | 0.036 |
| BMI | Leisure sedentary behaviour (computer use) | 0.053 | 0.018 | 2.099 | 0.036 |
| Visceral adipose tissue | Standing height | 0.032 | 0.015 | 2.092 | 0.036 |
| Triglycerides | Sitting height | -0.047 | 0.023 | -2.089 | 0.037 |
| Visceral adipose tissue | Hand grip strength (right) | -0.042 | 0.020 | -2.088 | 0.037 |
| Type 2 diabetes | LDL cholesterol | 0.112 | 0.054 | 2.086 | 0.037 |
| Intake of fat | Hip circumference | 0.067 | 0.032 | 2.085 | 0.037 |
| Daytime napping | HbA1c | 0.049 | 0.023 | 2.080 | 0.038 |
| Anorexia nervosa | LDL cholesterol | -0.110 | 0.053 | -2.075 | 0.038 |
| Hand grip strength (left) | Childhood BMI | 0.080 | 0.038 | 2.075 | 0.038 |
| Breakfast skipping | Triglycerides | 0.078 | 0.038 | 2.072 | 0.038 |
| Adult-onset asthma | Moderate to vigorous physical activity levels | -0.076 | 0.037 | -2.070 | 0.038 |
| Coronary artery disease | Total cholesterol | 0.116 | 0.056 | 2.068 | 0.039 |
| Bipolar disorder | Sitting height | -0.039 | 0.019 | -2.065 | 0.039 |
| Daytime napping | Total cholesterol | 0.065 | 0.032 | 2.060 | 0.039 |
| Bipolar disorder | Waist circumference | -0.037 | 0.018 | -2.063 | 0.039 |

| | | | | | |
|---|---|---|---|---|---|
| Atrial fibrillation | Fasting insulin | -0.064 | 0.031 | -2.062 | 0.039 |
| Crohn's disease | Cigarette consumption | 0.074 | 0.036 | 2.060 | 0.039 |
| Daytime napping | Adiponectin | -0.189 | 0.092 | -2.060 | 0.039 |
| Smoking cessation | Intake of total sugar | -0.088 | 0.043 | -2.060 | 0.039 |
| Childhood-onset asthma | Atrial fibrillation | 0.060 | 0.029 | 2.059 | 0.040 |
| HDL cholesterol | Birth weight | 0.061 | 0.030 | 2.058 | 0.040 |
| Coronary artery disease | Moderate to vigorous physical activity levels | -0.057 | 0.028 | -2.055 | 0.040 |
| Schizophrenia | Breakfast skipping | 0.062 | 0.030 | 2.056 | 0.040 |
| Smoking initiation | Leptin | 0.099 | 0.048 | 2.055 | 0.040 |
| Alcohol consumption | Hip circumference | -0.047 | 0.023 | -2.053 | 0.040 |
| Crohn's disease | Sitting height | 0.048 | 0.024 | 2.052 | 0.040 |
| Alcohol consumption | Educational attainment | 0.041 | 0.020 | 2.050 | 0.040 |
| Adult-onset asthma | 10+ minutes vigorous activity | -0.083 | 0.041 | -2.049 | 0.041 |
| Intake of total sugar | Total cholesterol | -0.096 | 0.047 | -2.047 | 0.041 |
| Alcohol consumption | 10+ minutes vigorous activity | 0.058 | 0.028 | 2.046 | 0.041 |
| Adult-onset asthma | HDL cholesterol | -0.075 | 0.037 | -2.045 | 0.041 |
| Visceral adipose tissue | Hand grip strength (left) | -0.042 | 0.021 | -2.038 | 0.042 |
| 10+ minutes vigorous activity | Triglycerides | -0.062 | 0.030 | -2.040 | 0.042 |
| 2-hour blood glucose | Triglycerides | 0.139 | 0.069 | 2.030 | 0.042 |
| Breakfast skipping | Morning person | -0.064 | 0.032 | -2.017 | 0.044 |
| Waist-to-hip ratio | Birth weight | -0.039 | 0.019 | -2.015 | 0.044 |
| Adult-onset asthma | Long sleep | 0.083 | 0.041 | 2.012 | 0.044 |
| Atrial fibrillation | Positive affect | -0.051 | 0.025 | -2.006 | 0.045 |
| Strenuous sports or other exercises | LDL cholesterol | -0.099 | 0.049 | -2.006 | 0.045 |
| Strenuous sports or other exercises | Childhood BMI | 0.082 | 0.041 | 2.004 | 0.045 |
| Adult-onset asthma | Schizophrenia | 0.057 | 0.029 | 2.001 | 0.045 |
| Caffeine consumption | Fasting glucose | -0.076 | 0.038 | -1.999 | 0.046 |
| Smoking initiation | Standing height | -0.024 | 0.012 | -1.997 | 0.046 |
| Standing height | Anxious feeling | -0.036 | 0.018 | -1.994 | 0.046 |
| Coffee consumption | Sitting height | 0.047 | 0.024 | 1.990 | 0.047 |
| Age of initiation of regular smoking | Fasting glucose | -0.062 | 0.031 | -1.986 | 0.047 |
| Childhood-onset asthma | Age of initiation of regular smoking | 0.063 | 0.032 | 1.984 | 0.047 |
| Crohn's disease | Waist circumference | 0.053 | 0.027 | 1.977 | 0.048 |
| Caffeine consumption | Short sleep | -0.057 | 0.029 | -1.974 | 0.048 |
| Coffee consumption | Moderate to vigorous physical activity levels | -0.068 | 0.034 | -1.973 | 0.049 |
| Crohn's disease | Total cholesterol | -0.089 | 0.045 | -1.971 | 0.049 |
| Smoking cessation | 10+ minutes vigorous activity | -0.082 | 0.042 | -1.968 | 0.049 |
| Intake of carbohydrate | Short sleep | -0.064 | 0.033 | -1.961 | 0.050 |

## Table S4. The summary of the MR results in the discovery phase

| Exposure | Used SNPs | I^2(%)[a] | IVW method | | | | | Weighted median method | | | | | MR-Egger method | | | | | MR PRESSO model | | | | | *P* value for heterogeneity[b] | *P* value for pleiotropy[c] |
|---|---|---|---|---|---|---|---|---|---|---|---|---|---|---|---|---|---|---|---|---|---|---|---|---|
| | | | 95%CI | OR | *P* value | SE | BETA | 95%CI | OR | *P* value | SE | BETA | 95%CI | OR | *P* value | SE | BETA | 95%CI | OR | *P* value | SE | BETA | | |
| **Daily habits** | | | | | | | | | | | | | | | | | | | | | | | | |
| Alcohol consumption | 91 | 26.329 | 0.788-1.155 | 0.954 | 0.632 | 0.097 | -0.047 | 0.625-1.100 | 0.829 | 0.194 | 0.144 | -0.187 | 0.628-1.325 | 0.912 | 0.630 | 0.191 | -0.092 | 0.791-1.151 | 0.955 | 0.627 | 0.096 | -0.047 | 0.011 | 0.782 |
| Coffee consumption | 29 | 10.811 | 0.714-1.108 | 0.889 | 0.295 | 0.112 | -0.118 | 0.658-1.280 | 0.918 | 0.614 | 0.170 | -0.086 | 0.824-2.107 | 1.318 | 0.259 | 0.239 | 0.276 | 0.694-1.454 | 0.889 | 0.361 | 0.127 | -0.118 | 0.255 | 0.067 |
| Caffeine consumption | 26 | 9.464 | 0.773-1.057 | 0.904 | 0.205 | 0.08 | -0.101 | 0.727-1.169 | 0.922 | 0.504 | 0.121 | -0.081 | 0.904-1.773 | 1.266 | 0.183 | 0.172 | 0.236 | 0.754-1.083 | 0.904 | 0.284 | 0.092 | -0.101 | 0.277 | 0.068 |
| Smoking initiation | 347 | 28.523 | 1.217-1.383 | 1.297 | 1.376E-15 | 0.033 | 0.260 | 1.125-1.332 | 1.224 | 2.898E-06 | 0.043 | 0.202 | 0.973-1.648 | 1.266 | 0.080 | 0.134 | 0.236 | 1.216-1.378 | 1.294 | 1.009E-14 | 0.032 | 0.258 | 1.075E-06 | 0.851 |
| Lifetime smoking | 119 | 31.540 | 1.846-2.794 | 2.271 | 8.561E-15 | 0.106 | 0.820 | 1.478-2.601 | 1.961 | 3.007E-06 | 0.144 | 0.673 | 0.663-3.339 | 1.488 | 0.337 | 0.412 | 0.398 | 1.825-2.727 | 2.231 | 1.890E-12 | 0.102 | 0.802 | 6.644E-04 | 0.291 |
| Cigarette consumption | 53 | 7.712 | 1.077-1.323 | 1.194 | 7.701E-04 | 0.053 | 0.177 | 0.923-1.295 | 1.093 | 0.301 | 0.086 | 0.089 | 0.850-1.256 | 1.033 | 0.746 | 0.100 | 0.032 | 1.071-1.332 | 1.194 | 2.342E-03 | 0.056 | 0.178 | 0.282 | 0.087 |
| Age of initiation of smoking | 7 | 270.622 | 0.563-1.282 | 0.849 | 0.436 | 0.210 | -0.163 | 0.475-1.295 | 0.784 | 0.342 | 0.256 | -0.243 | 0.507-43.779 | 4.710 | 0.231 | 1.138 | 1.550 | 0.626-1.328 | 0.911 | 0.642 | 0.192 | -0.093 | 0.899 | 0.186 |
| Smoking cessation | 24 | 18.400 | 1.082-1.377 | 1.221 | 1.170E-03 | 0.061 | 0.200 | 1.044-1.473 | 1.240 | 0.015 | 0.088 | 0.215 | 0.819-1.655 | 1.164 | 0.407 | 0.180 | 0.152 | 1.068-1.395 | 1.221 | 7.583E-03 | 0.068 | 0.200 | 0.170 | 0.776 |
| Intake of total sugar | 9 | 47.507 | 0.716-1.248 | 0.946 | 0.693 | 0.142 | -0.056 | 0.669-1.550 | 1.019 | 0.931 | 0.214 | 0.019 | 0.026-3.405 | 0.298 | 0.362 | 1.243 | -1.211 | 0.630-1.420 | 0.946 | 0.795 | 0.208 | -0.056 | 0.033 | 0.377 |
| Intake of fat | 5 | 3.141 | 0.770-1.497 | 1.074 | 0.674 | 0.169 | 0.071 | 0.657-1.503 | 0.994 | 0.976 | 0.211 | -0.006 | 0.538-2.576 | 1.177 | 0.710 | 0.400 | 0.163 | 0.771-1.495 | 1.074 | 0.694 | 0.169 | 0.071 | 0.275 | 0.810 |
| Intake of carbohydrate | 10 | 29.697 | 0.544-1.016 | 0.743 | 0.062 | 0.159 | -0.297 | 0.616-1.549 | 0.976 | 0.919 | 0.235 | -0.024 | 0.009-1.026 | 0.097 | 0.088 | 1.205 | -2.336 | 0.512-1.126 | 0.759 | 0.201 | 0.201 | -0.275 | 0.119 | 0.124 |
| Breakfast skipping | 6 | 15.374 | 0.834-1.909 | 1.262 | 0.271 | 0.211 | 0.233 | 0.644-2.105 | 1.165 | 0.613 | 0.302 | 0.153 | 1.351-41.474 | 7.484 | 0.083 | 0.874 | 2.013 | 0.823-1.934 | 1.262 | 0.334 | 0.218 | 0.233 | 0.923 | 0.104 |
| Morning person | 115 | 19.939 | 0.993-1.117 | 1.053 | 0.083 | 0.030 | 0.052 | 0.950-1.118 | 1.031 | 0.466 | 0.042 | 0.030 | 0.878-1.280 | 1.060 | 0.544 | 0.096 | 0.059 | 1.000-1.121 | 1.058 | 0.054 | 0.029 | 0.057 | 0.032 | 0.943 |
| Insomnia | 38 | 32.624 | 1.873-3.986 | 2.733 | 1.809E-07 | 0.193 | 1.005 | 1.426-3.744 | 2.310 | 6.745E-04 | 0.246 | 0.837 | 0.734-9.439 | 2.632 | 0.146 | 0.652 | 0.968 | 1.958-4.031 | 2.809 | 1.828E-06 | 0.184 | 1.033 | 0.023 | 0.952 |
| Short sleep | 24 | 50.288 | 1.521-8.631 | 3.623 | 3.651E-03 | 0.443 | 1.287 | 0.814-6.086 | 2.226 | 0.119 | 0.513 | 0.800 | 0.707-2179.697 | 39.254 | 0.087 | 2.049 | 3.670 | 1.658-8.476 | 3.749 | 3.947E-03 | 0.416 | 1.322 | 0.002 | 0.247 |
| Long sleep | 8 | 29.657 | 0.119-2.498 | 0.545 | 0.434 | 0.777 | -0.608 | 0.045-2.078 | 0.306 | 0.226 | 0.977 | -1.183 | 5.729E-05-8.585 | 0.022 | 0.257 | 3.040 | -3.809 | 0.074-4.006 | 0.545 | 0.569 | 1.018 | -0.608 | 0.127 | 0.307 |
| Daytime sleepiness | 37 | 36.553 | 1.646-4.786 | 2.807 | 1.499E-04 | 0.272 | 1.032 | 1.252-4.325 | 2.327 | 7.547E-03 | 0.316 | 0.845 | 0.435-49.212 | 4.626 | 0.213 | 1.206 | 1.532 | 1.725-4.981 | 2.931 | 3.146E-04 | 0.271 | 1.075 | 0.011 | 0.673 |
| Daytime napping | 99 | 29.165 | 1.183-1.945 | 1.517 | 1.033E-03 | 0.127 | 0.417 | 1.326-2.566 | 1.845 | 2.762E-04 | 0.168 | 0.612 | 1.353-7.376 | 3.159 | 9.169E-03 | 0.433 | 1.150 | 1.166-1.908 | 1.492 | 1.937E-03 | 0.126 | 0.400 | 0.004 | 0.079 |
| Leisure sedentary behavior (TV watching) | 131 | 36.588 | 1.495-1.950 | 1.708 | 2.735E-15 | 0.068 | 0.535 | 1.361-1.896 | 1.606 | 1.978E-08 | 0.084 | 0.474 | 0.864-2.991 | 1.608 | 0.136 | 0.317 | 0.475 | 1.520-1.969 | 1.730 | 7.749E-14 | 0.066 | 0.548 | 2.341E-05 | 0.846 |

| | | | | | | | | | | | | | | | | | | | | | | | | |
|---|---|---|---|---|---|---|---|---|---|---|---|---|---|---|---|---|---|---|---|---|---|---|---|---|
| Leisure sedentary behavior (computer use) | 47 | 11.611 | 0.597-0.858 | 0.716 | 3.100E-04 | 0.093 | -0.334 | 0.558-0.942 | 0.725 | 0.016 | 0.134 | -0.322 | 0.079-0.982 | 0.278 | 0.053 | 0.644 | -1.280 | 0.587-0.873 | 0.716 | 1.817E-03 | 0.101 | -0.334 | 0.219 | 0.144 |
| Moderate to vigorous physical activity levels | 18 | 34.455 | 0.641-1.199 | 0.877 | 0.411 | 0.160 | -0.131 | 0.545-1.388 | 0.870 | 0.559 | 0.239 | -0.140 | 0.016-1.843 | 0.171 | 0.165 | 1.214 | -1.768 | 0.583-1.319 | 0.877 | 0.537 | 0.208 | -0.131 | 0.055 | 0.190 |
| 10+ minutes vigorous activity | 6 | 20.534 | 0.126-0.676 | 0.292 | 4.047E-03 | 0.428 | -1.229 | 0.095-1.095 | 0.323 | 0.070 | 0.623 | -1.131 | 1.777E-07-1.381 | 4.031E-04 | 0.133 | 4.153 | -7.816 | 0.088-0.975 | 0.292 | 0.046 | 0.614 | -1.229 | 0.178 | 0.185 |
| Strenuous sports or other exercises | 12 | 21.041 | 0.240-0.992 | 0.488 | 0.047 | 0.362 | -0.718 | 0.307-2.297 | 0.840 | 0.734 | 0.513 | -0.174 | 0.103-200.544 | 4.554 | 0.451 | 1.931 | 1.516 | 0.151-1.015 | 0.391 | 0.067 | 0.486 | -0.939 | 0.176 | 0.263 |
| **Health status** | | | | | | | | | | | | | | | | | | | | | | | | |
| Childhood-onset asthma | 99 | 21.940 | 0.999-1.034 | 1.018 | 0.068 | 9.743E-03 | 0.018 | 0.987-1.042 | 1.014 | 0.315 | 0.014 | 0.014 | 0.966-1.058 | 1.011 | 0.638 | 0.023 | 0.011 | 0.999-1.037 | 1.018 | 0.061 | 0.010 | 0.018 | 0.027 | 0.744 |
| Adult-onset asthma | 45 | 37.637 | 0.987-1.088 | 1.036 | 0.153 | 0.025 | 0.036 | 0.960-1.096 | 1.026 | 0.450 | 0.034 | 0.026 | 0.837-1.137 | 0.128 | 0.749 | 0.078 | -0.025 | 0.987-1.088 | 1.036 | 0.160 | 0.025 | 0.036 | 5.069E-03 | 0.416 |
| Type 2 diabetes | 345 | 21.254 | 1.002-1.043 | 1.023 | 0.030 | 0.010 | 0.022 | 0.988-1.054 | 1.021 | 0.214 | 0.017 | 0.021 | 0.960-1.040 | 0.999 | 0.978 | 0.021 | -5.755E-04 | 1.000-1.040 | 1.020 | 0.048 | 0.010 | 0.020 | 4.419E-04 | 0.197 |
| Coronary artery disease | 144 | 30.657 | 1.013-1.088 | 1.050 | 7.613E-03 | 0.018 | 0.049 | 1.009-1.112 | 1.060 | 0.019 | 0.025 | 0.058 | 0.943-1.089 | 1.013 | 0.717 | 0.037 | 0.013 | 1.013-1.088 | 1.050 | 8.493E-03 | 0.018 | 0.049 | 4.517E-06 | 0.231 |
| Atrial fibrillation | 111 | 15.710 | 0.991-1.042 | 1.016 | 0.216 | 0.013 | 0.016 | 0.970-1.062 | 1.015 | 0.520 | 0.023 | 0.015 | 0.948-1.057 | 1.001 | 0.969 | 0.028 | 1.077E-03 | 0.988-1.044 | 1.016 | 0.261 | 0.014 | 0.016 | 0.079 | 0.536 |
| Ulcerative colitis | 37 | 8.609 | 1.000-1.033 | 1.016 | 0.056 | 8.326E-03 | 0.016 | 0.977-1.027 | 1.002 | 0.903 | 0.013 | 1.575E-03 | 0.980-1.064 | 1.021 | 0.332 | 0.021 | 0.021 | 0.999-1.033 | 1.016 | 0.077 | 8.531E-03 | 0.016 | 0.280 | 0.802 |
| Crohn's disease | 51 | 47.280 | 0.987-1.024 | 1.006 | 0.542 | 9.362E-03 | 5.707E-03 | 0.974-1.020 | 0.997 | 0.778 | 0.012 | -3.352E-03 | 0.960-1.045 | 1.002 | 0.940 | 0.021 | 1.615E-03 | 0.988-1.023 | 1.005 | 0.553 | 9.026E-03 | 5.391E-03 | 9.384E-05 | 0.832 |
| **Nutritional & biochemical biomarkers** | | | | | | | | | | | | | | | | | | | | | | | | |
| Fasting glucose | 68 | 9.119 | 0.901-1.221 | 1.005 | 0.930 | 0.056 | 4.933E-03 | 0.909-1.287 | 1.081 | 0.378 | 0.089 | 0.078 | 0.856-1.284 | 1.048 | 0.651 | 0.104 | 0.047 | 0.901-1.119 | 1.009 | 0.865 | 0.053 | 9.003E-03 | 0.637 | 0.630 |
| Fating insulin | 37 | 16.784 | 0.904-1.346 | 1.103 | 0.332 | 0.102 | 0.098 | 0.838-1.500 | 1.121 | 0.441 | 0.149 | 0.114 | 0.474-1.782 | 0.919 | 0.805 | 0.338 | -0.084 | 0.886-1.374 | 1.103 | 0.385 | 0.112 | 0.098 | 0.159 | 0.570 |
| 2-hour blood glucose | 13 | 43.786 | 0.906-1.059 | 0.979 | 0.598 | 0.040 | -0.021 | 0.896-1.112 | 0.998 | 0.977 | 0.055 | -1.590E-03 | 0.673-1.024 | 0.831 | 0.511 | 0.107 | -0.186 | 0.909-1.055 | 0.979 | 0.594 | 0.038 | -0.021 | 0.682 | 0.126 |
| HbA1c | 72 | 3.792 | 0.863-1.159 | 1.000 | 0.999 | 0.075 | -2.129E-05 | 0.803-1.298 | 1.021 | 0.868 | 0.123 | 0.020 | 0.841-1.442 | 1.101 | 0.485 | 0.137 | 0.096 | 0.866-1.155 | 1.000 | 0.995 | 0.073 | 4.583E-04 | 0.532 | 0.405 |
| HDL cholesterol | 136 | 19.853 | 0.805-1.008 | 0.901 | 0.070 | 0.057 | -0.104 | 0.830-1.165 | 0.984 | 0.848 | 0.087 | -0.017 | 0.813-1.241 | 1.004 | 0.967 | 0.108 | 4.440E-03 | 0.807-1.006 | 0.901 | 0.067 | 0.056 | -0.104 | 0.023 | 0.237 |
| LDL cholesterol | 110 | 15.158 | 0.866-1.025 | 0.942 | 0.163 | 0.043 | -0.060 | 0.819-1.046 | 0.925 | 0.214 | 0.062 | -0.078 | 0.795-1.065 | 0.920 | 0.266 | 0.075 | -0.084 | 0.863-1.036 | 0.945 | 0.229 | 0.047 | -0.056 | 0.087 | 0.684 |
| Total cholesterol | 130 | 10.860 | 0.895-1.059 | 0.974 | 0.537 | 0.043 | -0.027 | 0.854-1.108 | 0.973 | 0.678 | 0.066 | -0.028 | 0.797-1.067 | 0.922 | 0.278 | 0.074 | -0.081 | 0.907-1.081 | 0.990 | 0.825 | 0.045 | -9.952E-03 | 0.148 | 0.354 |
| Triglycerides | 115 | 32.948 | 0.834-1.064 | 0.942 | 0.336 | 0.062 | -0.060 | 0.750-1.049 | 0.887 | 0.163 | 0.086 | -0.120 | 0.694-1.049 | 0.853 | 0.134 | 0.106 | -0.159 | 0.843-1.071 | 0.950 | 0.403 | 0.061 | -0.051 | 4.227E-04 | 0.246 |
| Adiponectin | 7 | 61.395 | 0.886-1.571 | 1.180 | 0.258 | 0.146 | 0.165 | 0.848-1.431 | 1.102 | 0.469 | 0.134 | 0.097 | 0.501-2.537 | 1.127 | 0.785 | 0.414 | 0.119 | 0.886-1.571 | 1.180 | 0.301 | 0.146 | 0.165 | 8.281E-03 | 0.909 |
| Leptin | 5 | 15.136 | 0.678-1.162 | 0.887 | 0.385 | 0.138 | -0.120 | 0.595-1.231 | 0.856 | 0.401 | 0.185 | -0.156 | 6.132E-04-17.527 | 0.104 | 0.45 | 2.617 | -2.267 | 0.871-1.148 | 0.871 | 0.373 | 0.141 | -0.138 | 0.194 | 0.471 |

| | | | | | | | | | | | | | | | | | | | | | | | | |
|---|---|---|---|---|---|---|---|---|---|---|---|---|---|---|---|---|---|---|---|---|---|---|---|---|
| Vitamin C | 10 | 22.448 | 0.891-1.064 | 0.974 | 0.555 | 0.045 | -0.027 | 0.858-1.066 | 0.956 | 0.419 | 0.055 | -0.045 | 0.797-1.115 | 0.943 | 0.510 | 0.086 | -0.059 | 0.888-1.081 | 0.980 | 0.697 | 0.050 | -0.020 | 0.170 | 0.638 |
| 25-Hydroxyvitamin D | 116 | 18.292 | 0.939-1.107 | 1.019 | 0.647 | 0.042 | 0.019 | 0.882-1.133 | 0.999 | 0.993 | 0.064 | -5.953E-04 | 0.817-1.062 | 0.931 | 0.289 | 0.067 | -0.071 | 0.942-1.111 | 1.023 | 0.589 | 0.042 | 0.022 | 0.045 | 0.087 |
| **Nutritional & developmental status** | | | | | | | | | | | | | | | | | | | | | | | | |
| Body mass index | 436 | 28.637 | 1.361-1.543 | 1.449 | 5.108E-31 | 0.032 | 0.371 | 1.235-1.466 | 1.346 | 1.310E-11 | 0.044 | 0.297 | 0.936-1.325 | 1.113 | 0.226 | 0.089 | 0.107 | 1.386-1.508 | 1.447 | 1.791E-28 | 0.031 | 0.369 | 4.697E-08 | 0.102 |
| Body fat percentage | 378 | 30.422 | 1.450-1.735 | 1.586 | 5.999E-24 | 0.046 | 0.461 | 1.281-1.631 | 1.446 | 1.991E-09 | 0.062 | 0.369 | 1.098-1.964 | 1.468 | 0.010 | 0.148 | 0.384 | 1.435-1.714 | 1.568 | 8.505E-21 | 0.045 | 0.450 | 4.207E-08 | 0.584 |
| Whole body fat mass | 418 | 27.834 | 1.279-1.456 | 1.365 | 4.366E-21 | 0.033 | 0.311 | 1.179-1.410 | 1.289 | 2.770E-08 | 0.046 | 0.254 | 0.959-1.388 | 1.154 | 0.131 | 0.094 | 0.143 | 1.281-1.455 | 1.365 | 1.047E-19 | 0.033 | 0.311 | 2.376E-07 | 0.058 |
| Whole body fat-free mass | 532 | 27.579 | 0.879-1.025 | 0.949 | 0.180 | 0.039 | -0.053 | 0.860-1.060 | 0.955 | 0.384 | 0.053 | -0.046 | 0.700-1.010 | 0.841 | 0.065 | 0.094 | -0.173 | 0.884-1.028 | 0.953 | 0.215 | 0.039 | -0.048 | 1.040E-08 | 0.156 |
| Visceral adipose tissue | 184 | 31.705 | 1.287-1.513 | 1.395 | 7.476E-16 | 0.041 | 0.333 | 1.207-1.488 | 1.340 | 3.990E-08 | 0.053 | 0.293 | 0.846-1.407 | 1.091 | 0.501 | 0.130 | 0.087 | 1.275-1.490 | 1.378 | 5.230E-14 | 0.040 | 0.321 | 3.479E-05 | 0.097 |
| Waist circumference | 356 | 40.154 | 1.372-1.635 | 1.498 | 1.073E-19 | 0.045 | 0.404 | 1.257-1.572 | 1.406 | 2.552E-09 | 0.057 | 0.341 | 0.984-1.647 | 1.273 | 0.067 | 0.131 | 0.241 | 1.370-1.627 | 1.493 | 4.284E-18 | 0.044 | 0.401 | 2.401E-14 | 0.189 |
| Hip circumference | 396 | 24.992 | 1.119-1.270 | 1.192 | 5.563E-08 | 0.032 | 0.176 | 1.007-1.212 | 1.105 | 0.035 | 0.047 | 0.100 | 0.794-1.138 | 0.950 | 0.580 | 0.092 | -0.051 | 1.114-1.262 | 1.186 | 1.487E-07 | 0.032 | 0.170 | 8.469E-06 | 0.129 |
| Waist-to-hip ratio | 338 | 29.275 | 1.272-1.489 | 1.376 | 2.400E-15 | 0.040 | 0.319 | 1.201-1.499 | 1.342 | 1.872E-07 | 0.056 | 0.294 | 0.815-1.249 | 1.009 | 0.933 | 0.109 | 9.170E-03 | 1.270-1.483 | 1.372 | 2.048E-14 | 0.040 | 0.316 | 6.738E-07 | 0.235 |
| Standing height | 742 | 22.633 | 0.860-0.943 | 0.900 | 9.371E-06 | 0.024 | -0.105 | 0.895-1.029 | 0.960 | 0.244 | 0.035 | -0.041 | 0.887-1.068 | 0.973 | 0.562 | 0.047 | -0.027 | 0.859-0.941 | 0.899 | 5.666E-06 | 0.023 | -0.106 | 9.875E-08 | 0.060 |
| Sitting height | 576 | 22.569 | 0.848-0.944 | 0.895 | 4.387E-05 | 0.027 | -0.111 | 0.853-0.996 | 0.922 | 0.040 | 0.040 | -0.082 | 0.815-1.038 | 0.920 | 0.176 | 0.062 | -0.084 | 0.853-0.948 | 0.899 | 8.006E-05 | 0.027 | -0.106 | 2.354E-06 | 0.621 |
| Hand grip strength (left) | 153 | 18.480 | 0.665-0.897 | 0.772 | 7.408E-04 | 0.077 | -0.258 | 0.585-0.888 | 0.721 | 2.069E-03 | 0.106 | -0.327 | 0.426-1.331 | 0.753 | 0.331 | 0.291 | -0.283 | 0.663-0.893 | 0.769 | 7.161E-04 | 0.076 | -0.263 | 0.026 | 0.929 |
| Hand grip strength (right) | 168 | 30.497 | 0.656-0.894 | 0.766 | 7.150E-04 | 0.079 | -0.267 | 0.590-0.877 | 0.719 | 1.139E-03 | 0.101 | -0.330 | 0.342-1.025 | 0.592 | 0.063 | 0.280 | -0.524 | 0.654-0.883 | 0.759 | 4.277E-04 | 0.077 | -0.275 | 1.442E-04 | 0.340 |
| Birth weight | 170 | 25.119 | 0.816-0.958 | 0.884 | 2.569E-03 | 0.041 | -0.123 | 0.765-0.957 | 0.856 | 6.522E-03 | 0.057 | -0.156 | 0.660-1.013 | 0.817 | 0.067 | 0.109 | -0.202 | 0.825-0.966 | 0.893 | 5.249E-03 | 0.040 | -0.113 | 1.982E-03 | 0.439 |
| Childhood BMI | 18 | 50.203 | 0.973-1.050 | 1.011 | 0.575 | 0.019 | 0.011 | 0.973-1.029 | 1.001 | 0.952 | 0.014 | 8.556E-04 | 0.960-1.044 | 1.001 | 0.957 | 0.021 | 1.172E-03 | 0.973-1.050 | 1.011 | 0.582 | 0.019 | 0.011 | 5.204E-03 | 0.319 |
| **Emotional factors** | | | | | | | | | | | | | | | | | | | | | | | | |
| Major depressive disorder | 47 | 0.242 | 1.414-1.702 | 1.551 | 1.802E-20 | 0.047 | 0.439 | 1.318-1.739 | 1.514 | 4.761E-09 | 0.071 | 0.414 | 1.638-4.404 | 2.686 | 3.027E-04 | 0.252 | 0.988 | 1.400-1.695 | 1.541 | 9.838E-12 | 0.049 | 0.432 | 0.435 | 0.092 |
| Anxious feeling | 22 | 39.022 | 1.340-2.750 | 1.920 | 3.804E-04 | 0.184 | 0.652 | 1.596-3.432 | 2.341 | 1.327E-05 | 0.195 | 0.850 | 1.324-215.480 | 16.891 | 0.042 | 1.299 | 2.827 | 1.340-2.750 | 1.920 | 1.880E-03 | 0.184 | 0.652 | 0.023 | 0.107 |
| Schizophrenia | 189 | 21.293 | 0.984-1.038 | 1.010 | 0.442 | 0.014 | 0.010 | 0.977-1.052 | 1.014 | 0.467 | 0.019 | 0.014 | 0.892-1.153 | 1.014 | 0.830 | 0.065 | 0.014 | 0.983-1.035 | 1.008 | 0.523 | 0.013 | 8.457E-03 | 6.570E-03 | 0.469 |
| Bipolar disorder | 53 | 22.702 | 0.999-1.002 | 1.000 | 0.639 | 8.277E-04 | 3.880E-04 | 0.997-1.001 | 0.999 | 0.594 | 1.282E-03 | -6.842E-04 | 0.999-1.017 | 1.008 | 0.105 | 4.756E-03 | 7.850E-03 | 0.999-1.002 | 1.001 | 0.586 | 9.590E-04 | 5.252E-04 | 0.063 | 0.115 |
| Anorexia nervosa | 6 | 170.613 | 0.855-1.011 | 0.930 | 0.090 | 0.043 | -0.073 | 0.866-1.063 | 0.960 | 0.429 | 0.052 | -0.041 | 0.652-1.245 | 0.901 | 0.561 | 0.165 | -0.105 | 0.898-0.982 | 0.939 | 0.028 | 0.023 | -0.063 | 0.764 | 0.851 |
| Life satisfaction | 75 | 36.327 | 0.284-0.515 | 0.382 | 2.510E-10 | 0.152 | -0.962 | 0.295-0.637 | 0.434 | 2.063E-05 | 0.196 | -0.835 | 0.087-1.949 | 0.667 | 0.267 | 0.793 | -0.888 | 0.282-0.499 | 0.375 | 2.354E-09 | 0.145 | -0.980 | 9.741E-04 | 0.924 |
| Positive affect | 90 | 25.992 | 0.313-0.530 | 0.407 | 2.140E-11 | 0.134 | -0.899 | 0.284-0.597 | 0.412 | 2.880E-06 | 0.189 | -0.886 | 0.342-3.113 | 1.032 | 0.956 | 0.563 | 0.031 | 0.323-0.538 | 0.416 | 1.391E-09 | 0.130 | -0.876 | 0.013 | 0.093 |
| **Socioeconomic factors** | | | | | | | | | | | | | | | | | | | | | | | | |

| | | | | | | | | | | | | | | | | | | | | | | | | | |
|---|---|---|---|---|---|---|---|---|---|---|---|---|---|---|---|---|---|---|---|---|---|---|---|---|---|
| Income | 43 | 27.953 | 0.480-0.692 | 0.577 | 3.274E-09 | 0.093 | -0.551 | 0.446-0.719 | 0.566 | 3.026E-06 | 0.122 | -0.569 | 0.333-1.574 | 0.724 | 0.420 | 0.396 | -0.322 | 0.493-0.696 | 0.586 | 2.094E-07 | 0.088 | -0.535 | 0.039 | 0.557 |
| Educational attainment | 1207 | 15.067 | 0.474-0.530 | 0.501 | 4.688E-131 | 0.028 | -0.691 | 0.482-0.564 | 0.522 | 6.906E-59 | 0.040 | -0.651 | 0.439-0.647 | 0.533 | 2.720E-10 | 0.099 | -0.630 | 0.480-0.536 | 0.507 | 2.475E-107 | 0.028 | -0.679 | 1.621E-05 | 0.519 |
| Intelligence | 166 | 35.029 | 0.622-0.748 | 0.682 | 3.165E-16 | 0.047 | -0.383 | 0.606-0.768 | 0.683 | 2.418E-10 | 0.060 | -0.382 | 0.365-0.862 | 0.561 | 9.223E-03 | 0.219 | -0.578 | 0.622-0.748 | 0.682 | 6.838E-14 | 0.047 | -0.383 | 8.302E-06 | 0.363 |

SNP, single nucleotide polymorphism; OR, odds ratio; CI, confidence interval; SE, standard error

[a]*I^2* was utilized to quantify the heterogeneity among SNPs for each exposure.

[b]*P* value for heterogeneity was the *P* value of Cochran's *Q* test. *P* value $\leq$ 0.05 was deemed as a statistically significant heterogeneity.

[c]*P* value for pleiotropy was the *P* value of MR-Egger intercept test. *P* value $\leq$ 0.05 was deemed as a statistically significant horizontal pleiotropy

**Table S5. The summary of the MR results in the replication phase**

| Exposures | Used SNPs | I^2(%)[a] | IVW method | | | | | Weighted median method | | | | | MR-Egger method | | | | | MR PRESSO model | | | | | *P* value for heterogeneity[b] | *P* value for pleiotropy[c] |
|---|---|---|---|---|---|---|---|---|---|---|---|---|---|---|---|---|---|---|---|---|---|---|---|---|
| | | | 95%CI | OR | *P* value | SE | BETA | 95%CI | OR | *P* value | SE | BETA | 95%CI | OR | *P* value | SE | BETA | 95%CI | OR | *P* value | SE | BETA | | |
| **Daily habits** | | | | | | | | | | | | | | | | | | | | | | | | |
| Alcohol consumption | 90 | 24.689 | 0.802-1.521 | 1.104 | 0.544 | 0.163 | 0.099 | 0.783-1.953 | 1.236 | 0.363 | 0.233 | 0.212 | 0.344-1.621 | 0.747 | 0.462 | 0.395 | -0.292 | 0.775-1.463 | 1.065 | 0.700 | 0.162 | 0.063 | 0.018 | 0.280 |
| Coffee consumption | 27 | 35.635 | 0.598-1.458 | 0.934 | 0.763 | 0.227 | -0.068 | 0.647-1.984 | 1.133 | 0.662 | 0.286 | 0.125 | 0.500-2.895 | 1.203 | 0.684 | 0.448 | 0.185 | 0.598-1.458 | 0.934 | 0.766 | 0.227 | -0.068 | 0.027 | 0.516 |
| Caffeine consumption | 26 | 44.392 | 0.583-1.168 | 0.825 | 0.279 | 0.177 | -0.192 | 0.710-1.560 | 1.053 | 0.798 | 0.201 | 0.051 | 0.663-2.633 | 1.321 | 0.437 | 0.352 | 0.278 | 0.583-1.168 | 0.825 | 0.289 | 0.177 | -0.192 | 5.894E-03 | 0.139 |
| Smoking initiation | 334 | 17.250 | 1.129-1.365 | 1.241 | 8.065E-06 | 0.048 | 0.216 | 1.072-1.401 | 1.226 | 2.864E-03 | 0.068 | 0.203 | 1.006-1.511 | 1.233 | 0.022 | 0.104 | 0.210 | 1.114-1.344 | 1.224 | 3.014E-05 | 0.048 | 0.202 | 4.844E-03 | 0.029 |
| Lifetime smoking | 117 | 15.737 | 1.111-1.903 | 1.454 | 6.360E-03 | 0.137 | 0.374 | 1.057-2.497 | 1.625 | 0.027 | 0.219 | 0.485 | 0.144-1.651 | 0.488 | 0.251 | 0.622 | -0.717 | 1.072-1.8985 | 1.425 | 0.016 | 0.145 | 0.354 | 0.074 | 0.073 |
| Cigarette consumption | 51 | 23.305 | 1.031-1.420 | 1.210 | 0.020 | 0.082 | 0.191 | 0.892-1.501 | 1.157 | 0.273 | 0.133 | 0.146 | 0.748-1.449 | 1.041 | 0.811 | 0.169 | 0.040 | 1.000-1.442 | 1.201 | 0.055 | 0.093 | 0.183 | 0.061 | 0.288 |
| Age of initiation of smoking | 8 | 313.903 | 0.490-1.670 | 0.904 | 0.748 | 0.313 | -0.101 | 0.373-1.932 | 0.849 | 0.697 | 0.419 | -0.164 | 1.599-709.447 | 33.686 | 0.064 | 1.555 | 3.517 | 0.420-1.662 | 0.836 | 0.621 | 0.351 | -0.180 | 0.946 | 0.055 |
| Smoking cessation | 21 | 6.966 | 0.941-1.430 | 1.160 | 0.165 | 0.107 | 0.148 | 0.752-1.406 | 1.028 | 0.862 | 0.160 | 0.028 | 0.454-1.452 | 0.812 | 0.492 | 0.297 | -0.208 | 0.925-1.455 | 1.160 | 0.214 | 0.116 | 0.148 | 0.310 | 0.209 |
| Intake of total sugar | 9 | 1.583 | 0.656-1.541 | 1.005 | 0.980 | 0.218 | 5.357E-03 | 0.582-1.809 | 1.026 | 0.928 | 0.289 | 0.026 | 0.113-27.523 | 1.763 | 0.698 | 1.402 | 0.567 | 0.650-1.554 | 1.005 | 0.981 | 0.222 | 5.36E-03 | 0.321 | 0.697 |
| Intake of fat | 5 | 118.714 | 0.785-2.602 | 1.429 | 0.243 | 0.306 | 0.357 | 0.574-2.659 | 1.235 | 0.589 | 0.391 | 0.211 | 0.106-3.418 | 0.602 | 0.607 | 0.886 | -0.507 | 0.857-2.382 | 1.429 | 0.243 | 0.261 | 0.357 | 0.607 | 0.375 |
| Intake of carbohydrate | 9 | 5.306 | 0.637-1.723 | 1.047 | 0.855 | 0.254 | 0.046 | 0.482-1.873 | 0.950 | 0.882 | 0.346 | -0.051 | 0.025-14.435 | 0.602 | 0.763 | 1.621 | -0.507 | 0.705-1.943 | 1.170 | 0.558 | 0.259 | 0.157 | 0.295 | 0.739 |
| Breakfast skipping | 6 | 58.474 | 0.993-1.027 | 1.010 | 0.245 | 8.580E-03 | 9.972E-03 | 0.994-1.038 | 1.016 | 0.151 | 0.011 | 0.016 | 0.973-1.118 | 1.043 | 0.304 | 0.035 | 0.042 | 0.995-1.025 | 1.010 | 0.251 | 7.687E-03 | 9.972E-03 | 0.532 | 0.407 |
| Morning person | 113 | 18.844 | 0.898-1.077 | 0.983 | 0.719 | 0.046 | -0.017 | 0.885-1.124 | 0.998 | 0.969 | 0.061 | -2.341E-03 | 0.756-1.333 | 1.004 | 0.979 | 0.145 | 3.904E-03 | 0.904-1.077 | 0.987 | 0.765 | 0.045 | -0.013 | 0.042 | 0.881 |
| Insomnia | 36 | 20.214 | 1.215-3.153 | 1.958 | 5.748E-03 | 0.243 | 0.672 | 0.871-3.736 | 1.804 | 0.112 | 0.372 | 0.590 | 0.200-5.452 | 1.046 | 0.958 | 0.843 | 0.045 | 1.297-3.845 | 2.233 | 6.292E-03 | 0.277 | 0.803 | 0.120 | 0.436 |
| Short sleep | 24 | 42.630 | 1.632-5.153 | 2.900 | 6.927E-03 | 0.293 | 1.065 | 1.838-37.090 | 8.258 | 5.878E-03 | 0.766 | 2.111 | 0.017-35753.988 | 24.580 | 0.398 | 3.716 | 3.202 | 1.895-6.633 | 3.545 | 3.980E-03 | 0.320 | 1.266 | 0.011 | 0.698 |
| Long sleep | 8 | 62.520 | 0.741-2.540 | 1.640 | 0.808 | 0.459 | 0.495 | 0.065-67.602 | 2.092 | 0.677 | 1.773 | 0.738 | 5.736E-06-5995004.000 | 5.864 | 0.811 | 7.060 | 1.769 | 0.030-88.530 | 1.640 | 0.815 | 2.035 | 0.495 | 4.746E-03 | 0.856 |
| Daytime sleepiness | 36 | 28.073 | 0.486-2.458 | 1.093 | 0.829 | 0.413 | 0.089 | 0.237-1.800 | 0.653 | 0.410 | 0.517 | -0.426 | 0.010-12.781 | 0.366 | 0.583 | 1.813 | -1.006 | 0.518-2.551 | 1.149 | 0.734 | 0.407 | 0.139 | 0.049 | 0.539 |
| Daytime napping | 91 | 28.117 | 0.804-1.781 | 1.197 | 0.375 | 0.203 | 0.180 | 0.755-2.119 | 1.265 | 0.373 | 0.263 | 0.235 | 0.185-3.248 | 0.776 | 0.729 | 0.730 | -0.254 | 0.782-1.715 | 1.158 | 0.466 | 0.200 | 0.147 | 6.898E-03 | 0.538 |
| Leisure sedentary behavior (TV watching) | 113 | 16.359 | 1.225-1.739 | 1.460 | 2.299E-05 | 0.089 | 0.378 | 0.971-1.633 | 1.259 | 0.082 | 0.133 | 0.230 | 0.282-1.696 | 0.692 | 0.423 | 0.457 | -0.368 | 1.231-1.773 | 1.477 | 5.141E-05 | 0.093 | 0.390 | 0.069 | 0.098 |
| Leisure sedentary behavior (computer use) | 45 | 4.217 | 0.549-0.977 | 0.732 | 0.034 | 0.147 | -0.311 | 0.461-1.066 | 0.701 | 0.097 | 0.214 | -0.355 | 0.035-1.705 | 0.245 | 0.163 | 0.989 | -1.406 | 0.543-0.988 | 0.732 | 0.047 | 0.153 | -0.311 | 0.351 | 0.269 |
| Moderate to vigorous physical activity levels | 18 | 18.694 | 0.546-1.404 | 0.876 | 0.582 | 0.241 | -0.133 | 0.421-1.537 | 0.805 | 0.511 | 0.330 | -0.217 | 0.022-4.538 | 0.313 | 0.407 | 1.364 | -1.162 | 0.563-1.362 | 0.879 | 0.564 | 0.225 | -0.133 | 0.575 | 0.455 |
| 10+ minutes vigorous activity | 7 | 68.470 | 0.314-3.220 | 1.006 | 0.992 | 0.593 | 6.144E-03 | 0.376-7.473 | 1.677 | 0.498 | 0.762 | 0.517 | 2.111E-03-137142.000 | 17.017 | 0.564 | 4.589 | 2.834 | 0.392-2.585 | 1.006 | 0.990 | 0.481 | 6.144E-03 | 0.614 | 0.562 |
| Strenuous sports or other exercises | 12 | 11.973 | 0.039-0.368 | 0.120 | 2.078E-04 | 0.571 | -2.119 | 0.032-0.735 | 0.154 | 0.019 | 0.796 | -1.868 | 7.017E-04-30.010 | 0.145 | 0.494 | 2.720 | -1.930 | 0.050-0.409 | 0.143 | 3.477E-03 | 0.536 | -1.944 | 0.456 | 0.945 |
| **Health status** | | | | | | | | | | | | | | | | | | | | | | | | |
| Childhood-onset asthma | 90 | 13.859 | 0.970-1.026 | 0.998 | 0.878 | 0.014 | -2.211E-03 | 0.956-1.048 | 1.001 | 0.968 | 0.024 | 9.398E-04 | 0.909-1.055 | 0.979 | 0.586 | 0.038 | -0.021 | 0.968-1.029 | 0.998 | 0.907 | 0.015 | -1.810E-03 | 0.126 | 0.593 |
| Adult-onset asthma | 37 | 3.222 | 1.013-1.163 | 1.086 | 0.020 | 0.035 | 0.082 | 0.993-1.220 | 1.101 | 0.067 | 0.052 | 0.096 | 0.739-1.238 | 0.957 | 0.739 | 0.131 | -0.044 | 1.011-1.166 | 1.086 | 0.030 | 0.036 | 0.082 | 0.368 | 0.325 |
| Type 2 diabetes | 324 | 16.876 | 1.013-1.078 | 1.045 | 5.702E-03 | 0.016 | 0.044 | 0.986-1.083 | 1.034 | 0.168 | 0.024 | 0.033 | 0.965-1.095 | 1.028 | 0.399 | 0.032 | 0.027 | 1.011-1.072 | 1.041 | 8.421E-03 | 0.015 | 0.040 | 6.429E-03 | 0.552 |
| Coronary artery disease | 137 | 11.772 | 0.963-1.060 | 1.011 | 0.664 | 0.024 | 0.011 | 0.929-1.101 | 1.011 | 0.798 | 0.043 | 0.011 | 0.943-1.170 | 1.051 | 0.372 | 0.055 | 0.049 | 0.960-1.064 | 1.011 | 0.684 | 0.026 | 0.011 | 0.124 | 0.427 |

| | | | | | | | | | | | | | | | | | | | | | | | | |
|---|---|---|---|---|---|---|---|---|---|---|---|---|---|---|---|---|---|---|---|---|---|---|---|---|
| Atrial fibrillation | 107 | 1.304 | 0.979-1.057 | 1.017 | 0.387 | 0.020 | 0.017 | 0.981-1.109 | 1.043 | 0.180 | 0.031 | 0.042 | 0.967-1.122 | 1.042 | 0.286 | 0.038 | 0.041 | 0.977-1.056 | 1.015 | 0.443 | 0.020 | 0.015 | 0.417 | 0.466 |
| Ulcerative colitis | 34 | 4.197 | 1.010-1.071 | 1.040 | 7.931E-03 | 0.015 | 0.039 | 0.998-1.085 | 1.040 | 0.065 | 0.021 | 0.040 | 0.937-1.139 | 1.033 | 0.514 | 0.050 | 0.033 | 1.012-1.070 | 1.041 | 7.876E-03 | 0.014 | 0.040 | 0.483 | 0.894 |
| Crohn's disease | 48 | 46.961 | 0.987-1.032 | 1.009 | 0.412 | 0.011 | 9.361E-03 | 0.968-1.033 | 1.000 | 0.994 | 0.017 | 1.193E-04 | 0.944-1.054 | 0.997 | 0.921 | 0.028 | -2.800E-03 | 0.989-1.026 | 1.007 | 0.426 | 9.132E-03 | 7.333E-03 | 0.942 | 0.638 |
| **Nutritional & biochemical biomarkers** | | | | | | | | | | | | | | | | | | | | | | | | |
| Fasting glucose | 65 | 17.783 | 0.928-1.303 | 1.100 | 0.272 | 0.087 | 0.095 | 0.896-1.661 | 1.220 | 0.206 | 0.157 | 0.199 | 0.963-1.925 | 1.361 | 0.086 | 0.177 | 0.308 | 0.912-1.320 | 1.097 | 0.328 | 0.094 | 0.093 | 0.099 | 0.155 |
| Fating insulin | 38 | 23.908 | 0.735-1.379 | 1.007 | 0.964 | 0.160 | 7.170E-03 | 0.537-1.423 | 0.874 | 0.588 | 0.248 | -0.135 | 0.151-1.555 | 0.484 | 0.231 | 0.595 | -0.726 | 0.696-1.457 | 1.007 | 0.970 | 0.188 | 7.170E-03 | 0.078 | 0.203 |
| 2-hour blood glucose | 11 | 26.433 | 0.871-1.125 | 0.990 | 0.876 | 0.065 | -0.010 | 0.791-1.177 | 0.965 | 0.724 | 0.102 | -0.036 | 0.799-1.920 | 1.239 | 0.363 | 0.223 | 0.214 | 0.849-1.144 | 0.986 | 0.854 | 0.076 | -0.014 | 0.138 | 0.310 |
| HbA1c | 69 | 10.439 | 0.882-1.437 | 1.126 | 0.340 | 0.124 | 0.119 | 0.870-1.865 | 1.274 | 0.213 | 0.195 | 0.242 | 0.660-1.708 | 1.062 | 0.804 | 0.242 | 0.060 | 0.896-1.420 | 1.128 | 0.308 | 0.117 | 0.120 | 0.664 | 0.780 |
| HDL cholesterol | 103 | 22.269 | 0.735-1.045 | 0.877 | 0.143 | 0.090 | -0.132 | 0.652-1.061 | 0.832 | 0.138 | 0.124 | -0.184 | 0.696-1.330 | 0.962 | 0.815 | 0.165 | -0.039 | 0.732-1.042 | 0.874 | 0.136 | 0.090 | -0.135 | 0.013 | 0.504 |
| LDL cholesterol | 104 | 19.188 | 0.841-1.119 | 0.971 | 0.681 | 0.073 | -0.030 | 0.791-1.175 | 0.964 | 0.717 | 0.101 | -0.037 | 0.972-1.482 | 1.200 | 0.092 | 0.108 | 0.183 | 0.844-1.124 | 0.974 | 0.722 | 0.073 | -0.026 | 0.045 | 9.911E-03 |
| Total cholesterol | 121 | 11.679 | 0.902-1.166 | 1.026 | 0.697 | 0.066 | 0.025 | 0.887-1.363 | 1.010 | 0.386 | 0.109 | 0.095 | 1.001-1.563 | 1.251 | 0.051 | 0.114 | 0.224 | 0.909-1.195 | 1.042 | 0.556 | 0.070 | 0.041 | 0.138 | 0.029 |
| Triglycerides | 114 | 10.494 | 0.910-1.227 | 1.057 | 0.471 | 0.076 | 0.055 | 0.911-1.507 | 1.171 | 0.218 | 0.128 | 0.158 | 0.883-1.479 | 1.143 | 0.312 | 0.132 | 0.134 | 0.911-1.241 | 1.063 | 0.437 | 0.079 | 0.061 | 0.169 | 0.451 |
| Adiponectin | 10 | 155.875 | 0.924-1.442 | 1.154 | 0.207 | 0.114 | 0.143 | 0.814-1.453 | 1.087 | 0.571 | 0.148 | 0.084 | 0.484-1.618 | 0.885 | 0.702 | 0.308 | -0.122 | 0.988-1.348 | 1.154 | 0.104 | 0.079 | 0.143 | 0.898 | 0.380 |
| Leptin | 5 | 45.520 | 0.280-1.224 | 0.586 | 0.155 | 0.376 | -0.535 | 0.322-1.322 | 0.653 | 0.237 | 0.360 | -0.426 | 2.942E-08-114.703 | 1.837E-03 | 0.345 | 5.634 | -6.300 | 0.318-1.097 | 0.590 | 0.156 | 0.316 | -0.527 | 0.027 | 0.381 |
| Vitamin C | 7 | 14.059 | 0.864-1.368 | 1.087 | 0.477 | 0.117 | 0.083 | 0.952-1.720 | 1.280 | 0.102 | 0.151 | 0.247 | 0.987-6.430 | 2.519 | 0.111 | 0.478 | 0.924 | 0.870-1.452 | 1.124 | 0.400 | 0.131 | 0.117 | 0.385 | 0.129 |
| 25-Hydroxyvitamin D | 109 | 1.401 | 0.923-1.169 | 1.039 | 0.525 | 0.060 | 0.038 | 0.788-1.139 | 0.947 | 0.567 | 0.094 | -0.054 | 0.777-1.161 | 0.950 | 0.617 | 0.103 | -0.052 | 0.925-1.175 | 1.043 | 0.493 | 0.061 | 0.042 | 0.414 | 0.279 |
| **Nutritional & developmental status** | | | | | | | | | | | | | | | | | | | | | | | | |
| Body mass index | 416 | 12.345 | 1.144-1.366 | 1.251 | 8.122E-07 | 0.045 | 0.224 | 1.103-1.518 | 1.294 | 1.549E-03 | 0.081 | 0.258 | 0.836-1.345 | 1.061 | 0.628 | 0.121 | 0.059 | 1.130-1.348 | 1.234 | 3.743E-06 | 0.045 | 0.211 | 0.023 | 0.144 |
| Body fat percentage | 363 | 20.137 | 1.097-1.431 | 1.253 | 8.808E-04 | 0.068 | 0.226 | 0.962-1.416 | 1.168 | 0.116 | 0.099 | 0.155 | 0.689-1.633 | 1.061 | 0.790 | 0.220 | 0.059 | 1.093-1.419 | 1.245 | 1.042E-03 | 0.066 | 0.219 | 6.799E-04 | 0.426 |
| Whole body fat mass | 403 | 19.041 | 1.081-1.308 | 1.189 | 3.672E-04 | 0.049 | 0.173 | 0.982-1.309 | 1.134 | 0.086 | 0.073 | 0.126 | 0.926-1.579 | 1.209 | 0.164 | 0.136 | 0.190 | 1.083-1.304 | 1.188 | 3.182E-04 | 0.048 | 0.172 | 7.858E-04 | 0.896 |
| Whole body fat-free mass | 509 | 5.416 | 0.785-0.964 | 0.870 | 8.012E-03 | 0.052 | -0.139 | 0.675-0.954 | 0.803 | 0.013 | 0.088 | -0.220 | 0.573-0.940 | 0.734 | 0.015 | 0.126 | -0.309 | 0.777-0.957 | 0.862 | 5.609E-03 | 0.053 | -0.148 | 0.172 | 0.137 |
| Visceral adipose tissue | 175 | 23.030 | 1.051-1.335 | 1.184 | 5.449E-03 | 0.061 | 0.169 | 0.972-1.404 | 1.168 | 0.098 | 0.094 | 0.156 | 0.738-1.548 | 1.069 | 0.726 | 0.189 | 0.066 | 1.051-1.322 | 1.179 | 5.401E-03 | 0.058 | 0.164 | 4.156E-03 | 0.566 |
| Waist circumference | 341 | 13.772 | 1.107-1.388 | 1.240 | 1.957E-04 | 0.058 | 0.215 | 1.084-1.590 | 1.313 | 5.383E-03 | 0.098 | 0.272 | 0.782-1.482 | 1.076 | 0.653 | 0.163 | 0.073 | 1.095-1.368 | 1.224 | 4.143E-03 | 0.057 | 0.202 | 0.020 | 0.355 |
| Hip circumference | 387 | 17.037 | 0.923-1.110 | 1.012 | 0.803 | 0.047 | 0.012 | 0.816-1.073 | 0.936 | 0.341 | 0.070 | -0.066 | 0.747-1.234 | 0.960 | 0.750 | 0.128 | -0.041 | 0.925-1.108 | 1.012 | 0.793 | 0.046 | 0.012 | 3.103E-03 | 0.659 |
| Waist-to-hip ratio | 333 | 6.219 | 1.119-1.380 | 1.243 | 4.726E-05 | 0.053 | 0.217 | 0.989-1.428 | 1.188 | 0.066 | 0.091 | 0.172 | 0.919-1.611 | 1.217 | 0.172 | 0.143 | 0.196 | 1.136-1.395 | 1.259 | 1.358E-05 | 0.052 | 0.230 | 0.184 | 0.874 |
| Standing height | 707 | 11.221 | 0.828-0.951 | 0.887 | 6.865E-04 | 0.035 | -0.120 | 0.786-0.977 | 0.876 | 0.017 | 0.056 | -0.132 | 0.813-1.075 | 0.935 | 0.345 | 0.071 | -0.068 | 0.831-0.952 | 0.889 | 7.153E-04 | 0.034 | -0.117 | 0.010 | 0.403 |
| Sitting height | 559 | 9.549 | 0.796-0.931 | 0.861 | 1.729E-04 | 0.0400 | -0.150 | 0.756-0.964 | 0.854 | 0.011 | 0.062 | -0.158 | 0.739-1.056 | 0.884 | 0.174 | 0.091 | -0.124 | 0.806-0.938 | 0.870 | 3.317E-04 | 0.039 | -0.140 | 0.040 | 0.748 |
| Hand grip strength (left) | 141 | 10.593 | 0.624-0.972 | 0.779 | 0.027 | 0.113 | -0.250 | 0.529-1.027 | 0.737 | 0.072 | 0.169 | -0.305 | 0.190-1.126 | 0.463 | 0.092 | 0.454 | -0.770 | 0.607-0.968 | 0.766 | 0.027 | 0.119 | -0.266 | 0.146 | 0.236 |
| Hand grip strength (right) | 160 | 2.445 | 0.642-0.972 | 0.790 | 0.026 | 0.106 | -0.236 | 0.546-1.013 | 0.744 | 0.060 | 0.157 | -0.296 | 0.185-0.857 | 0.398 | 0.020 | 0.392 | -0.922 | 0.962-1.338 | 1.135 | 0.151 | 0.084 | 0.126 | 0.548 | 0.105 |
| Birth weight | 165 | 18.232 | 0.733-0.937 | 0.829 | 2.604E-03 | 0.062 | -0.188 | 0.658-0.932 | 0.783 | 5.813E-03 | 0.089 | -0.245 | 0.430-0.850 | 0.604 | 4.322E-03 | 0.174 | -0.504 | 0.736-0.935 | 0.829 | 2.504E-03 | 0.061 | -0.187 | 0.024 | 0.054 |
| Childhood BMI | 18 | 41.695 | 0.945-1.043 | 0.993 | 0.767 | 0.025 | -7.431E-03 | 0.951-1.029 | 0.989 | 0.593 | 0.020 | -0.011 | 0.931-1.037 | 0.982 | 0.528 | 0.028 | -0.018 | 0.945-1.043 | 0.993 | 0.770 | 0.025 | -7.431E-03 | 0.023 | 0.378 |
| **Emotional factors** | | | | | | | | | | | | | | | | | | | | | | | | |
| Major depressive disorder | 44 | 5.287 | 1.297-1.755 | 1.509 | 9.842E-08 | 0.077 | 0.411 | 1.034-1.627 | 1.297 | 0.024 | 0.116 | 0.260 | 0.471-3.005 | 1.190 | 0.715 | 0.473 | 0.174 | 1.310-1.765 | 1.520 | 1.546E-06 | 0.076 | 0.419 | 0.332 | 0.613 |
| Anxious feeling | 22 | 39.568 | 0.888-2.614 | 1.524 | 0.126 | 0.275 | 0.421 | 0.735-2.478 | 1.349 | 0.334 | 0.310 | 0.300 | 0.359-700.032 | 15.851 | 0.168 | 1.933 | 2.763 | 0.888-2.614 | 1.524 | 0.141 | 0.275 | 0.421 | 0.021 | 0.235 |
| Schizophrenia | 131 | 5.674 | 1.006-1.082 | 1.043 | 0.023 | 0.019 | 0.042 | 0.995-1.105 | 1.049 | 0.074 | 0.027 | 0.048 | 0.833-1.149 | 0.978 | 0.790 | 0.082 | -0.022 | 0.997-1.077 | 1.036 | 0.072 | 0.020 | 0.035 | 0.255 | 0.424 |
| Bipolar disorder | 47 | 12.319 | 0.906-1.032 | 0.967 | 0.307 | 0.033 | -0.034 | 0.884-1.063 | 0.969 | 0.507 | 0.047 | -0.031 | 0.704-1.355 | 0.977 | 0.888 | 0.167 | -0.024 | 0.909-1.028 | 0.967 | 0.285 | 0.032 | -0.034 | 0.644 | 0.950 |

| | | | | | | | | | | | | | | | | | | | | | | | | |
|---|---|---|---|---|---|---|---|---|---|---|---|---|---|---|---|---|---|---|---|---|---|---|---|---|
| Anorexia nervosa | 6 | 92.256 | 0.866-1.124 | 0.987 | 0.843 | 0.067 | -0.013 | 0.853-1.200 | 1.012 | 0.894 | 0.087 | 0.012 | 0.463-1.264 | 0.765 | 0.354 | 0.256 | -0.268 | 0.819-1.052 | 0.928 | 0.280 | 0.064 | -0.074 | 0.627 | 0.361 |
| Life satisfaction | 73 | 17.802 | 0.357-0.754 | 0.518 | 5.864E-04 | 0.191 | -0.657 | 0.435-1.427 | 0.788 | 0.432 | 0.303 | -0.238 | 0.008-0.701 | 0.075 | 0.026 | 1.142 | -2.594 | 0.381-0.860 | 0.573 | 8.875E-03 | 0.208 | -0.558 | 0.088 | 0.089 |
| Positive affect | 92 | 14.990 | 0.367-0.724 | 0.516 | 1.334E-04 | 0.173 | -0.662 | 0.319-0.930 | 0.545 | 0.026 | 0.273 | -0.607 | 0.045-1.152 | 0.228 | 0.077 | 0.827 | -1.479 | 0.387-0.801 | 0.557 | 2.151E-03 | 0.186 | -0.586 | 0.106 | 0.313 |
| **Socioeconomic factors** | | | | | | | | | | | | | | | | | | | | | | | | |
| income | 42 | 16.122 | 0.601-0.984 | 0.769 | 0.037 | 0.126 | -0.263 | 0.531-1.086 | 0.760 | 0.132 | 0.182 | -0.275 | 0.279-4.469 | 1.116 | 0.877 | 0.708 | 0.110 | 0.598-1.001 | 0.774 | 0.057 | 0.131 | -0.257 | 0.158 | 0.594 |
| Educational attainment | 1147 | 15.335 | 0.595-0.710 | 0.650 | 1.725E-21 | 0.045 | -0.431 | 0.624-0.794 | 0.704 | 1.056E-08 | 0.061 | -0.351 | 0.551-1.036 | 0.756 | 0.082 | 0.161 | -0.280 | 0.607-0.724 | 0.663 | 1.610E-19 | 0.045 | -0.411 | 1.793E-05 | 0.328 |
| Intelligence | 158 | 15.813 | 0.791-1.025 | 0.900 | 0.113 | 0.066 | -0.105 | 0.762-1.087 | 0.910 | 0.299 | 0.091 | -0.094 | 0.632-2.395 | 1.231 | 0.542 | 0.340 | 0.207 | 0.792-1.024 | 0.900 | 0.112 | 0.066 | -0.105 | 0.048 | 0.350 |

SNP, single nucleotide polymorphism; OR, odds ratio; CI, confidence interval; SE, standard error

[a]*I^2* was utilized to quantify the heterogeneity among SNPs for each exposure.

[b]*P* value for heterogeneity was the *P* value of Cochran's *Q* test. *P* value ≤ 0.05 was deemed as a statistically significant heterogeneity.

[c]*P* value for pleiotropy was the *P* value of MR-Egger intercept test. *P* value ≤ 0.05 was deemed as a statistically significant horizontal pleiotropy.

## Table S6. The summary of the MR results of the meta-analysis

| Exposures | Used SNPs | I^2(%)[a] | IVW-method | | | | | Weighted median method | | | | | MR-Egger method | | | | | MR PRESSO model | | | | | *P* value for heterogeneity[b] | *P* value for pleiotropy[c] |
|---|---|---|---|---|---|---|---|---|---|---|---|---|---|---|---|---|---|---|---|---|---|---|---|---|
| | | | 95%CI | OR | *P* value | SE | BETA | 95%CI | OR | *P* value | SE | BETA | 95%CI | OR | *P* value | SE | BETA | 95%CI | OR | *P* value | SE | BETA | | |
| **Daily habits** | | | | | | | | | | | | | | | | | | | | | | | | |
| Alcohol consumption | 94 | 38.248 | 0.824-1.196 | 0.993 | 0.938 | 0.095 | -7.429E-03 | 0.657-1.128 | 0.861 | 0.277 | 0.138 | -0.150 | 0.596-1284 | 0.874 | 0.495 | 0.196 | -0.134 | 0.818-1.182 | 0.983 | 0.859 | 0.094 | -0.017 | 1.129E-04 | 0.460 |
| Coffee consumption | 29 | 23.895 | 0.723-1.066 | 0.878 | 0.188 | 0.099 | -0.131 | 0.768-1.330 | 1.011 | 0.938 | 0.140 | 0.011 | 0.830-2.039 | 1.301 | 0.262 | 0.229 | 0.263 | 0.691-1.114 | 0.878 | 0.293 | 0.122 | -0.131 | 0.099 | 0.057 |
| Caffeine consumption | 25 | 14.171 | 0.762-1.009 | 0.877 | 0.066 | 0.071 | -0.131 | 0.825-1.224 | 1.005 | 0.959 | 0.101 | 5.113E-03 | 0.930-1.724 | 1.266 | 0.147 | 0.157 | 0.236 | 0.737-1.043 | 0.877 | 0.150 | 0.088 | -0.131 | 0.217 | 0.127 |
| Smoking initiation | 345 | 27.458 | 1.214-1.359 | 1.284 | 2.942E-18 | 0.029 | 0.250 | 1.144-1.334 | 1.236 | 7.766E-08 | 0.040 | 0.212 | 0.880-1.399 | 1.110 | 0.379 | 0.118 | 0.104 | 1.200-1.343 | 1.270 | 1.389E-15 | 0.029 | 0.239 | 3.262E-06 | 0.203 |
| Lifetime smoking | 117 | 27.115 | 1.649-2.356 | 1.971 | 9.127E-14 | 0.091 | 0.678 | 1.409-2.253 | 1.782 | 1.427E-06 | 0.120 | 0.578 | 0.585-2.380 | 1.180 | 0.645 | 0.358 | 0.165 | 1.623-2.292 | 1.929 | 1.443E-11 | 0.088 | 0.657 | 4.051E-03 | 0.141 |
| Cigarette consumption | 51 | 0.473 | 1.105-1.326 | 1.211 | 3.944E-05 | 0.047 | 0.191 | 0.980-1.313 | 1.134 | 0.092 | 0.075 | 0.126 | 0.869-1.209 | 1.025 | 0.771 | 0.084 | 0.025 | 1.110-1.347 | 1.223 | 1.507E-04 | 0.049 | 0.201 | 0.424 | 0.074 |
| Age of initiation of regular smoking | 7 | 389.324 | 0.645-1.330 | 0.926 | 0.678 | 0.185 | -0.077 | 0.527-1.344 | 0.841 | 0.469 | 0.239 | -0.173 | 0.966-46.466 | 6.700 | 0.112 | 0.988 | 1.902 | 0.610-1.430 | 0.934 | 0.621 | 0.351 | -0.180 | 0.942 | 0.097 |
| Smoking cessation | 24 | 17.381 | 1.081-1.344 | 1.206 | 8.558E-04 | 0.056 | 0.187 | 0.979-1.338 | 1.145 | 0.089 | 0.080 | 0.135 | 0.776-1.463 | 1.065 | 0.699 | 0.162 | 0.064 | 1.068-1.362 | 1.206 | 6.170E-03 | 0.062 | 0.187 | 0.181 | 0.415 |
| Intake of total sugar | 8 | 11.364 | 0.641-1.086 | 0.834 | 0.178 | 0.134 | -0.181 | 0.606-1.265 | 0.875 | 0.478 | 0.188 | -0.133 | 0.062-1.811 | 0.335 | 0.251 | 0.861 | -1.094 | 0.614-1.133 | 0.834 | 0.284 | 0.156 | -0.181 | 0.246 | 0.323 |
| Intake of fat | 5 | 26.311 | 0.847-1.553 | 1.147 | 0.375 | 0.155 | 0.137 | 0.790-1.674 | 1.150 | 0.466 | 0.192 | 0.140 | 0.431-2.594 | 1.057 | 0.911 | 0.458 | 0.056 | 0.804-1.636 | 1.147 | 0.492 | 0.181 | 0.137 | 0.143 | 0.855 |
| Intake of carbohydrate | 10 | 21.242 | 0.589-1.033 | 0.780 | 0.083 | 0.143 | -0.249 | 0.568-1.294 | 0.857 | 0.463 | 0.210 | -0.154 | 0.023-1.145 | 0.163 | 0.106 | 0.996 | -1.816 | 0.585-1.158 | 0.823 | 0.290 | 0.174 | -0.194 | 0.179 | 0.149 |
| Breakfast skipping | 6 | 139.588 | 0.833-1.710 | 1.194 | 0.335 | 0.183 | 0.177 | 0.701-1.730 | 1.102 | 0.674 | 0.23 | 0.097 | 0.646-12.170 | 2.804 | 0.241 | 0.749 | 1.031 | 0.885-1.611 | 1.194 | 0.299 | 0.153 | 0.177 | 0.720 | 0.305 |
| Morning Person | 116 | 24.017 | 0.970-1.078 | 1.023 | 0.406 | 0.027 | 0.022 | 0.962-1.115 | 1.036 | 0.351 | 0.038 | 0.035 | 0.889-1.245 | 1.052 | 0.552 | 0.086 | 0.051 | 0.974-1.080 | 1.025 | 0.340 | 0.026 | 0.025 | 0.011 | 0.725 |
| Insomnia | 39 | 41.714 | 1.653-3.344 | 2.351 | 1.957E-06 | 0.180 | 0.855 | 1.504-3.508 | 2.297 | 1.184E-04 | 0.216 | 0.832 | 0.667-6.824 | 2.134 | 0.209 | 0.593 | 0.758 | 1.768-3.532 | 2.499 | 6.522E-06 | 0.177 | 0.916 | 2.858E-03 | 0.865 |
| Short sleep | 23 | 28.845 | 2.019-5.896 | 3.450 | 5.867E-06 | 0.273 | 1.239 | 1.348-7.234 | 3.123 | 7.895E-03 | 0.429 | 1.139 | 2.729-1438.835 | 62.657 | 0.017 | 1.599 | 4.138 | 1.974-7.303 | 3.797 | 5.300E-04 | 0.334 | 1.334 | 0.075 | 0.078 |
| Long sleep | 9 | 43.974 | 0.115-4.781 | 0.742 | 0.754 | 0.950 | -0.298 | 0.041-1.226 | 0.224 | 0.085 | 0.868 | -1.498 | 2.500E-04-35.661 | 0.094 | 0.461 | 3.028 | -2.360 | 0.115-4.781 | 0.742 | 0.762 | 0.950 | -0.298 | 0.046 | 0.495 |
| Daytime sleepiness | 37 | 33.656 | 1.353-3.403 | 2.146 | 1.161E-03 | 0.235 | 0.764 | 1.068-3.327 | 1.885 | 0.029 | 0.290 | 0.634 | 0.296-17.515 | 2.277 | 0.435 | 1.041 | 0.823 | 1.416-3.560 | 2.245 | 1.460E-03 | 0.235 | 0.809 | 0.020 | 0.954 |
| Daytime napping | 102 | 44.414 | 1.087-1.772 | 1.388 | 8.569E-03 | 0.125 | 0.328 | 1.086-1.926 | 1.446 | 0.012 | 0.146 | 0.369 | 0.932-5.198 | 2.202 | 0.075 | 0.438 | 0.789 | 1.072-1.740 | 1.366 | 0.013 | 0.124 | 0.312 | 1.106E-06 | 0.275 |
| Leisure sedentary behavior (TV watching) | 130 | 39.096 | 1.422-1.812 | 1.606 | 1.835E-14 | 0.062 | 0.473 | 1.375-1.857 | 1.598 | 9.679E-10 | 0.077 | 0.469 | 0.751-2.327 | 1.322 | 0.336 | 0.289 | 0.279 | 1.444-1.829 | 1.625 | 3.430E-13 | 0.060 | 0.485 | 4.548E-06 | 0.491 |
| Leisure sedentary behavior (computer use) | 47 | 20.459 | 0.614-0.846 | 0.721 | 6.203E-05 | 0.082 | -0.327 | 0.585-0.940 | 0.742 | 0.014 | 0.121 | -0.298 | 0.083-0.870 | 0.269 | 0.034 | 0.599 | -1.314 | 0.599-0.867 | 0.721 | 1.154E-03 | 0.094 | -0.327 | 0.095 | 0.103 |
| 10+ minutes vigorous activity | 7 | 35.784 | 0.303-1.152 | 0.591 | 0.123 | 0.341 | -0.525 | 0.192-1.236 | 0.488 | 0.130 | 0.475 | -0.718 | 9.040E-06-28.048 | 0.016 | 0.327 | 3.813 | -4.140 | 0.239-1.462 | 0.591 | 0.299 | 0.462 | -0.525 | 0.096 | 0.383 |
| Moderate to vigorous physical activity levels | 18 | 17.077 | 0.668-1.149 | 0.876 | 0.340 | 0.139 | -0.132 | 0.547-1.173 | 0.801 | 0.254 | 0.195 | -0.222 | 0.034-1.251 | 0.206 | 0.105 | 0.920 | -1.578 | 0.635-1.208 | 0.876 | 0.430 | 0.164 | -0.132 | 0.199 | 0.130 |
| Strenuous sports or other exercises | 13 | 12.070 | 0.217-0.734 | 0.399 | 3.138E-03 | 0.311 | -0.919 | 0.249-1.401 | 0.591 | 0.232 | 0.441 | -0.527 | 0.059-34.153 | 1.421 | 0.833 | 1.622 | 0.351 | 0.183-0.692 | 0.356 | 9.438E-03 | 0.340 | -1.033 | 0.456 | 0.945 |
| **Health status** | | | | | | | | | | | | | | | | | | | | | | | | |
| Childhood-onset asthma | 99 | 29.721 | 0.992-1.029 | 1.011 | 0.242 | 9.169E-03 | 0.011 | 0.986-1.037 | 1.011 | 0.379 | 0.013 | 0.011 | 0.959-1.044 | 1.001 | 0.968 | 0.022 | 8.838E-04 | 0.994-1.029 | 1.011 | 0.217 | 8.991E-03 | 0.011 | 3.104E-03 | 0.622 |
| Adult-onset asthma | 45 | 21.027 | 1.012-1.085 | 1.048 | 9.092E-03 | 0.018 | 0.047 | 0.980-1.093 | 1.035 | 0.214 | 0.028 | 0.035 | 0.851-1.100 | 0.967 | 0.615 | 0.065 | -0.033 | 1.006-1.091 | 1.048 | 0.029 | 0.020 | 0.047 | 0.092 | 0.206 |
| Type 2 diabetes | 347 | 23.232 | 1.008-1.045 | 1.026 | 5.447E-03 | 9.212E-03 | 0.026 | 0.976-1.032 | 1.004 | 0.789 | 0.014 | 3.799E-03 | 0.973-1.047 | 1.010 | 0.602 | 0.018 | 9.635E-03 | 1.005-1.040 | 1.022 | 0.014 | 8.902E-03 | 0.022 | 1.084E-04 | 0.318 |
| Coronary artery disease | 144 | 28.995 | 1.006-1.071 | 1.038 | 0.018 | 0.016 | 0.038 | 0.992-1.084 | 1.037 | 0.110 | 0.023 | 0.036 | 0.961-1.091 | 1.024 | 0.470 | 0.033 | 0.024 | 1.006-1.071 | 1.038 | 0.019 | 0.016 | 0.038 | 7.762E-04 | 0.622 |
| Atrial fibrillation | 110 | 8.423 | 0.992-1.037 | 1.014 | 0.200 | 0.011 | 0.014 | 0.986-1.060 | 1.022 | 0.238 | 0.019 | 0.022 | 0.968-1.059 | 1.013 | 0.586 | 0.023 | 0.012 | 0.992-1.038 | 1.015 | 0.203 | 0.017 | 0.015 | 0.220 | 0.923 |
| Ulcerative colitis | 37 | 11.789 | 1.007-1.037 | 1.022 | 4.269E-03 | 7.558E-03 | 0.022 | 1.000-1.047 | 1.023 | 0.048 | 0.012 | 0.023 | 0.980-1.064 | 1.021 | 0.319 | 0.021 | 0.021 | 1.005-1.037 | 1.021 | 0.013 | 7.924E-03 | 0.021 | 0.230 | 0.352 |

| | | | | | | | | | | | | | | | | | | | | | | | | |
|---|---|---|---|---|---|---|---|---|---|---|---|---|---|---|---|---|---|---|---|---|---|---|---|---|
| Crohn's disease | 50 | 29.264 | 0.988-1.017 | 1.002 | 0.771 | 7.315E-03 | 2.128E-03 | 0.974-1.014 | 0.994 | 0.548 | 0.010 | -6.242E-03 | 0.969-1.035 | 1.001 | 0.933 | 0.017 | 1.423E-03 | 0.988-1.016 | 1.002 | 0.819 | 7.0415E-03 | 1.617E-03 | 0.024 | 0.963 |
| **Nutritional & biochemical biomarkers** | | | | | | | | | | | | | | | | | | | | | | | | |
| Fasting glucose | 68 | 1.552 | 0.939-1.138 | 1.033 | 0.501 | 0.049 | 0.033 | 0.954-1.306 | 1.116 | 0.170 | 0.080 | 0.110 | 0.945-1.354 | 1.131 | 0.184 | 0.092 | 0.123 | 0.942-1.140 | 1.036 | 0.469 | 0.049 | 0.036 | 0.407 | 0.246 |
| Fating insulin | 37 | 31.908 | 0.875-1.351 | 1.087 | 0.449 | 0.111 | 0.084 | 0.827-1.407 | 1.079 | 0.577 | 0.136 | 0.076 | 0.391-1.457 | 0.755 | 0.408 | 0.335 | -0.281 | 0.875-1.351 | 1.087 | 0.454 | 0.111 | 0.084 | 0.027 | 0.257 |
| 2-hour blood glucose | 14 | 23.177 | 0.919-1.055 | 0.985 | 0.661 | 0.035 | -0.015 | 0.917-1.108 | 1.008 | 0.873 | 0.048 | 7.680E-03 | 0.747-1.108 | 0.910 | 0.365 | 0.101 | -0.095 | 0.908-1.068 | 0.985 | 0.715 | 0.042 | -0.015 | 0.153 | 0.403 |
| Glycosylated hemoglobin (HbA1c) | 72 | 4.857 | 0.906-1.178 | 1.033 | 0.626 | 0.067 | 0.033 | 0.864-1.323 | 1.069 | 0.539 | 0.109 | 0.067 | 0.853-1.391 | 1.089 | 0.494 | 0.125 | 0.086 | 0.910-1.174 | 1.033 | 0.614 | 0.065 | 0.033 | 0.555 | 0.616 |
| HDL cholesterol | 83 | 17.553 | 0.902-0.981 | 0.941 | 4.171E-03 | 0.021 | -0.061 | 0.887-1.012 | 0.947 | 0.106 | 0.033 | -0.054 | 0.880-1.048 | 0.961 | 0.367 | 0.044 | -0.040 | 0.903-0.988 | 0.944 | 0.014 | 0.023 | -0.057 | 0.080 | 0.577 |
| LDL cholesterol | 79 | 18.138 | 0.927-0.990 | 0.958 | 0.011 | 0.017 | -0.043 | 0.939-1.049 | 0.992 | 0.787 | 0.028 | -7.676E-03 | 0.923-1.027 | 0.973 | 0.322 | 0.027 | -0.027 | 0.927-0.996 | 0.961 | 0.034 | 0.018 | -0.040 | 0.077 | 0.416 |
| Total cholesterol | 86 | 6.709 | 0.944-1.012 | 0.977 | 0.202 | 0.018 | -0.023 | 0.930-1.044 | 0.985 | 0.607 | 0.029 | -0.015 | 0.911-1.025 | 0.966 | 0.258 | 0.030 | -0.034 | 0.948-1.017 | 0.982 | 0.304 | 0.018 | -0.019 | 0.279 | 0.631 |
| Triglycerides | 53 | 42.993 | 0.962-1.090 | 1.024 | 0.451 | 0.032 | 0.024 | 0.944-1.087 | 1.013 | 0.722 | 0.036 | 0.013 | 0.925-1.130 | 1.022 | 0.671 | 0.051 | 0.022 | 0.962-1.090 | 1.024 | 0.454 | 0.032 | 0.024 | 4.618E-04 | 0.957 |
| Adiponectin | 8 | 41.810 | 0.866-1.151 | 0.998 | 0.980 | 0.072 | -1.834E-03 | 0.810-1.189 | 0.982 | 0.851 | 0.098 | -0.018 | 0.771-2.279 | 1.326 | 0.347 | 0.276 | 0.282 | 0.814-1.224 | 0.998 | 0.986 | 0.104 | -1.834E-03 | 0.061 | 0.311 |
| Leptin | 5 | 33.812 | 0.620-0.995 | 0.785 | 0.045 | 0.121 | -0.242 | 0.630-1.349 | 0.922 | 0.674 | 0.194 | -0.082 | 1.971E-04-5.122 | 0.032 | 0.276 | 2.593 | -3.449 | 0.575-1.050 | 0.777 | 0.162 | 0.154 | -0.253 | 0.110 | 0.303 |
| Vitamin C | 10 | 42.561 | 0.874-1.098 | 0.980 | 0.725 | 0.058 | -0.020 | 0.841-1.035 | 0.933 | 0.188 | 0.053 | -0.070 | 0.761-1.093 | 0.912 | 0.350 | 0.092 | -0.092 | 0.886-1.106 | 0.990 | 0.861 | 0.057 | -0.010 | 0.047 | 0.348 |
| 25-Hydroxyvitamin D | 115 | 16.839 | 0.952-1.084 | 1.016 | 0.630 | 0.033 | 0.016 | 0.890-1.101 | 0.990 | 0.847 | 0.054 | -0.010 | 0.839-1.058 | 0.942 | 0.317 | 0.059 | -0.060 | 0.948-1.097 | 1.020 | 0.598 | 0.037 | 0.020 | 0.061 | 0.108 |
| **Nutritional & developmental status** | | | | | | | | | | | | | | | | | | | | | | | | |
| Body mass index | 437 | 26.108 | 1.317-1.468 | 1.391 | 1.859E-32 | 0.028 | 0.330 | 1.193-1.391 | 1.288 | 1.083E-10 | 0.039 | 0.253 | 0.942-1.269 | 1.093 | 0.242 | 0.076 | 0.089 | 1.313-1.462 | 1.386 | 8.431E-29 | 0.027 | 0.326 | 9.744E-07 | 0.096 |
| Body fat percentage | 378 | 34.633 | 1.377-1.621 | 1.494 | 4.751E-22 | 0.042 | 0.402 | 1.210-1.515 | 1.354 | 1.209E-07 | 0.057 | 0.303 | 1.002-1.699 | 1.304 | 0.049 | 0.135 | 0.266 | 1.372-1.612 | 1.487 | 5.424E-20 | 0.041 | 0.397 | 1.157E-10 | 0.290 |
| Whole body fat mass | 415 | 27.917 | 1.225-1.373 | 1.297 | 3.035E-19 | 0.029 | 0.260 | 1.103-1.321 | 1.207 | 4.319E-05 | 0.046 | 0.188 | 0.999-1.382 | 1.176 | 0.051 | 0.083 | 0.162 | 1.228-1.374 | 1.299 | 2.373E-18 | 0.029 | 0.262 | 2.366E-07 | 0.204 |
| Whole body fat-free mass | 531 | 14.325 | 0.857-0.980 | 0.916 | 0.011 | 0.034 | -0.087 | 0.812-0.986 | 0.895 | 0.025 | 0.049 | -0.111 | 0.698-0.965 | 0.821 | 0.017 | 0.083 | -0.197 | 0.863-0.986 | 0.923 | 0.018 | 0.034 | -0.081 | 4.404E-08 | 0.143 |
| Visceral adipose tissue | 180 | 34.109 | 1.222-1.413 | 1.314 | 2.002E-13 | 0.037 | 0.273 | 1.037-1.272 | 1.148 | 8.006E-03 | 0.052 | 0.138 | 0.892-1.408 | 1.121 | 0.330 | 0.117 | 0.114 | 1.211-1.391 | 1.298 | 4.662E-12 | 0.035 | 0.261 | 7.647E-06 | 0.152 |
| Waist circumference | 358 | 41.395 | 1.298-1.514 | 1.402 | 9.657E-18 | 0.039 | 0.338 | 1.145-1.413 | 1.272 | 7.070E-06 | 0.054 | 0.241 | 0.966-1.520 | 1.212 | 0.097 | 0.115 | 0.192 | 1.297-1.511 | 1.400 | 1.745E-16 | 0.039 | 0.336 | 1.467E-15 | 0.180 |
| Hip circumference | 399 | 31.554 | 1.087-1.219 | 1.151 | 1.614E-06 | 0.029 | 0.141 | 1.022-1.211 | 1.113 | 0.014 | 0.043 | 0.107 | 0.904-1.244 | 1.060 | 0.473 | 0.081 | 0.059 | 1.087-1.218 | 1.151 | 1.674E-06 | 0.029 | 0.140 | 4.092E-09 | 0.282 |
| Waist-to-hip ratio | 337 | 28.011 | 1.280-1.469 | 1.371 | 1.978E-19 | 0.035 | 0.316 | 1.212-1.466 | 1.333 | 3.209E-09 | 0.049 | 0.288 | 0.961-1.394 | 1.157 | 0.123 | 0.095 | 0.146 | 1.276-1.460 | 1.365 | 1.389E-17 | 0.035 | 0.311 | 2.461E-06 | 0.055 |
| Standing height | 740 | 24.560 | 0.864-0.938 | 0.900 | 7.002E-07 | 0.021 | -0.105 | 0.842-0.952 | 0.895 | 4.056E-04 | 0.031 | -0.111 | 0.889-1.050 | 0.966 | 0.415 | 0.043 | -0.035 | 0.860-0.933 | 0.896 | 1.504E-07 | 0.021 | -0.110 | 5.136E-09 | 0.057 |
| Sitting height | 573 | 20.804 | 0.835-0.917 | 0.875 | 1.739E-08 | 0.024 | -0.134 | 0.834-0.962 | 0.896 | 2.514E-03 | 0.036 | -0.110 | 0.799-0.986 | 0.887 | 0.026 | 0.054 | -0.120 | 0.839-0.919 | 0.878 | 3.119E-08 | 0.023 | -0.130 | 0.040 | 0.748 |
| Hand grip strength (left) | 154 | 18.542 | 0.646-0.845 | 0.739 | 9.596E-06 | 0.068 | -0.303 | 0.683-0.983 | 0.819 | 0.032 | 0.093 | -0.199 | 0.316-0.853 | 0.519 | 0.011 | 0.254 | -0.655 | 0.641-0.838 | 0.733 | 1.070E-05 | 0.068 | -0.310 | 0.026 | 0.151 |
| Hand grip strength (right) | 166 | 20.187 | 0.677-0.877 | 0.771 | 7.802E-05 | 0.066 | -0.260 | 0.671-0.972 | 0.808 | 0.024 | 0.094 | -0.213 | 0.331-0.832 | 0.525 | 6.724E-03 | 0.235 | -0.645 | 0.680-0.876 | 0.772 | 8.903E-05 | 0.064 | -0.259 | 0.013 | 0.090 |
| Birth weight | 170 | 27.181 | 0.802-0.925 | 0.861 | 4.522E-05 | 0.037 | -0.149 | 0.784-0.953 | 0.864 | 3.521E-03 | 0.050 | -0.146 | 0.636-0.941 | 0.773 | 0.011 | 0.100 | -0.257 | 0.809-0.932 | 0.868 | 1.262E-04 | 0.036 | -0.141 | 7.747E-04 | 0.251 |
| Childhood BMI | 18 | 58.208 | 0.970-1.041 | 1.005 | 0.796 | 0.018 | 4.655E-03 | 0.974-1.023 | 0.998 | 0.871 | 0.012 | -2.025E-03 | 0.957-1.034 | 0.995 | 0.790 | 0.020 | -5.334E-03 | 0.970-1.041 | 1.005 | 0.799 | 0.018 | 4.655E-03 | 6.195E-04 | 0.257 |
| **Emotional factors** | | | | | | | | | | | | | | | | | | | | | | | | |
| Major depressive disorder | 47 | 2.857 | 1.417-1.671 | 1.539 | 1.084E-24 | 0.042 | 0.431 | 1.344-1.719 | 1.520 | 2.578E-11 | 0.063 | 0.419 | 1.399-3.503 | 2.214 | 1.448E-03 | 0.234 | 0.795 | 1.410-1.669 | 1.534 | 2.292E-13 | 0.043 | 0.428 | 0.377 | 0.121 |
| Anxious feeling | 21 | 36.284 | 1.414-2.624 | 1.926 | 3.214E-05 | 0.158 | 0.656 | 1.491-3.025 | 2.124 | 3.000E-05 | 0.180 | 0.753 | 1.047-88.907 | 9.649 | 0.060 | 1.133 | 2.267 | 1.414-2.624 | 1.926 | 4.865E-04 | 0.158 | 0.656 | 0.037 | 0.167 |
| Schizophrenia | 195 | 30.834 | 0.992-1.042 | 1.016 | 0.200 | 0.013 | 0.016 | 0.986-1.051 | 1.018 | 0.264 | 0.016 | 0.018 | 0.916-1.147 | 1.025 | 0.666 | 0.057 | 0.025 | 0.988-1.037 | 1.012 | 0.325 | 0.012 | 0.012 | 3.925E-05 | 0.877 |
| Bipolar disorder | 52 | 38.391 | 0.930-1.021 | 0.975 | 0.278 | 0.024 | -0.026 | 0.914-1.025 | 0.968 | 0.263 | 0.029 | -0.033 | 0.799-1.348 | 1.038 | 0.782 | 0.133 | 0.037 | 0.930-1.019 | 0.973 | 0.251 | 0.023 | -0.027 | 2.436E-03 | 0.634 |
| Anorexia Nervosa | 6 | 154.389 | 0.879-1.019 | 0.947 | 0.144 | 0.038 | -0.055 | 0.846-1.013 | 0.926 | 0.094 | 0.046 | -0.077 | 0.645-1.138 | 0.857 | 0.346 | 0.145 | -0.154 | 0.915-1.005 | 0.959 | 0.126 | 0.024 | -0.042 | 0.742 | 0.516 |
| Life satisfaction | 76 | 34.808 | 0.318-0.533 | 0.412 | 1.464E-11 | 0.131 | -0.887 | 0.314-0.593 | 0.432 | 2.226E-07 | 0.162 | -0.840 | 0.071-1.073 | 0.275 | 0.067 | 0.694 | -1.289 | 0.332-0.555 | 0.429 | 8.493E-09 | 0.131 | -0.846 | 1.591E-03 | 0.557 |
| Positive affect | 91 | 29.906 | 0.337-0.538 | 0.426 | 6.869E-13 | 0.119 | -0.853 | 0.316-0.589 | 0.431 | 1.240E-07 | 0.159 | -0.841 | 0.231-1.718 | 0.630 | 0.369 | 0.512 | -0.462 | 0.360-0.571 | 0.453 | 1.548E-09 | 0.118 | -0.791 | 3.999E-03 | 0.434 |
| **Socioeconomic factors** | | | | | | | | | | | | | | | | | | | | | | | | |

| | | | | | | | | | | | | | | | | | | | | | | | | |
|---|---|---|---|---|---|---|---|---|---|---|---|---|---|---|---|---|---|---|---|---|---|---|---|---|
| Income | 43 | 19.253 | 0.540-0.709 | 0.619 | 5.427E-12 | 0.070 | -0.480 | 0.486-0.739 | 0.600 | 1.677E-06 | 0.107 | -0.511 | 0.386-1.503 | 0.762 | 0.437 | 0.3466 | -0.272 | 0.543-0.727 | 0.629 | 1.291E-07 | 0.074 | -0.464 | 0.116 | 0.542 |
| Educational attainment | 1206 | 18.617 | 0.514-0.568 | 0.540 | 1.121E-127 | 0.026 | -0.616 | 0.510-0.588 | 0.548 | 1.375E-61 | 0.036 | -0.602 | 0.480-0.683 | 0.573 | 6.886E-10 | 0.090 | -0.558 | 0.522-0.576 | 0.548 | 6.766E-104 | 0.025 | -0.603 | 6.844E-08 | 0.500 |
| Intelligence | 164 | 30.937 | 0.697-0.816 | 0.754 | 2.252E-12 | 0.040 | -0.283 | 0.694-0.848 | 0.767 | 2.397E-07 | 0.051 | -0.265 | 0.495-1.060 | 0.724 | 0.099 | 0.194 | -0.323 | 0.697-0.816 | 0.754 | 5.172E-11 | 0.040 | -0.283 | 1.316E-04 | 0.834 |

SNP, single nucleotide polymorphism; OR, odds ratio; CI, confidence interval; SE, standard error

[a] *I^2* was utilized to quantify the heterogeneity among SNPs for each exposure.

[b] *P* value for heterogeneity was the *P* value of Cochran's *Q* test. *P* value ≤ 0.05 was deemed as a statistically significant heterogeneity.

[c] *P* value for pleiotropy was the *P* value of MR-Egger intercept test. *P* value ≤ 0.05 was deemed as a statistically significant horizontal pleiotropy.

## Table S7. The summary of the MVMR results with MVMR-IVW and MVMR-Egger method

| | Exposures | N.SNPs | MVMR-IVW | | | | | MVMR-Egger | | | | | *P* value for pleiotropy[a] |
|---|---|---|---|---|---|---|---|---|---|---|---|---|---|
| | | | 95%CI | OR | *P* value | SE | BETA | 95%CI | OR | *P* value | SE | BETA | |
| **Hub exposures** | Educational attainment | 156 | 0.516-0.650 | 0.579 | 1.426E-20 | 0.059 | -0.546 | 0.516-0.651 | 0.579 | 1.235E-18 | 0.059 | -0.546 | 0.967 |
| | Smoking initiation | 25 | 1.007-1.231 | 1.114 | 0.036 | 0.051 | 0.108 | 1.007-1.232 | 1.114 | 0.037 | 0.051 | 0.108 | |
| | BMI | 328 | 1.136-1.296 | 1.214 | 8.715E-09 | 0.034 | 0.194 | 1.136-1.297 | 1.214 | 1.641E-08 | 0.034 | 0.194 | |
| **BMI, smoking initiation and educational attainment adjusted** | Cigarette consumption | 9 | 0.927-1.206 | 1.057 | 0.407 | 0.067 | 0.056 | 0.926-1.206 | 1.057 | 0.413 | 0.067 | 0.055 | 0.816 |
| | BMI | 323 | 1.127-1.294 | 1.207 | 9.099E-08 | 0.035 | 0.189 | 1.127-1.293 | 1.207 | 1.471E-07 | 0.035 | 0.188 | |
| | Smoking initiation | 24 | 1.001-1.227 | 1.108 | 0.048 | 0.052 | 0.103 | 1.001-1.227 | 1.108 | 0.049 | 0.052 | 0.103 | |
| | Educational attainment | 157 | 0.520-0.656 | 0.584 | 9.685E-20 | 0.059 | -0.538 | 0.519-0.656 | 0.584 | 5.121E-18 | 0.060 | -0.539 | |
| **BMI, smoking initiation and educational attainment adjusted** | Insomnia | 6 | 1.684-3.104 | 2.286 | 1.176E-07 | 0.156 | 0.827 | 1.291-2.363 | 1.747 | 3.283E-04 | 0.154 | 0.558 | 0.765 |
| | BMI | 326 | 1.122-1.276 | 1.196 | 5.026E-08 | 0.033 | 0.179 | 1.083-1.283 | 1.179 | 1.445E-03 | 0.043 | 0.165 | |
| | Smoking initiation | 25 | 0.983-1.194 | 1.083 | 0.108 | 0.050 | 0.080 | 0.876-1.093 | 0.979 | 0.701 | 0.056 | -0.021 | |
| | Educational attainment | 155 | 0.552-0.695 | 0.620 | 3.363E-16 | 0.059 | -0.479 | 0.494-0.662 | 0.572 | 2.003E-13 | 0.075 | -0.559 | |
| **BMI, smoking initiation and educational attainment adjusted** | Short sleep | 7 | 2.281-3.409 | 2.788 | 2.134E-05 | 0.103 | 1.025 | 2.207-3.541 | 2.795 | 9.198E-05 | 0.121 | 1.028 | 0.859 |
| | BMI | 328 | 1.110-1.266 | 1.185 | 4.161E-07 | 0.034 | 0.170 | 1.110-1.266 | 1.185 | 6.085E-07 | 0.034 | 0.170 | |
| | Smoking initiation | 25 | 0.989-1.207 | 1.092 | 0.082 | 0.051 | 0.088 | 0.989-1.207 | 1.093 | 0.082 | 0.051 | 0.089 | |
| | Educational attainment | 156 | 0.547-0.693 | 0.615 | 8.425E-16 | 0.060 | -0.485 | 0.547-0.695 | 0.616 | 1.611E-14 | 0.061 | -0.484 | |
| **BMI, smoking initiation and educational attainment adjusted** | Leisure sedentary behavior (TV watching) | 46 | 0.985-1.490 | 1.212 | 0.069 | 0.106 | 0.192 | 0.987-1.498 | 1.216 | 0.067 | 0.106 | 0.196 | 0.762 |
| | BMI | 260 | 1.096-1.277 | 1.183 | 1.593E-05 | 0.039 | 0.168 | 1.096-1.277 | 1.183 | 2.081E-05 | 0.039 | 0.168 | |
| | Smoking initiation | 23 | 0.972-1.198 | 1.079 | 0.155 | 0.053 | 0.076 | 0.972-1.198 | 1.079 | 0.157 | 0.053 | 0.076 | |
| | Educational attainment | 133 | 0.541-0.766 | 0.644 | 6.683E-07 | 0.089 | -0.441 | 0.542-0.771 | 0.646 | 1.799E-06 | 0.090 | -0.436 | |
| **BMI, smoking initiation and educational attainment adjusted** | Waist-to-hip ratio | 188 | 1.089-1.313 | 1.195 | 1.837E-04 | 0.048 | 0.179 | 1.089-1.314 | 1.196 | 2.075E-04 | 0.048 | 0.179 | 0.909 |
| | BMI | 263 | 1.032-1.214 | 1.119 | 6.741E-03 | 0.042 | 0.113 | 1.031-1.214 | 1.119 | 7.054E-03 | 0.042 | 0.113 | |
| | Smoking initiation | 23 | 0.951-1.172 | 1.056 | 0.305 | 0.053 | 0.055 | 0.951-1.172 | 1.056 | 0.312 | 0.053 | 0.054 | |
| | Educational attainment | 139 | 0.513-0.652 | 0.578 | 4.685E-19 | 0.062 | -0.549 | 0.511-0.652 | 0.577 | 1.625E-17 | 0.062 | -0.550 | |
| **BMI, smoking initiation and educational attainment adjusted** | Standing height | 658 | 0.903-0.988 | 0.944 | 0.013 | 0.023 | -0.057 | 0.902-0.987 | 0.944 | 0.013 | 0.023 | -0.058 | 0.803 |
| | BMI | 93 | 1.067-1.267 | 1.162 | 6.034E-04 | 0.044 | 0.150 | 1.067-1.268 | 1.163 | 6.207E-04 | 0.044 | 0.151 | |
| | Smoking initiation | 11 | 0.899-1.124 | 1.005 | 0.924 | 0.057 | 5.448E-03 | 0.899-1.124 | 1.006 | 0.923 | 0.057 | 5.492E-03 | |
| | Educational attainment | 47 | 0.478-0.643 | 0.555 | 7.532E-15 | 0.076 | -0.590 | 0.478-0.645 | 0.555 | 3.376E-14 | 0.076 | -0.588 | |
| **BMI, smoking initiation and educational attainment adjusted** | Sitting height | 448 | 0.880-0.978 | 0.928 | 5.544E-03 | 0.027 | -0.075 | 0.880-0.978 | 0.928 | 5.735E-03 | 0.027 | -0.075 | 0.707 |
| | BMI | 149 | 1.028-1.205 | 1.113 | 8.281E-03 | 0.041 | 0.107 | 1.027-1.204 | 1.112 | 9.268E-03 | 0.041 | 0.106 | |
| | Smoking initiation | 14 | 0.903-1.134 | 1.012 | 0.838 | 0.058 | 0.012 | 0.902-1.133 | 1.011 | 0.855 | 0.058 | 0.011 | |
| | Educational attainment | 81 | 0.526-0.698 | 0.606 | 4.532E-12 | 0.073 | -0.501 | 0.525-0.697 | 0.605 | 1.071E-11 | 0.073 | -0.503 | |
| **BMI, smoking initiation and educational attainment adjusted** | Major depressive disorder | 18 | 1.240-1.493 | 1.361 | 7.761E-11 | 0.047 | 0.308 | 1.241-1.495 | 1.362 | 1.919E-10 | 0.047 | 0.308 | 0.670 |
| | BMI | 321 | 1.122- 1.274 | 1.195 | 3.458E-08 | 0.032 | 0.178 | 1.122-1.274 | 1.196 | 5.619E-08 | 0.032 | 0.179 | |
| | Smoking initiation | 22 | 0.946-1.154 | 1.045 | 0.389 | 0.051 | 0.044 | 0.946-1.154 | 1.045 | 0.382 | 0.051 | 0.044 | |
| | Educational attainment | 151 | 0.543-0.680 | 0.608 | 2.930E-18 | 0.057 | -0.498 | 0.544-0.683 | 0.610 | 1.571E-16 | 0.058 | -0.495 | |
| **BMI, smoking initiation and educational attainment adjusted** | Anxious feeling | 3 | 1.194-1.847 | 1.485 | 3.753E-04 | 0.111 | 0.396 | 1.196-1.851 | 1.488 | 3.993E-04 | 0.111 | 0.397 | 0.489 |
| | BMI | 320 | 1.184-1.362 | 1.270 | 2.055E-11 | 0.036 | 0.239 | 1.185-1.363 | 1.271 | 5.861E-11 | 0.036 | 0.240 | |
| | Smoking initiation | 24 | 0.995-1.215 | 1.099 | 0.064 | 0.051 | 0.095 | 0.995-1.216 | 1.100 | 0.063 | 0.051 | 0.096 | |
| | Educational attainment | 152 | 0.535-0.678 | 0.602 | 4.206E-17 | 0.060 | -0.508 | 0.536-0.681 | 0.604 | 1.641E-15 | 0.061 | -0.504 | |
| **BMI, smoking initiation and educational attainment** | Life satisfaction | 14 | 0.342-0.624 | 0.462 | 4.673E-07 | 0.153 | -0.772 | 0.341-0.623 | 0.461 | 7.215E-07 | 0.154 | -0.775 | 0.799 |
| | BMI | 270 | 1.148-1.326 | 1.234 | 1.131E-08 | 0.037 | 0.210 | 1.148-1.327 | 1.234 | 2.251E-08 | 0.037 | 0.211 | |

| | | | | | | | | | | | | | |
|---|---|---|---|---|---|---|---|---|---|---|---|---|---|
| **adjusted** | Smoking initiation | 22 | 0.912-1.139 | 1.019 | 0.737 | 0.057 | 0.019 | 0.912-1.140 | 1.020 | 0.732 | 0.057 | 0.020 | |
| | Educational attainment | 130 | 0.542-0.692 | 0.612 | 3.158E-15 | 0.062 | -0.491 | 0.542-0.695 | 0.614 | 1.156E-13 | 0.063 | -0.488 | |
| **BMI, smoking initiation and educational attainment adjusted** | Positive affect | 20 | 0.385-0.699 | 0.519 | 1.597E-05 | 0.152 | -0.656 | 0.384-0.698 | 0.518 | 2.017E-05 | 0.152 | -0.658 | 0.835 |
| | BMI | 270 | 1.147-1.328 | 1.234 | 1.574E-08 | 0.037 | 0.210 | 1.148-1.329 | 1.235 | 3.082E-08 | 0.037 | 0.211 | |
| | Smoking initiation | 23 | 0.915-1.143 | 1.023 | 0.696 | 0.057 | 0.022 | 0.915-1.144 | 1.023 | 0.689 | 0.057 | 0.023 | |
| | Educational attainment | 126 | 0.529-0.677 | 0.598 | 2.754E-16 | 0.063 | -0.514 | 0.529-0.680 | 0.600 | 1.409E-14 | 0.064 | -0.511 | |
| **BMI and educational attainment adjusted** | Leisure sedentary behavior (computer use) | 11 | 0.782-1.209 | 0.972 | 0.799 | 0.111 | -0.028 | 0.781-1.209 | 0.972 | 0.799 | 0.111 | -0.028 | 0.994 |
| | BMI | 344 | 1.163-1.322 | 1.240 | 5.245E-11 | 0.033 | 0.215 | 1.163-1.322 | 1.398 | 2.360E-21 | 0.033 | 0.335 | |
| | Educational attainment | 155 | 0.426-0.579 | 0.497 | 5.931E-19 | 0.079 | -0.700 | 0.483-0.635 | 0.554 | 2.616E-16 | 0.070 | -0.591 | |
| **BMI and educational attainment adjusted** | Hand grip strength (left) | 48 | 0.715-0.983 | 0.838 | 0.031 | 0.081 | -0.177 | 0.715-0.984 | 0.839 | 0.035 | 0.081 | -0.176 | 0.753 |
| | BMI | 320 | 1.169-1.324 | 1.244 | 5.723E-12 | 0.031 | 0.219 | 1.168-1.324 | 1.244 | 2.282E-11 | 0.032 | 0.218 | |
| | Educational attainment | 148 | 0.514-0.639 | 0.573 | 7.902E-24 | 0.055 | -0.557 | 0.513-0.638 | 0.572 | 1.265E-21 | 0.056 | -0.558 | |
| **BMI and educational attainment adjusted** | Hand grip strength (right) | 49 | 0.689-0.977 | 0.820 | 0.027 | 0.089 | -0.197 | 0.689-0.977 | 0.820 | 0.027 | 0.089 | -0.198 | 0.637 |
| | BMI | 317 | 1.168-1.324 | 1.244 | 7.769E-12 | 0.032 | 0.218 | 1.167-1.323 | 1.243 | 2.973E-11 | 0.032 | 0.217 | |
| | Educational attainment | 146 | 0.515-0.640 | 0.574 | 1.413E-23 | 0.055 | -0.555 | 0.514-0.639 | 0.573 | 1.654E-21 | 0.056 | -0.557 | |
| **BMI and educational attainment adjusted** | Birth weight | 15 | 0.696-0.941 | 0.811 | 6.940E-03 | 0.078 | -0.210 | 0.696-0.944 | 0.811 | 7.215E-03 | 0.078 | -0.210 | 0.781 |
| | BMI | 326 | 1.165-1.328 | 1.244 | 5.636E-11 | 0.033 | 0.218 | 1.165-1.329 | 1.244 | 1.573E-10 | 0.033 | 0.219 | |
| | Educational attainment | 119 | 0.522-0.667 | 0.590 | 2.984E-17 | 0.063 | -0.528 | 0.522-0.668 | 0.590 | 6.726E-16 | 0.063 | -0.527 | |

N.SNPs, numbers of single nucleotide polymorphisms; OR, odds ratio; CI, confidence interval; SE, standard error

[a] *P* value for pleiotropy was the *p* value of MVMR-Egger intercept test. *P* value < 0.05 was deemed as a statistically significant horizontal pleiotropy.

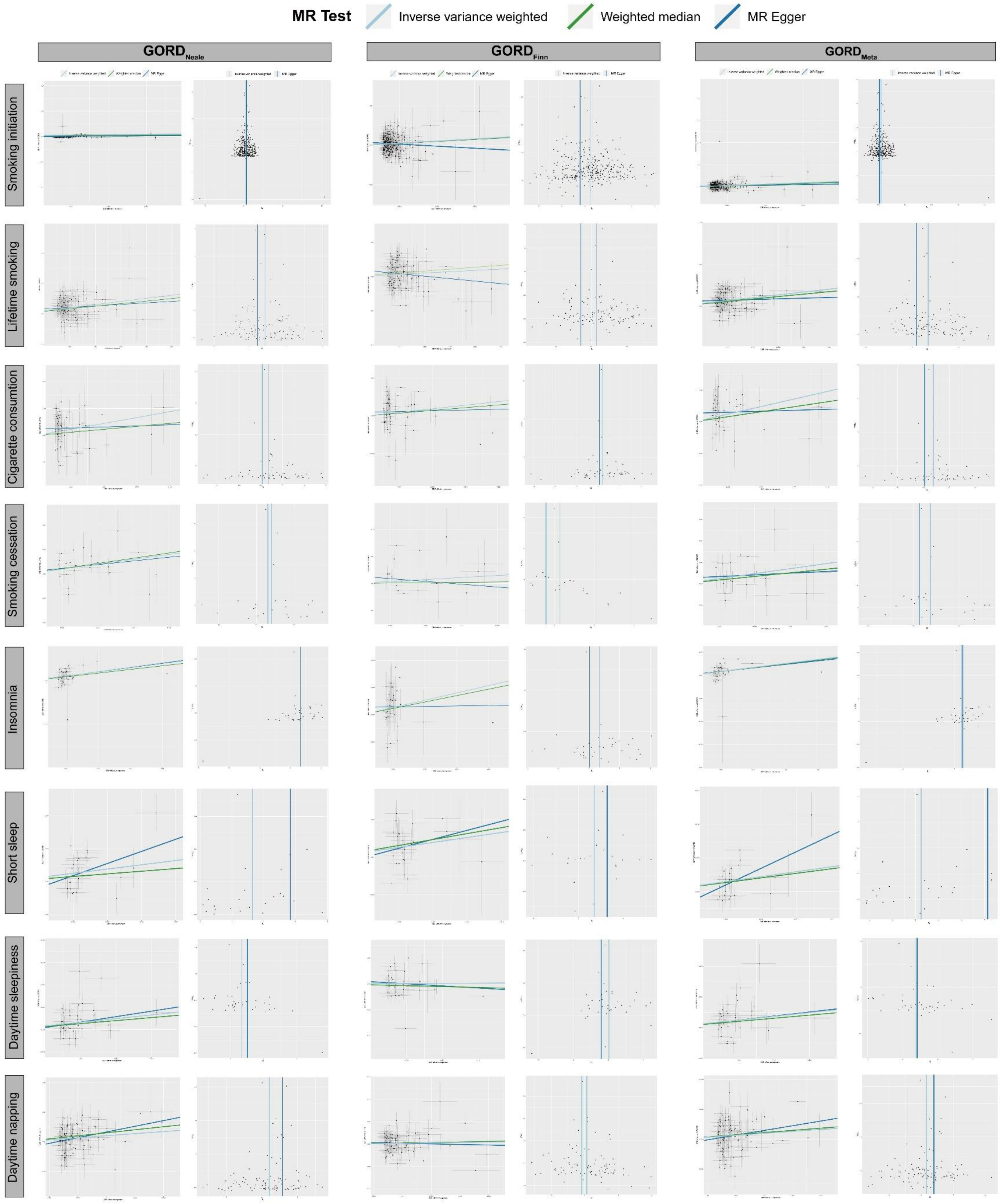
MR Test
Inverse variance weighted
Weighted median
MR Egger
GORD$_{Neale}$
GORD$_{Finn}$
GORD$_{Meta}$
Smoking initiation
Lifetime smoking
Cigarette consumtion
Smoking cessation
Insomnia
Short sleep
Daytime sleepiness
Daytime napping

**Figure S1. Scatter plots and funnel plots for exposures (smoking initiation, lifetime smoking, cigarette consumption, smoking cessation, insomnia, short sleep, daytime sleepiness and daytime napping) for $GORD_{Neale}$, $GORD_{Finn}$ and $GORD_{Meta}$**

In scatter plot, X and Y axes respectively represented the genetic correlation of IVs (dots) on the exposures and outcomes, whose ratio was the slope of the slashes. The upward slashes represented the positive causal effects of exposures on the outcomes. On the contrary, the downward slashes represented the negative causal effects of exposures on the outcomes. In funnel plots, whether the distributions of IVs (dots) is symmetrical or not was used to judge the possible heterogeneity visually. MR-Egger regression was the main method to quantify the heterogeneity.

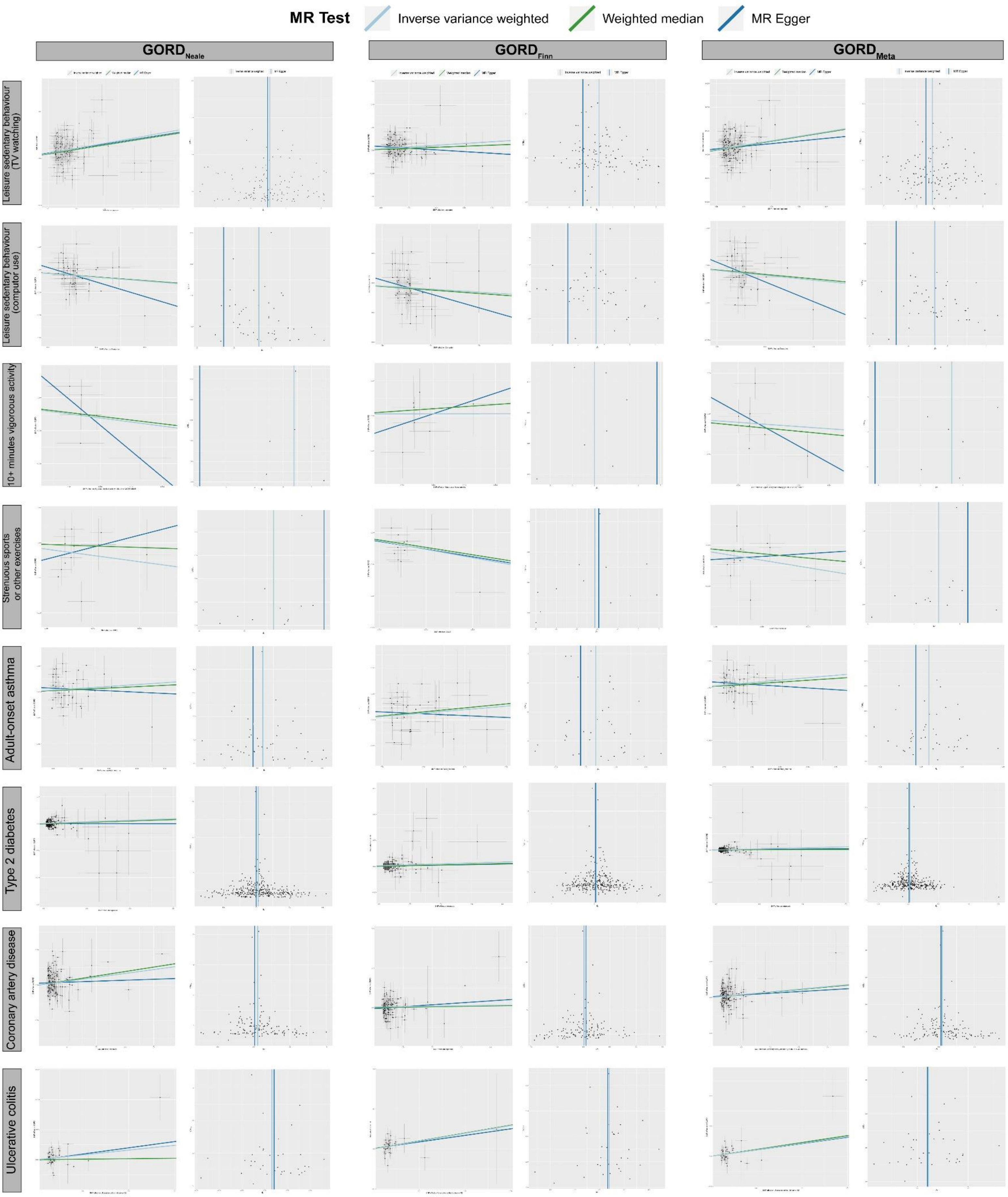
MR Test
Inverse variance weighted
Weighted median
MR Egger
GORD$_{Neale}$
GORD$_{Finn}$
GORD$_{Meta}$
Leisure sedentary behaviour (TV watching)
Leisure sedentary behaviour (computor use)
10+ minutes vigorous activity
Strenuous sports or other exercises
Adult-onset asthma
Type 2 diabetes
Coronary artery disease
Ulcerative colitis

**Figure S2. Scatter plots and funnel plots for exposures (leisure sedentary behavior (TV watching), Leisure sedentary behavior (computer use), 10+ minutes vigorous activity, strenuous sports or other exercises, adult-onset asthma, type 2 diabetes, coronary artery disease and ulcerative colitis) for $GORD_{Neale}$, $GORD_{Finn}$ and $GORD_{Meta}$**

In scatter plot, X and Y axes respectively represented the genetic correlation of IVs (dots) on the exposures and outcomes, whose ratio was the slope of the slashes. The upward slashes represented the positive causal effects of exposures on the outcomes. On the contrary, the downward slashes represented the negative causal effects of exposures on the outcomes. In funnel plots, whether the distributions of IVs (dots) is symmetrical or not was used to judge the possible heterogeneity visually. MR-Egger regression was the main method to quantify the heterogeneity.

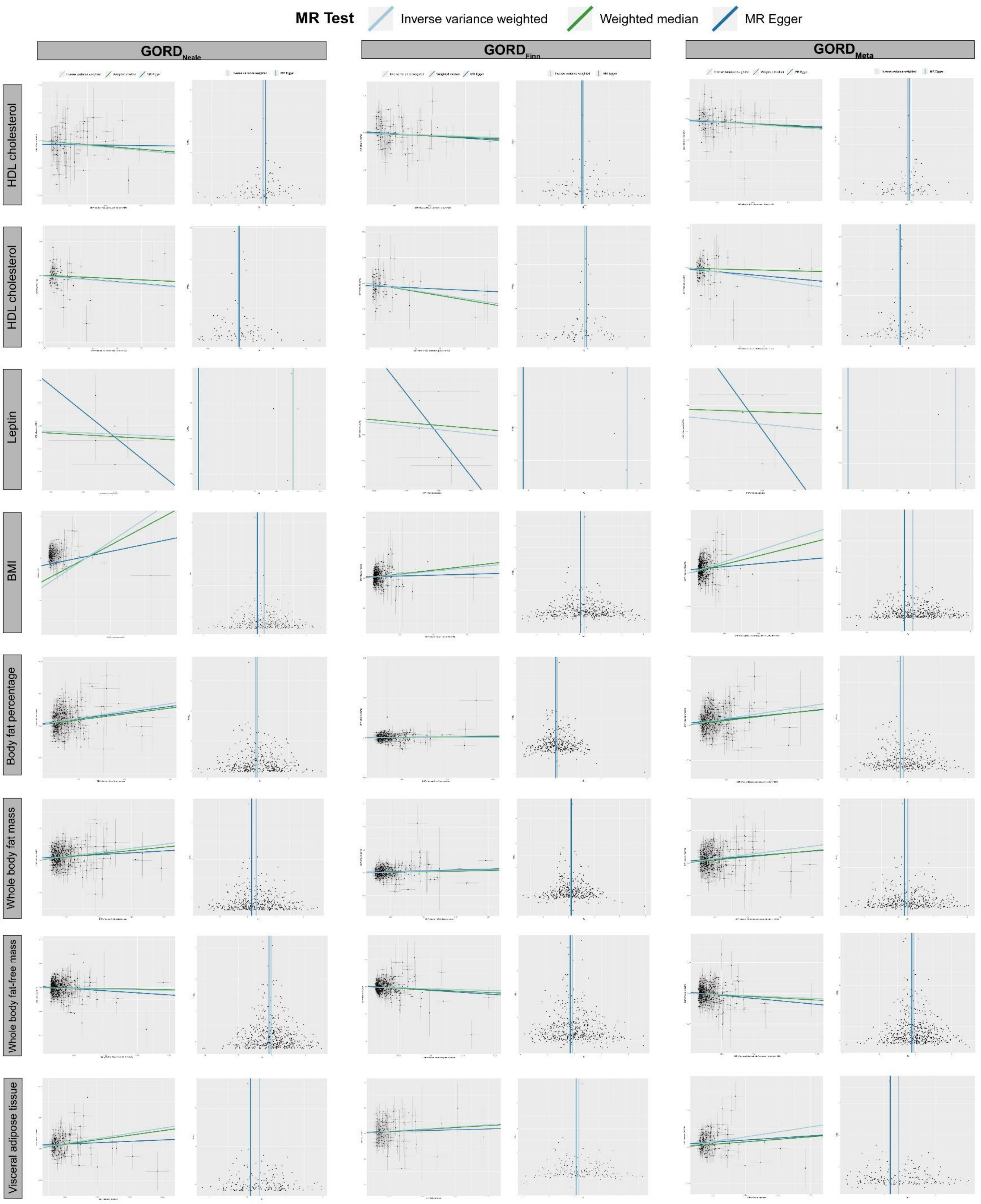

MR Test
Inverse variance weighted
Weighted median
MR Egger
GORD$_{Neale}$
GORD$_{Finn}$
GORD$_{Meta}$
HDL cholesterol
HDL cholesterol
Leptin
BMI
Body fat percentage
Whole body fat mass
Whole body fat-free mass
Visceral adipose tissue

**Figure S3. Scatter plots and funnel plots for exposures (HDL cholesterol, LDL cholesterol, leptin, BMI, body fat percentage, whole body fat mass, whole body fat-free mass and visceral adipose tissue) for $GORD_{Neale}$, $GORD_{Finn}$ and $GORD_{Meta}$**

In scatter plot, X and Y axes respectively represented the genetic correlation of IVs (dots) on the exposures and outcomes, whose ratio was the slope of the slashes. The upward slashes represented the positive causal effects of exposures on the outcomes. On the contrary, the downward slashes represented the negative causal effects of exposures on the outcomes. In funnel plots, whether the distributions of IVs (dots) is symmetrical or not was used to judge the possible heterogeneity visually. MR-Egger regression was the main method to quantify the heterogeneity.

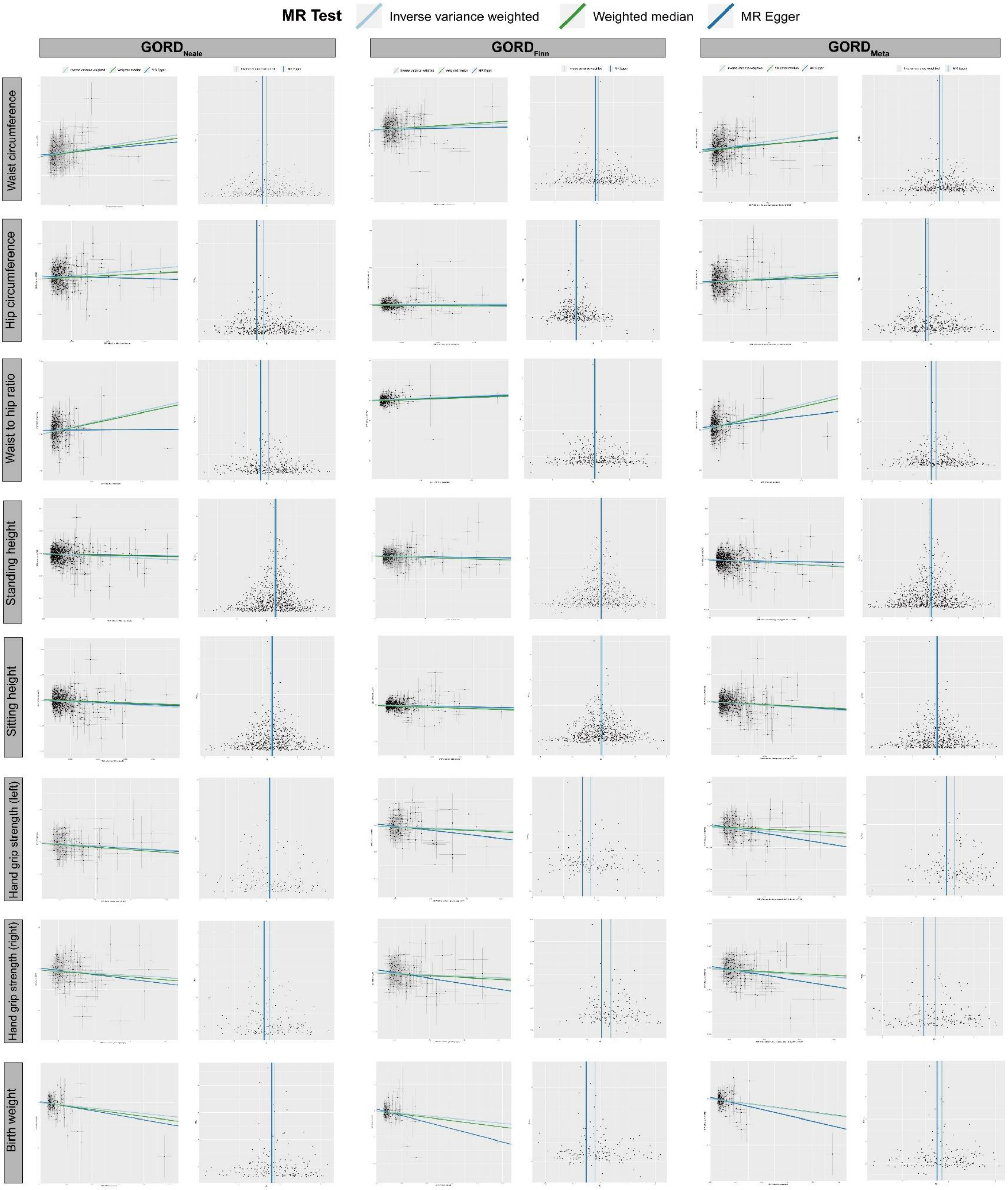

MR Test
Inverse variance weighted
Weighted median
MR Egger
GORD_Neale
GORD_Finn
GORD_Meta
Waist circumference
Hip circumference
Waist to hip ratio
Standing height
Sitting height
Hand grip strength (left)
Hand grip strength (right)
Birth weight

**Figure S4. Scatter plots and funnel plots for exposures (waist circumference, hip circumference, waist-to-hip ratio, height (standing and sitting), hand grip strength (left and right) and birth weight) for $GORD_{Neale}$, $GORD_{Finn}$ and $GORD_{Meta}$**

In scatter plot, X and Y axes respectively represented the genetic correlation of IVs (dots) on the exposures and outcomes, whose ratio was the slope of the slashes. The upward slashes represented the positive causal effects of exposures on the outcomes. On the contrary, the downward slashes represented the negative causal effects of exposures on the outcomes. In funnel plots, whether the distributions of IVs (dots) is symmetrical or not was used to judge the possible heterogeneity visually. MR-Egger regression was the main method to quantify the heterogeneity.

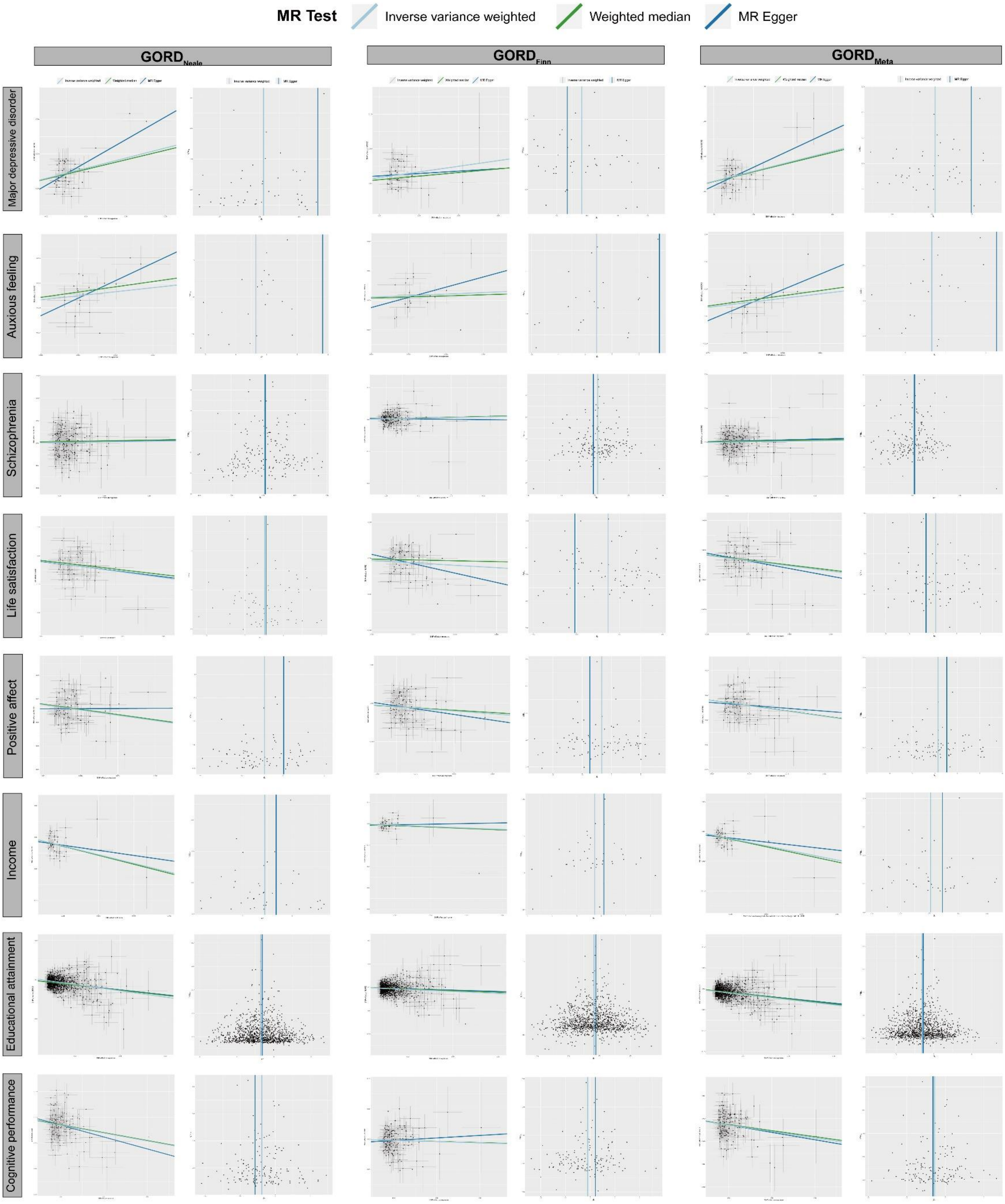

MR Test
Inverse variance weighted
Weighted median
MR Egger
GORD Neale
GORD Finn
GORD Meta
Major depressive disorder
Anxious feeling
Schizophrenia
Life satisfaction
Positive affect
Income
Educational attainment
Cognitive performance

**Figure S5. Scatter plots and funnel plots for exposures (major depressive disorder, anxious feeling, schizophrenia, life satisfaction, positive affect, income, educational attainment and intelligence) for $GORD_{Neale}$, $GORD_{Finn}$ and $GORD_{Meta}$**

In scatter plot, X and Y axes respectively represented the genetic correlation of IVs (dots) on the exposures and outcomes, whose ratio was the slope of the slashes. The upward slashes represented the positive causal effects of exposures on the outcomes. On the contrary, the downward slashes represented the negative causal effects of exposures on the outcomes. In funnel plots, whether the distributions of IVs (dots) is symmetrical or not was used to judge the possible heterogeneity visually. MR-Egger regression was the main method to quantify the heterogeneity.
